# Supplementary material for: In situ detection of the protein corona in complex environments
Source: Nat Commun. 2017 Nov 16;8:1542. doi: 10.1038/s41467-017-01826-4 (PMC5688064; doi:10.1038/s41467-017-01826-4)
Supplement: Supplementary file 1 — Supplementary Information [file 41467_2017_1826_MOESM1_ESM.pdf]

Supplementary Methods.

Ligand synthesis and characterization

All reagents were purchased from Sigma Aldrich and used without further purification. Polyethylene glycol (PEG) derivatives TrtS-PEG-OH (where Trt stands for trityl or triphenylmethyl), HS-PEG-COOH and TrtS-PEG-NH_2 contained a PEG fraction of approximately 3 kDa of molecular weight, and were purchased from Rapp Polymere (#133000-00-41, #133000-4-32 and #133000-20-41, respectively). Unless otherwise stated, reactions were run under air and non-anhydrous conditions. ^{1}H, ^{13}C, and ^{19}F-NMR spectra were recorded in deuterated chloroform ($CDCl_3$) solution in a Bruker 500 MHz spectrometer. Chemical shifts are reported in parts per million (ppm) relative to the residual signal of the solvent used, except in the case of ^{19}F-NMR spectra, which are referred to trifluoroacetic acid (TFA, δ = -76.55 ppm). Coupling constants (J) are expressed in Hertz (Hz). Drying of organic extracts after work-up of reactions was performed over anhydrous Na_2SO_4. Evaporation of solvents was accomplished with a Buchi rotary evaporator. Matrix-Assisted Laser Desorption/Ionization – Time of flight (MALDI-TOF) mass analyses of new compounds were performed on an Ultraflextreme III time-of-flight mass spectrometer equipped with a pulsed Nd:YAG laser (355 nm) and controlled by FlexControl 3.3 and FlexAnalysis 3.3 softwares (Bruker Daltonics, Bremen, Germany). For other measurements, time-of-flight mass spectrometer (ESI-TOF) LCT Premier XE from Waters (Milford, MA, USA) with an electrospray ionization source, working in positive / W mode was used.

Synthetic procedures

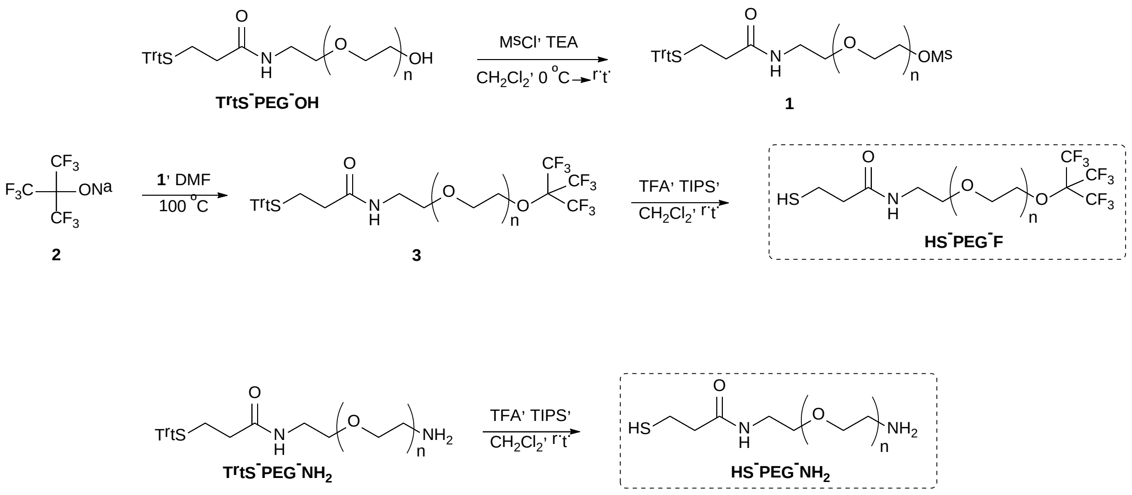

Supplementary Figure 1: Synthetic route to ligands HS-PEG-F and HS-PEG-NH_2.

Compound 1. *Typical procedure*. Commercially available TrtS-PEG-OH (400 mg, 0.121 mmol) was dissolved in CH_2Cl_2 (1.2 mL) and triethylamine (TEA, 98.3 µL, 0.71 mmol) was added. Subsequently, mesyl chloride (MsCl, 42.2 µL, 0.54 mmol) was added dropwise at 0 °C. The reaction was allowed to slowly reach room temperature (r.t.), and was stirred for 6 h since MsCl addition. The crude mixture was poured in a separation funnel and shook 4 times with a saturated solution of NH_4Cl, and then twice with brine. The organic layer was dried and the solvent removed in vacuo. Mesylated compound **1** was obtained as a pale yellow solid (409 mg, 99.9%) and was used without further purification in the next step. ^{1}H-NMR (500 MHz, $CDCl_3$) δ

7.32 (d, J = 7.7 Hz, 6H), 7.19 (t, J = 7.6 Hz, 6H), 7.11 (t, J = 7.3 Hz, 3H), 6.01 (t, J = 5.3 Hz, 1H), 4.32 – 4.24 (m, 2H), 3.55 (m, PEG), 3.28 (q, J = 5.3 Hz, 2H), 3.00 (s, 2H), 2.40 (t, J = 7.4 Hz, 2H), 1.97 (t, J = 7.4 Hz, 2H); ^{13}C-NMR (126 MHz, $CDCl_3$) δ 170.71, 144.50, 129.36, 127.69, 126.44, 70.41, 70.34, 70.24, 70.02, 69.55, 69.11, 68.79, 39.01, 37.51, 35.16, 27.50. HRMS (ESI): mass/charge (*m/z*) calculated for $C_{157}H_{291}NO_{70}S_2Na_3$: 1147.9455 $[M+3Na]^{3+}$; found 1147.9548.

Compound 3. *Typical procedure*. Mesylated compound **1** (296 mg, 0.087 mmol) was dissolved in DMF (1.75 mL) and **2** [1] was added (85 mg, 0.33 mmol). The mixture was stirred at 100 °C overnight. The crude mixture was allowed to reach r.t., poured in a separation funnel and shook 5 times with a saturated solution of NH_4Cl. The organic layer was dried and the solvent removed in vacuo. Compound **3** was obtained as a pale orange solid (281 mg, 91 %) and was used without further purification in the next step. ^{1}H-NMR (500 MHz, $CDCl_3$) δ 7.38 (d, J = 7.7 Hz, 6H), 7.24 (d, J = 7.8 Hz, 6H), 7.17 (t, J = 7.0 Hz, 2H), 6.06 (br s, 1H), 4.21 – 4.04 (m, 2H), 3.93 – 3.17 (m, PEG), 2.46 (t, J = 7.5 Hz, 2H), 2.02 (t, J = 7.6 Hz, 2H); ^{13}C-NMR (126 MHz, $CDCl_3$) δ 170.85, 144.62, 129.49, 127.81, 126.56, 120.21 (q, $J_{C\text{-}F}$ = 293.8 Hz), 83.71, 70.97, 70.46, 70.14, 69.72, 69.38, 69.30, 39.14, 35.30, 27.63. ^{19}F-NMR (470 MHz, $CDCl_3$) δ -71.48 (s). HRMS (ESI): *m/z* calculated for $C_{160}H_{292}F_9N_4O_{68}S$: 1189.6567 $[M+3NH_4]^{3+}$; found 1189.6462.

HS-PEG-F. *Typical procedure*. Compound **3** (218 mg, 0.062 mmol) was dissolved in CH_2Cl_2 (7.4 mL) and TFA (0.454 mL, 0.616 mmol) was added, followed by triisopropylsilane (TIPS, 0.624 mL, 0.308 mmol). Subsequently, TFA (0.454 mL, 0.616 mmol) and TIPS (0.624 mL, 0.308 mmol) were added again to the reaction mixture, and it was then allowed to stir for 15 min at r.t. All liquids were completely removed under high vacuum, and the so-obtained residue was triturated with diethyl ether (Et_2O) and filtered washing with Et_2O. Compound HS-PEG-F was obtained as a white solid (189 mg, 90%). To keep the product as a thiol and avoid disulfide formation, compound HS-PEG-F was stored at 4 °C under argon atmosphere. ^{1}H-NMR (500 MHz, $CDCl_3$) δ 6.77 (bs, 1H), 4.12 (t, J = 4.9 Hz, 2H), 3.75 (d, J = 4.3 Hz, 2H), 3.70 (t, J = 4.9 Hz, 2H), 3.61 – 3.53 (m, PEG), 3.46 (t, J = 4.9 Hz, 2H), 3.42 (q, J = 5.2 Hz, 2H), 2.77 (q, J = 7.2 Hz, 2H), 2.50 (t, J = 6.8 Hz, 2H). ^{13}C-NMR (126 MHz, $CDCl_3$) δ 171.12, 119.28 (q, $J_{C\text{-}F}$ = 298.7 Hz), 83.46, 70.90, 70.43, 70.39, 70.32, 70.22, 69.94, 69.75, 69.27, 40.03, 39.09, 20.44; ^{19}F-NMR (470 MHz, $CDCl_3$) δ -71.48 (s). HRMS (MALDI): *m/z* calculated for $C_{141}H_{274}F_9NO_{68}SNa$: 3295.7483 $[M+Na]^+$; found, 3295.7485.

HS-PEG-NH₂. *Typical procedure*. Commercially available compound TrtS-PEG-NH_2 (101 mg, 0.031 mmol) was dissolved in CH_2Cl_2 (3.7 mL) and TFA (0.223 mL, 0.303 mmol) was added, followed by TIPS (0.307 mL, 0.152 mmol). Subsequently, TFA (0.223 mL, 0.303 mmol) and TIPS (0.307 mL, 0.152 mmol) were added again to the reaction mixture and it was then allowed to stir for 15 minutes at r.t. All liquids were completely removed under high vacuum and the so-obtained residue was triturated with Et_2O and filtered washing with Et_2O. Compound HS-PEG- NH_2 was obtained as a white solid (94 mg, 99.9%). To keep the product as a thiol and avoid disulfide formation, compound HS-PEG-NH_2 was stored at 4 °C under argon atmosphere. ^{1}H-NMR (500 MHz, $CDCl_3$) δ 6.44 (s, 1H), 5.79 (s, 2H), 3.80 (t, J = 5.2 Hz, 2H), 3.76 (t, J = 4.8 Hz, 2H), 3.75 – 3.52 (m, PEG), 3.48 (t, J = 5.0 Hz, 2H), 3.44 (q, J = 5.1 Hz, 2H), 3.18 (q, J = 5.6 Hz, 2H), 2.79 (q, J = 7.2 Hz, 2H), 2.50 (t, J = 6.8 Hz, 2H). Alternatively, compound HS-PEG- NH_2 can also be purchased in Rapp polymere (#133000-40-20).

Supplementary Figures.

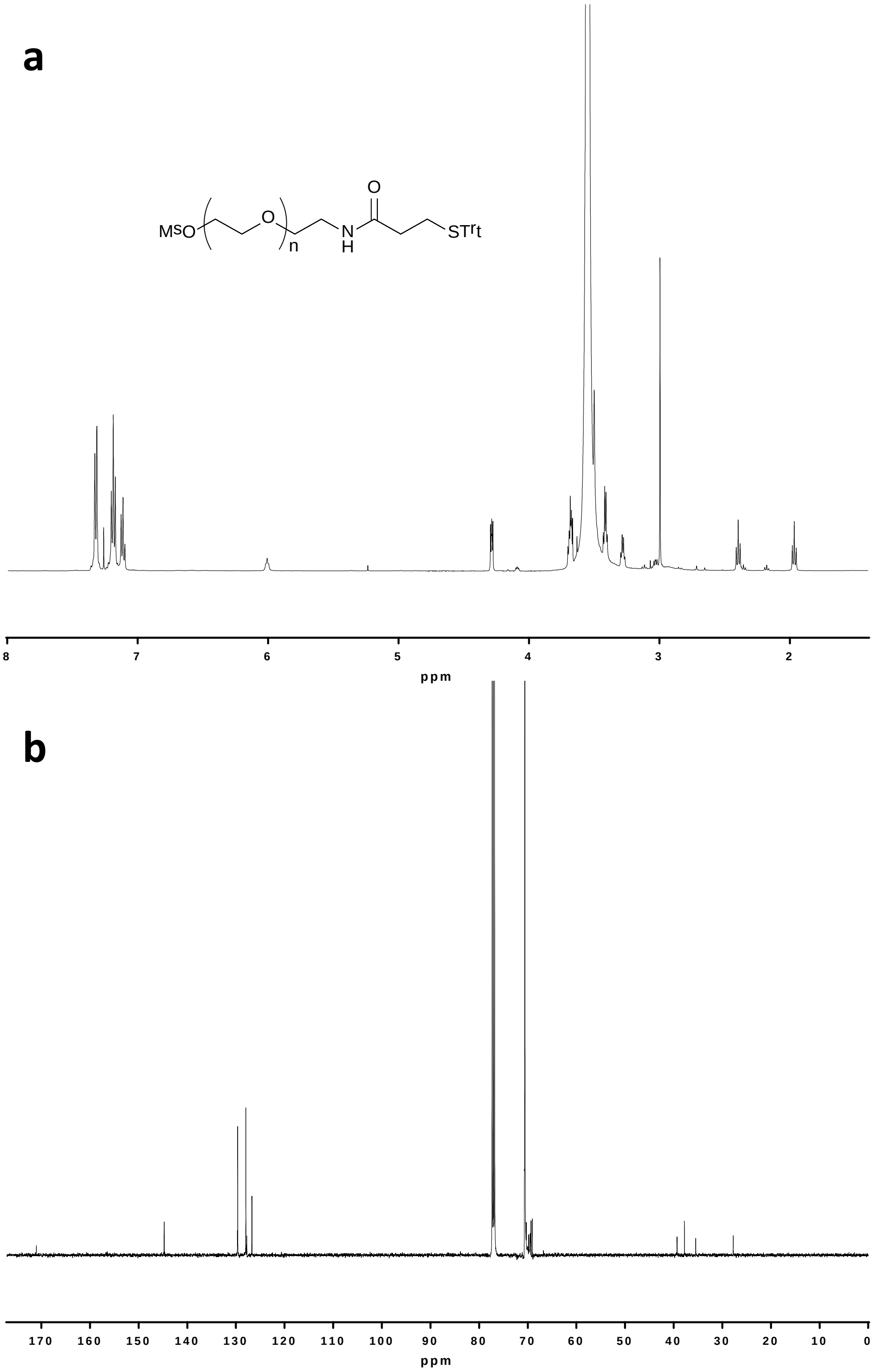

Supplementary Figure 2: NMR spectra of compound **1.** **a** ^{1}H-NMR and **b** ^{13}C-NMR.

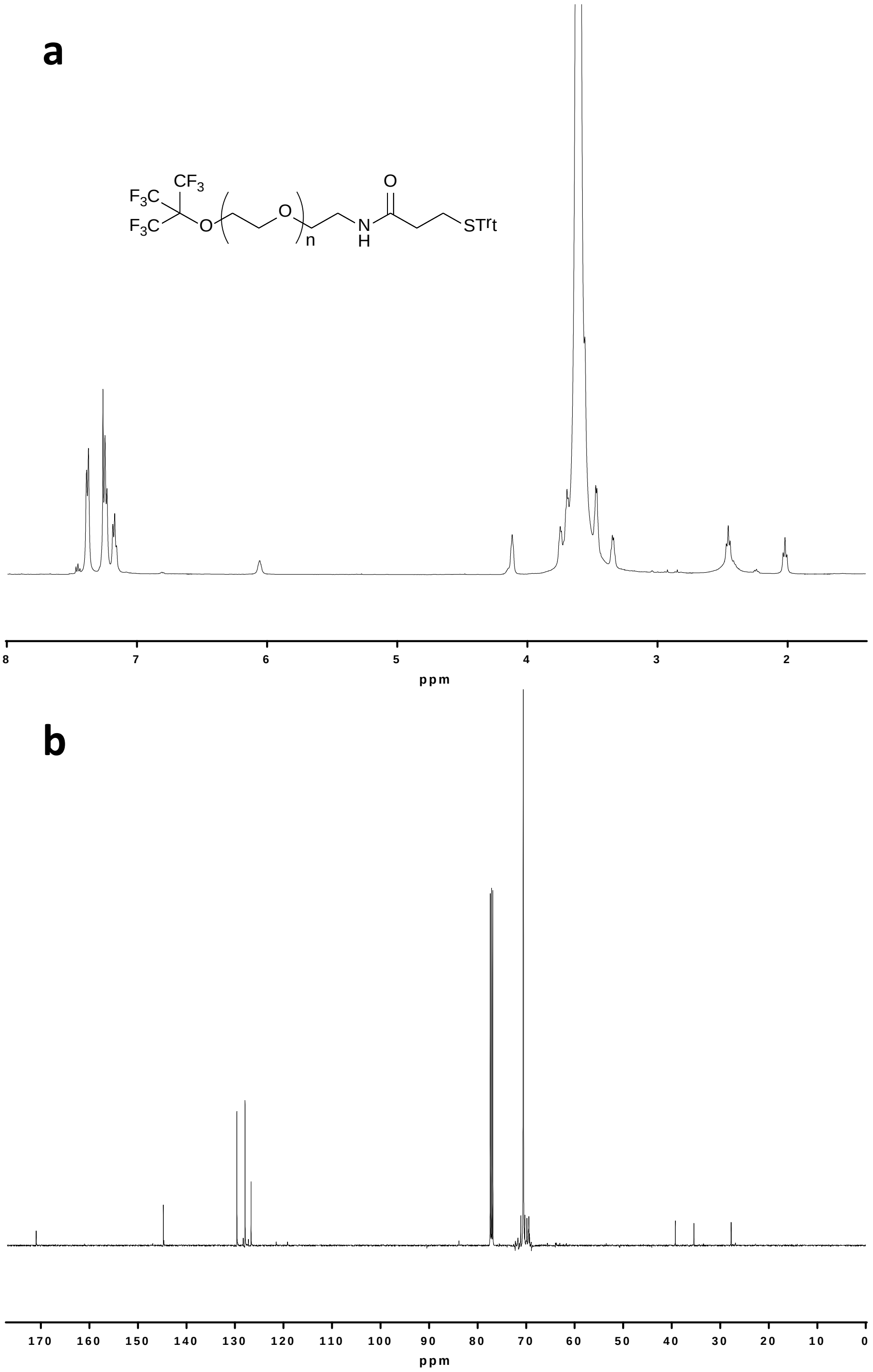

Supplementary Figure 3: NMR spectra of compound **3.** **a** ^{1}H-NMR and **b** ^{13}C-NMR.

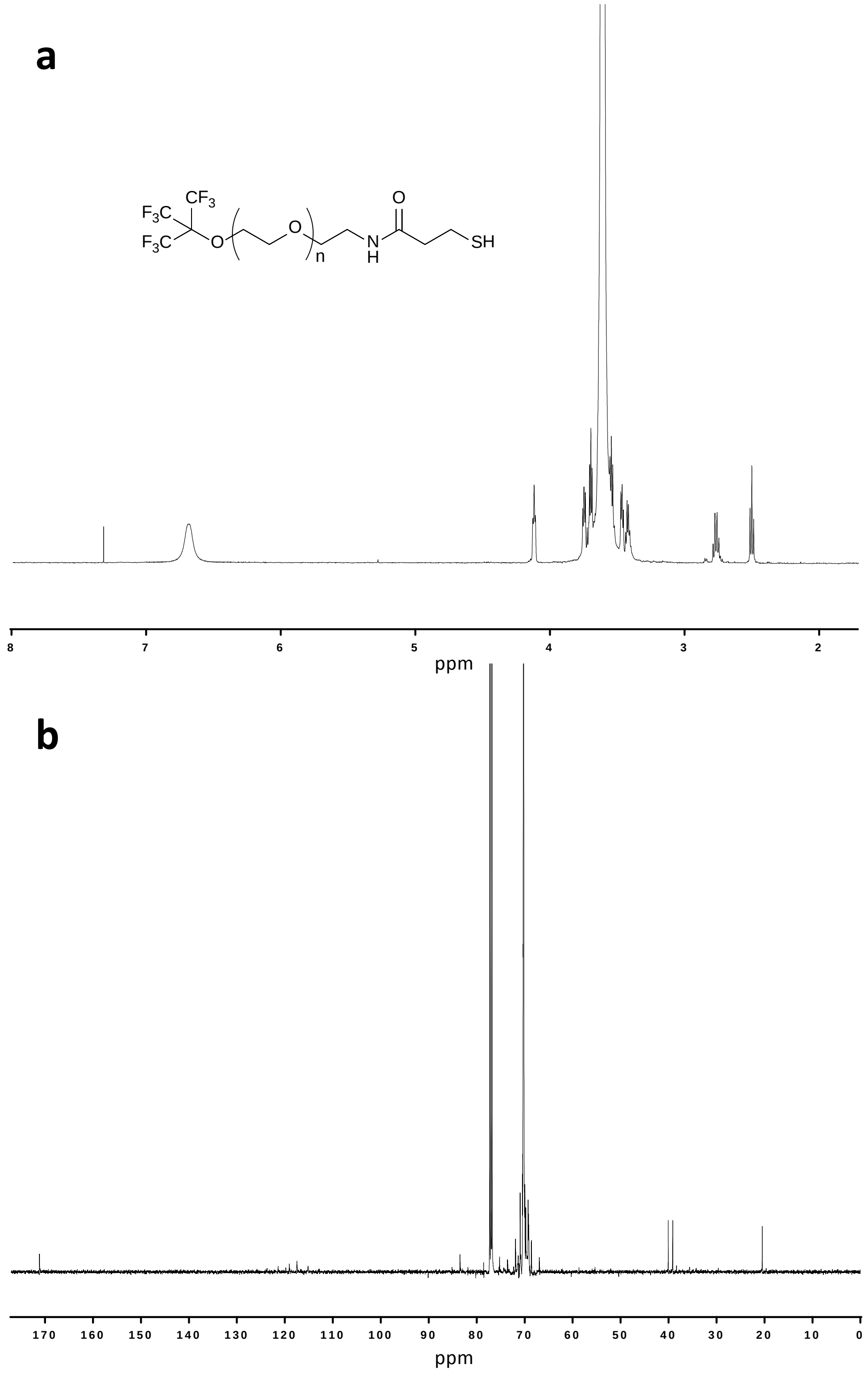

Supplementary Figure 4: NMR spectra of **HS-PEG-F. a** ^{1}H-NMR and **b** ^{13}C-NMR.

Gold NP synthesis

Tetrachloroauric(III) acid ($HAuCl_4$) was purchased from Strem Chemicals (#79-0500) and sodium borohydride ($NaBH_4$) from Sigma Aldrich (#213462). Solvents (analysis quality) were purchased from Panreac and used as received. Water is MilliQ quality. A Sigma bench centrifuge was used for centrifugations at speed lower than 14 x 10^3 g (relative centrifugal force). A Beckmann Coulter floor ultracentrifuge was used for centrifugations at speed greater than 5 x 10^4 g. In total three different types of Au nanoparticles (NPs) were prepared, as shown in Supplementary Figure 5.

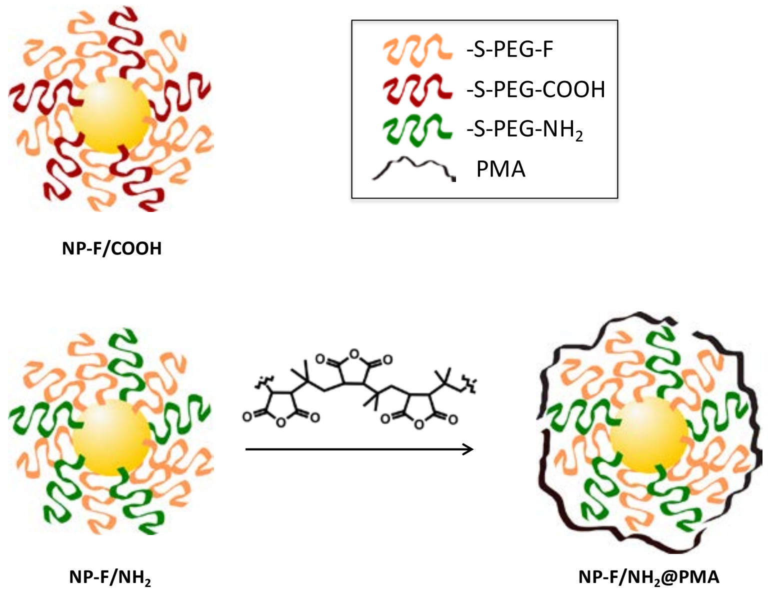

Supplementary Figure 5: Schematic representation of synthesized NPs.

Synthesis of NP-F/COOH and NP-F/NH₂

Typical procedure: HS-PEG-F ligand (844 µL of a 2.5 mM solution in CH_2Cl_2) and either HS-PEG-COOH or HS-PEG-NH_2 (281 µL of a 2.5 mM solution in CH_2Cl_2) were added to 50 mL of CH_2Cl_2 while stirring at r.t. Subsequently, a 0.1 M solution of $NaBH_4$ was freshly prepared with ice-cold methanol and allowed to stand for 1 min. During that minute, a freshly prepared solution of $HAuCl_4$ (250 µL, 25 mM) in ice-cold methanol was added to the reaction mixture. One minute after the preparation of $NaBH_4$ solution, the latter (313 µL, 0.1 M) was added dropwise to the ligand/gold mixture. The reaction was stirred for 30 s and then allowed to stand overnight without stirring at r.t. Hereby the solution was capped to avoid solvent evaporation. On next day, the solvent was evaporated in a rotavapor, and the NPs were resuspended in water and then purified by centrifugation. Firstly, NPs were centrifuged twice at 1.5 x 10^5 g for 2 h to remove unbound ligand and very small NPs, which remained in the supernatant and were discarded. Secondly, the so-obtained pellet was resuspended in water and centrifuged twice at 5 x 10^4 g for 30 min to remove aggregates and bigger NPs that precipitated at that speed and were discarded. Hence, NPs that remained in the supernatant were kept for further experiments (see Supplementary Figure 6).

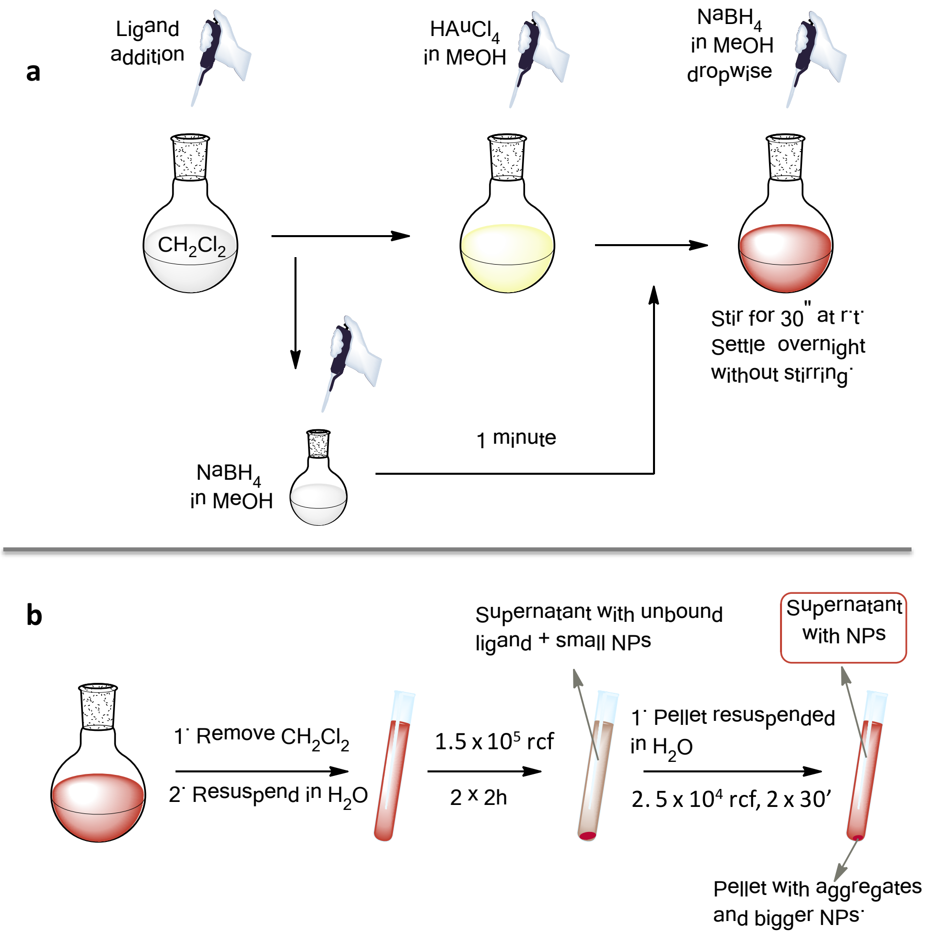

Supplementary Figure 6: Schematic representation of NP-F/COOH and NP-F/NH_2. **a** synthetic protocol and **b** purification by centrifugation. Templates provided by ChemBioDraw have been used in this Figure.

Synthesis of NP-F/NH_2@PMA

Typical procedure. NP-F/NH_2 (0.87 nmol) was dried under high vacuum to remove all water molecules and resuspended in 6.48 mL of CH_2Cl_2 in a glass vial under stirring at r.t. A poly(isobutylene–*alt*-maleic anhydride) (PMA) solution in tetrahydrofuran (THF, 9.2 mL, 17.5 mM in monomers) was added onto the NPs, followed by 4-(dimethylamino)pyridine (DMAP) (95 µL, 1.5 M in CH_2Cl_2) as a catalyst for the amide bond formation through the reaction between cyclic anhydrides on PMA and amine moieties on the NPs (Supplementary Figure 7**a**). The vial was capped and the mixture was allowed to stir overnight at r.t. Next morning, the NPs were extensively washed with THF by centrifugation to remove unbound PMA, which remained in the supernatant dissolved in THF and was discarded (4 times at 2 x 10^3 g during 4 min). Subsequently, the NPs obtained as a pellet after centrifugation were treated with 0.5 mL of NaOH (0.1 M) followed by 1 mL of NaOH (5 M) to transform all anhydride moieties present in PMA into the corresponding carboxylates [2]. The so-obtained NPs were further centrifuged twice at 125 x 10^3 g for 45 min using PBS (phosphate buffered saline solution, pH=7.4) as the washing solution. The supernatant was discarded and the so-obtained pellet of NPs was filtered through a 0.2 µm hydrophilic PTFE filter to remove aggregates (Supplementary Figure 7**b**).

For batch-to-batch reproducibility of the degree of PMA coating it is important to maintain a PMA monomer amount of at least 0.33 mmol/m^2 of NP-F/NH_2 surface, considering the hydrodynamic surface A_h derived by the hydrodynamic radius r_h in water: $A_h = 4\pi \cdot r_h^2$. The total NP surface of all NPs is $A_{h,tot} = A_h \cdot N_{NP}$, scaled with the number of NPs.

For the particular batch and example presented herein, the PMA/surface ratio has been calculated as described in the following:

The number of NPs per reaction is $N_{NP} = n_{NP} \cdot N_A = 8.7 \cdot 10^{-10}$ mol x $6.023 \cdot 10^{23}$ mol^{-1} = $5.24 \cdot 10^{14}$

From ^{19}F diffusion measurements the hydrodynamic radius of this particular batch of NP-F/NH_2 has been determined to be r_h = 8.57 nm, hence:

$A_{h,tot}$ = 4 x π x $(8.57 \cdot 10^{-9}$ m$)^2$ x $5.24 \cdot 10^{14}$ = $4.84 \cdot 10^{-1}$ m^2 total surface of all NPs per reaction

The total amount n_P of PMA monomers added to the reactions was:

$n_P = V \cdot c_P$ = 9.2 mL x $1.75 \cdot 10^{-2}$ M = $1.61 \cdot 10^{-1}$ mmol of PMA monomers per reaction

Hence, the used ratio between PMA monomers and NP surface was:

$n_P / A_{h,tot} = 1.61 \cdot 10^{-1}$ mmol/ $4.84 \cdot 10^{-1}$ m^2 = 0.33 mmol/m^2

In this manner it is possible to account for batch to batch size variability in the starting NP-F/NH_2 and keep the PMA coating degree.

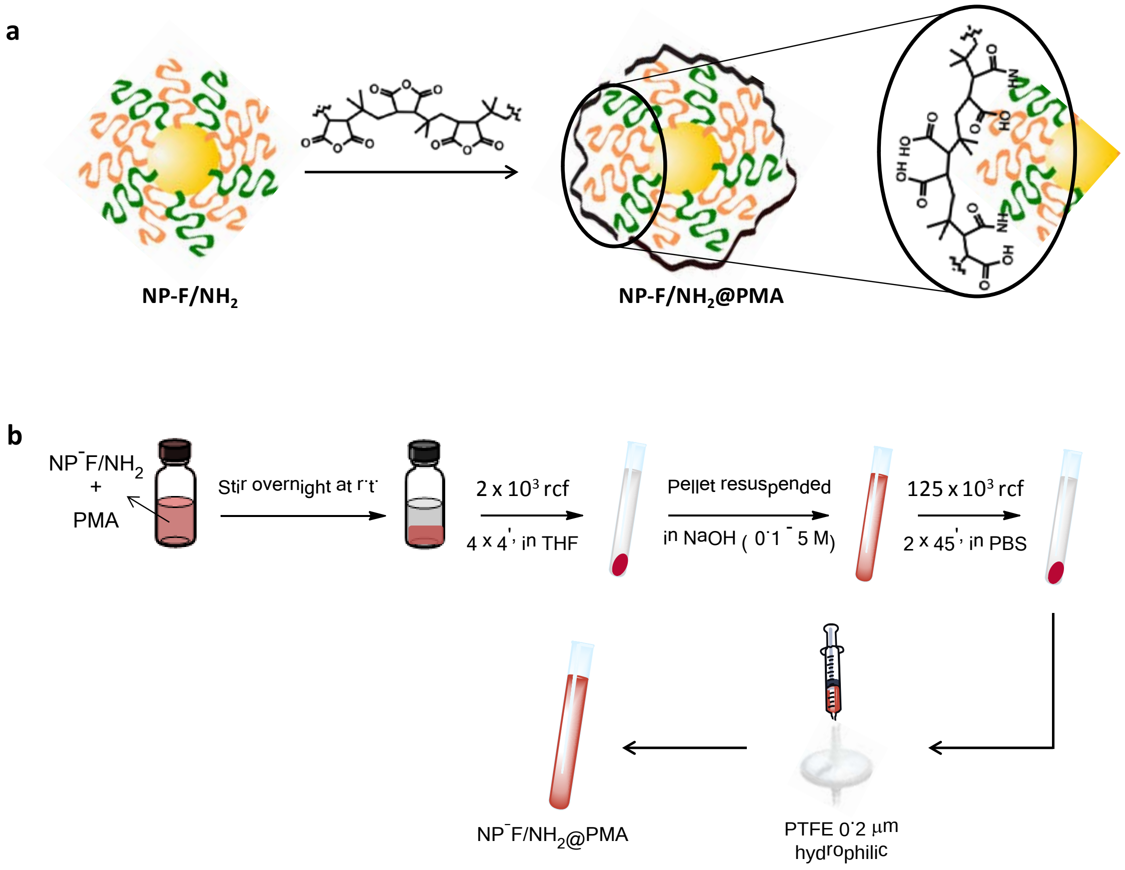

Supplementary Figure 7: **a** Schematic illustration of the reaction between PMA and the amine moieties on the surface of NP-F/NH_2 leading to amide bond formation. **b** Schematic representation of NP-F/NH_2@PMA synthetic protocol. Templates provided by ChemBioDraw have been used in this Figure.

Transmission Electron Microscopy (TEM)

Inorganic core radii (r_C) of all types of gold NPs were obtained from analysis of TEM images. As detailed elsewhere[3], a sample droplet was deposited on top of a carbon coated grid (Electron Microscopy Sciences, USA-Hatfield, PA) and allowed to dry overnight. Experiments were performed in a JEM-1230 transmission electron microscope running at 120 kV with a LaB_6 cathode and equipped with an ORIOUS SC1000 4008×2672 pixels CCD camera (Gatan UK, Abingdon Oxon, UK). Size measurement was performed with the free software ImageJ[4,5] and size distribution analysis and histogram production was done with Origin software. After TEM analysis, the following core radii were obtained for each type of NP: NP-F/COOH r_C = 1.5 ± 0.7 nm; NP-F/NH_2 r_C = 1.7 ± 0.6 nm; NP-F/NH_2@PMA r_C = 1.7 ± 0.6 nm (Supplementary Figure 8). The organic ligand shell does not provide sufficient contrast and thus is not included in the core diameter.

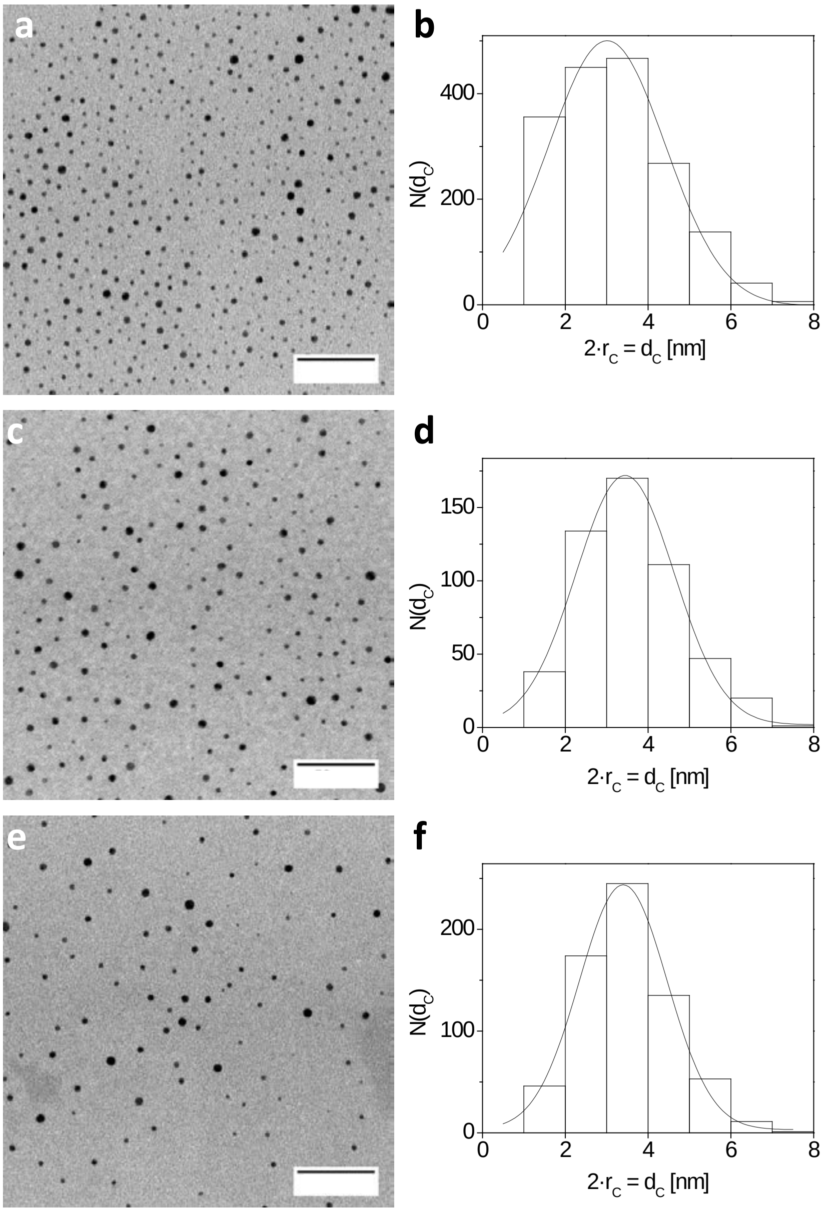

Supplementary Figure 8: Selected TEM micrographs (scale bar, 50 nm) and histograms for size distribution of the Au NP radius without organic shell (r_c) for NP-F/COOH **a**-**b**; NP-F/NH_2 **c**-**d**; NP-F/NH_2@PMA **e-f**. Histograms were obtained after analysis of at least 500 NPs. The core radius r_c is half the core diameter d_c.

Negative staining

By using negative staining techniques the organic ligand shell becomes visible in TEM [3]. The radii of the NP-organic shell system (r_{CS}) of NP-F/COOH, NP-F/NH_2 and NP-F/NH_2@PMA were analyzed by negative staining TEM analysis. For the staining, uranyl acetate purchased from Electron Microscopy Science and water of LC/MC purity from Fisher Scientific were used. Addition of a heavy element solution outlines the presence of lighter elements present in the sample. For this work, samples were prepared on glow discharged carbon film 400 copper mesh grids from Electron Microscopy Sciences (Hatfield, USA). A MED 020 modular high vacuum coating system (BAL-TEC AG, Balzers, Fuerstentum Liechtenstein) was used for glow discharge treatment of the grids ($2.0 \cdot 10^{-1}$ atm, 40 mA for 20 s in air). This grid treatment makes the carbon grid hydrophilic, favoring a rapid and more uniform spread of an aqueous droplet onto it. Analogous to previously reported sample preparation methodology [3], a sequential two-droplet method was applied for the preparation of the samples. In detail, 0.35 µL of sample (NP concentrations of 0.2 µM for NP-F/COOH and NP-F/NH_2@PMA, and 0.1 µM for NP-F/NH_2) were immediately added to the hydrophilic grid, incubated for 30 s, and followed by the addition of 0.35 µL of 0.25 % w/v uranyl acetate solution.

The image analysis of several negative staining TEM micrographs of NP-F/COOH, NP-F/NH_2 and NP-F/NH_2@PMA was done. For morphological characterization of NP-organic shell system, the borders between the organic shells and the stained grid were automatically segmented by ImageJ[4,5] (function Analyzed Particles), *cf.* Supplementary Figure 9, Supplementary Figure 10 and Supplementary Figure 11 for NP-F/COOH, NP-F/NH_2 and NP-F/NH_2@PMA, respectively. The total area limited by the outlines was divided by the total number of NPs analyzed, to give a spherically-idealized mean area occupied by the NP-organic shell system (A_{CS}), from which the mean r_{CS} can be extracted (*i.e.*, $r_{CS}=\sqrt[2]{A_{CS}/\pi}$).

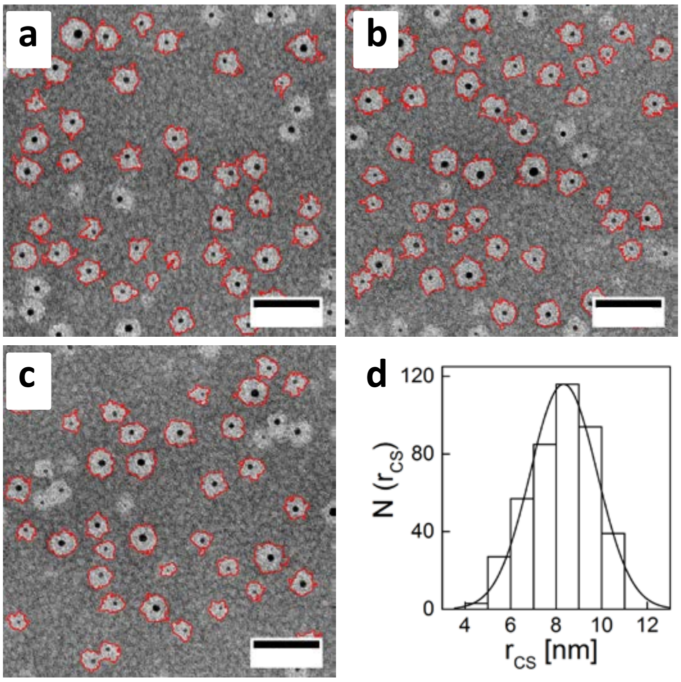

Supplementary Figure 9: **a-c** Selected low magnification negative staining TEM micrographs (scale bar, 50 nm) obtained with the sample NP-F/COOH, and **b** the histogram of the corresponding size distribution (r_{cs} = 8.3 ± 1.4 nm, as obtained from > 400 NPs). Red outlines correspond to the NPs analyzed in these images.

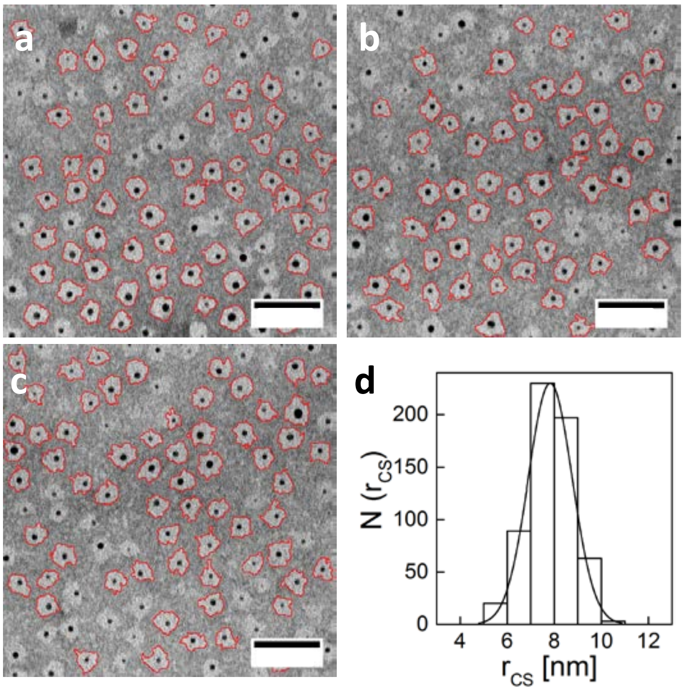

Supplementary Figure 10: **a-c** Selected low magnification negative staining TEM micrographs (scale bar, 50 nm) obtained with the sample NP-F/NH_2, and **b** the histogram of the corresponding size distribution (r_{cs} = 7.8 ± 0.9 nm, as obtained from >300 NPs). Red outlines correspond to the NPs analyzed in these images.

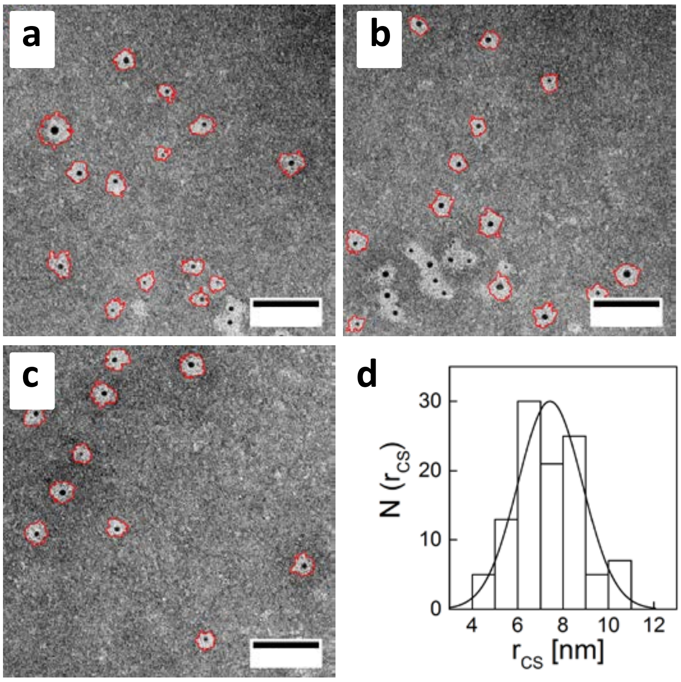

Supplementary Figure 11: **a-c** Selected low magnification negative staining TEM micrographs (scale bar, 50 nm) obtained with the sample NP-F/NH_2@PMA, and **b** the histogram of the corresponding size distribution (r_{cs} = 7.5 ± 1.2 nm, as obtained from >300 NPs). As the poor contrast of the micrographs obtained for this sample did not allow us to run a fully automated image analysis (only in *ca.* 150 NPs), for the rest of the NPs the outlines had to be drawn manually, and the obtained data for r_{cs} can only be taken as an approximation. Red outlines correspond to the NPs analyzed in these images.

Ultraviolet – Visible absorption spectra measurement (UV-Vis)

UV-Vis absorption spectra of all NPs were recorded in water in a Varian spectrometer, and the absorption A(λ) was normalized at λ = 450 nm. As expected, the UV-Vis absorption spectrum of NP-F/NH_2@PMA remained unchanged when compared with parent NP-F/NH_2 after PMA coating. The maximum absorption for NP-F/COOH was at λ = 509 nm and for NP-F/NH_2 and NP-F/NH_2@PMA was at λ = 512 nm (Supplementary Figure 12).

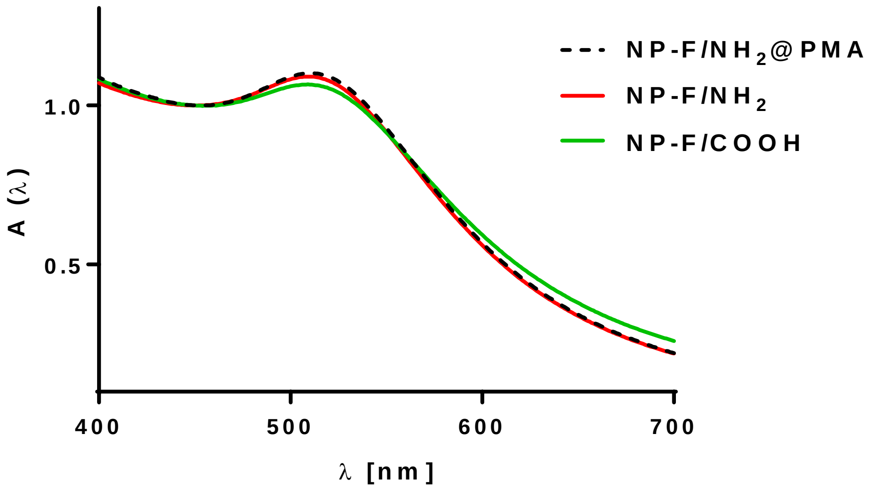

Supplementary Figure 12: UV-Vis absorption spectra of NP-F/COOH, NP-F/NH_2 and NP-F/NH_2@PMA measured in water. A(λ) is normalized at λ = 450 nm.

Inductively coupled plasma mass spectrometry (ICP-MS)

The content of elemental Au in NP solutions was obtained by ICP-MS (Agilent 7700 Series). The samples were dried prior to measurement in 15 mL glass vials to ensure safe transport and prohibit any interference from the solution medium. The dried samples were weighted to 1.1 - 1.5 mg per vial prior to digestion, in order to determine the overall necessary volume of aqua regia. Following these weightings the samples were digested directly inside the glass vials using 1.2 mL of aqua regia consisting of 1 part HNO_3 (67 wt%, ultra pure, Fisher Chemical, #7697372) and 3 parts HCl (35 wt%, ultra pure, Fisher Chemicals, #7647010) to bypass any additional weighting steps or volume changes due to transfer of the sample. The samples were then shaken under constant agitation for 4 hours at r.t. During this period the dried elemental gold bulk was digested and broken down to much smaller gold acid rests. No complete digestion into atoms is needed, as small enough gold clusters can be easily ionised with ICP-MS. 200 µL of the digested gold solution was then transferred to a 6 mL perfluoroalkoxy alkane tube (PFA) and diluted using 1.8 mL of a 2 wt% HCl solution to prevent digestion of the machinery, as well as to provide an ion-stable environment with constant background conditions for all samples. The dilution factor for the digestion solution is therefore 10, whereby the actual gold content can be determined by the used volume as well as by the concentrations obtained by the ICP-MS. Measurements were done using 5 repetitions per sample, 100 sweeps, and a peak pattern of 3 peaks. The diluted samples were introduced to the ICP-MS set-up through an integrated autosampler, coupled to a Peltier cooling spray chamber, where the sample was nebulized and taken up by the argon gas flow at a speed of ½ m/s. All data given were calculated as the mean of all 5 measurements taken for each sample.

According to these measurements, the gold content (as determined in terms of elemental Au by ICP-MS) was found to be 26.64% and 28.40% of the total mass (of the weighted NP powder) for NP-F/COOH and NP-F/NH_2, respectively. The molar concentration values (c_{NP} [µM]) of the NPs'

aqueous solutions were calculated by using the inorganic core diameters obtained by TEM (d_C), the density of bulk gold (ρ =19.3 g/cm^3), and the ICP-MS data. From the core diameter and the density of bulk gold the mass of Au in one Au NP was calculated. ICP-MS data yielded in the mass of elemental Au per volume of solution. The molar mass of one Au NP was assumed to be the molar mass of the Au core, neglecting the mass contribution of the organic surface coating. With these data the concentration of the Au NPs c_{NP} was calculated [6].

In addition, since we know the percentage of gold in the NP powder sample, the remaing mass corresponds to ligands on the surface, that is 73.36% and 71.60% of the total mass for NP-F/COOH and NP-F/NH_2, respectively. With these values, knowing the ligand ratio and the molecular weight of the ligands it is possible to calculate the number of ligands on each NP. The r_c data obtained from TEM allow the calculation of one NP surface and combining all data we obtained a value of 4.8 and 5.1 ligand per nm^2 for NP-F/COOH and NP-F/NH_2, respectively.

UV-Vis absorption spectra of NP solution of increasing NP concentration were measured and the absorbance of each sample at 450 nm (A_{450}) was plotted against the NP (c_{NP}) concentration value according to ICP-MS data, to find a linear relationship. These graphs were used for calculating the concentration of NP solutions from their UV-Vis absorption at 450 nm (Supplementary Figure 13). TEM analysis and UV-Vis absorption spectra of NP-F/NH_2@PMA showed that the size of the core remained unchanged (*cf.* Supplementary Figures 8 and 12) in comparison with parent NP-F/NH_2, for which the same calibration curve was used for calculating the concentration of both, NP-F/ NH_2 and NP-F/NH_2@PMA.

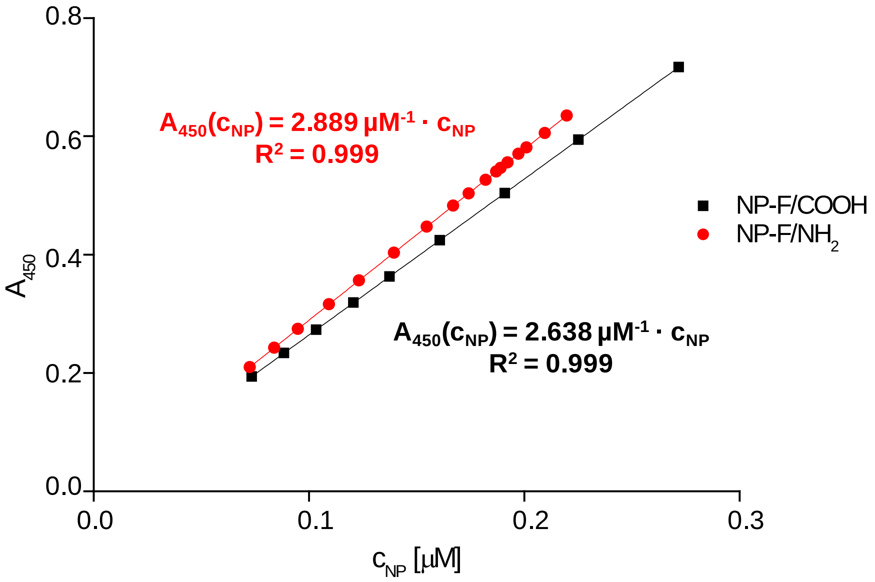

Supplementary Figure 13: Correlation between NP concentration c_{NP} obtained by ICP-MS analysis and UV-Vis absorption A_{450} at λ = 450 nm. Red dots refer to NP-F/NH_2 and black dots refer to NP-F/COOH. Measurements were carried out in water in a cuvette with 1 cm pathlength. Data points were fitted with linear regression analysis. Obtained fit parameters are given in the image. R square (R^2) is the coefficient of determination of the linear regression fit, which is an indication of the quality of the fit.

ζ potential measurement

The zeta potential values of all three types of NPs were recorded in water in a Zetasizer Nano ZS (Malvern, Worcesterhire, UK) equipped with a 633 nm laser, using NP concentrations of $c_{NP} \approx 20$ nM. The distributions N(ζ) of obtained zeta potentials are depicted in Supplementary Figure 14 along with the values, which vary accordingly with the presence of carboxylic or amino groups from negative to neutral or slightly positive.

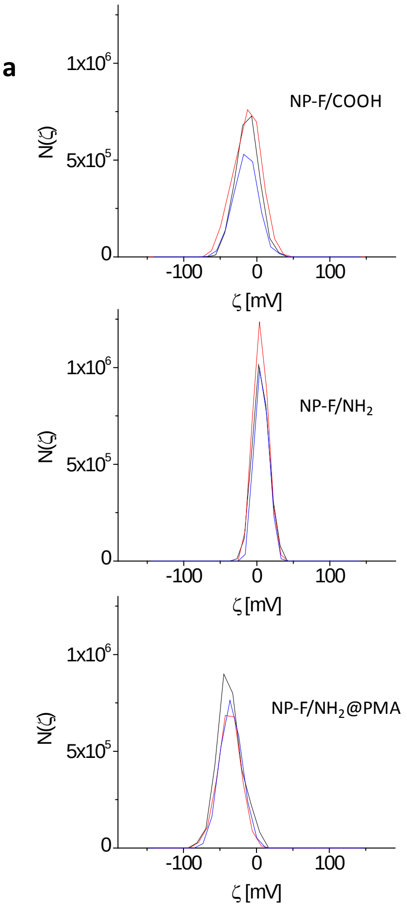

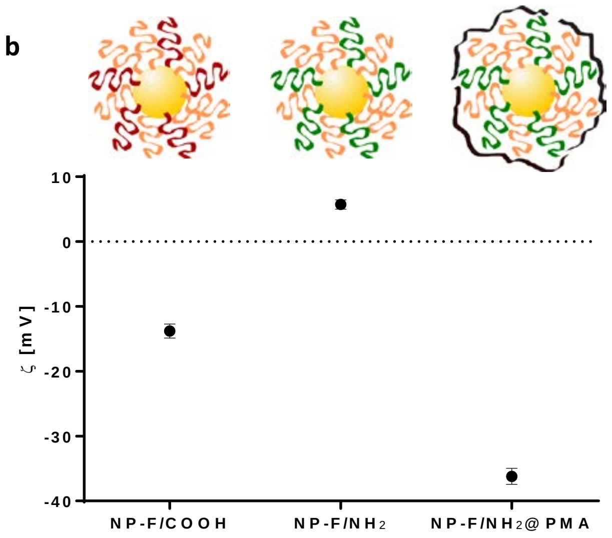

c

NP type	ζ [mV]
NP-F/COOH	-13.8 ± 1.1
NP-F/NH_2	5.7 ± 0.7
NP-F/NH_2@PMA	-36.2 ± 1.2

Supplementary Figure 14: **a** Zeta potential distributions N(ζ) for each type of NP as recorded in water. **b** Graphical representation of zeta potential values obtained for each NP. **c** Table with the average zeta potential values for each sample with the standard deviation (SD) corresponding to at least 3 measurements.

Interfacial Tension (IFT) measurement

Pendant drop tensiometry was used to investigate the dynamic interfacial tension (IFT) of NP-F/COOH, NP-F/NH_2 and NP-F/NH_2@PMA[7]. Profile changes of a sample droplet immersed in toluene were recorded using a drop Shape Analysis System (DSA100, Krüss, Germany). An ultra fast camera (Krüss, Germany) was used to image the time-dependent changes in drop shape. At room temperature, 50 µL sample droplet (NP concentration of c_{NP} = 120 nM) was dispensed with a Hamilton syringe plugged to a stainless steel needle at 200 $\mu L \cdot min^{-1}$ dosing rate. The interfacial tension (γ_t) was estimated fitting the droplet profile with the Young-Laplace equation using the analysis software package DSA3 (Krüss, Germany). The droplet profile was recorded and fitted

every 15.15 s, obtaining a time dependent variation of the interfacial tension. Each interfacial tension measurement was plotted and fitted to the empirical Hua and Rosen equation (Supplementary Equation 1)[8]

$$\gamma_t = \gamma_m + \frac{\gamma_0 - \gamma_m}{1+(t/t^*)^n} \qquad \text{Supplementary Equation 1}$$

where γ_t is the surface tension at any time t, γ_0 is the surface tension of the pure solvent, γ_m is the surface tension at mesoequilibrium, t^* is the half-time in reaching γ_m, and n is a dimensionless exponent. Assuming a constant value of γ_0 = 36 mN/m for the surface tension value of the water-toluene interface, γ_m, t^*, and n remain as fitting parameters. These parameters were estimated by computer fitting using Origin Lab ® software. The mean values and SD for each type of NP obtained after 3 independent measurements were 15.53 ± 0.37 mN/m, 13.73 ± 0.91 mN/m, and 18.37 ± 0.69 mN/m for NP-F/COOH, NP-F/NH_2, and NP-F/NH_2@PMA, respectively (Supplementary Figure 15).

a

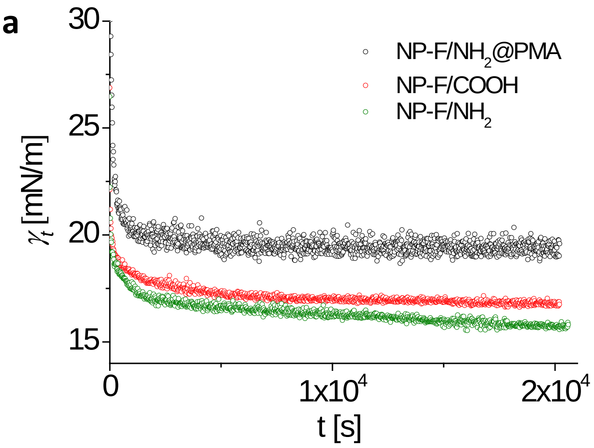

b

NP type	γ_m [mN/m]
NP-F/NH_2@PMA	18.37 ± 0.69
NP-F/COOH	15.53 ± 0.37
NP-F/NH_2	13.73 ± 0.91

Supplementary Figure 15: **a** Selected dynamic surface tension (γ_t) plots for all three types of NPs, where the interfacial tension decreases with time, approaching an equilibrium value. **b** Table with the average γ_m values for each type of NP with the standard deviation (SD) corresponding to 3 measurements.

^{1}H nuclear magnetic resonance (^{1}H-NMR) spectra measurement

^{1}H-NMR spectra of the supernatants above purified NPs were recorded in order to confirm the ratio of the ligands. The recovered supernatant from ultracentrifugation at 150.000 g containing the unbound ligands were lyophilized and dissolved in $CDCl_3$ for ^{1}H-NMR analysis. To obtain the ligand ratios we chose characteristic signals from each type of ligand that are exclusive to them. For instance, for HS-PEG-F we chose the signature methylene at 4.12 ppm, which is the closest to the fluorinated moiety; for HS-PEG-COOH we chose the signature of methylene at 1.91 ppm, which is the second closest to the COOH group, and for HS-PEG-NH_2 we chose the signature of methylene at 3.18 ppm, which is the closest to the NH_2 group. Since the molecular weights of these PEGylated ligands are very similar we obtained the ratio between them by direct integration of the selected signals on the spectra. By comparing the integration ratios for those signals we observed that the 75/25 ratio between HS-PEG-F and either HS-PEG-COOH or HS-

PEG-NH_2 was roughly maintained after NP synthesis, being 72/28 for NP-F/COOH and 73/27 for NP-F/NH_2 (Supplementary Figure 16).

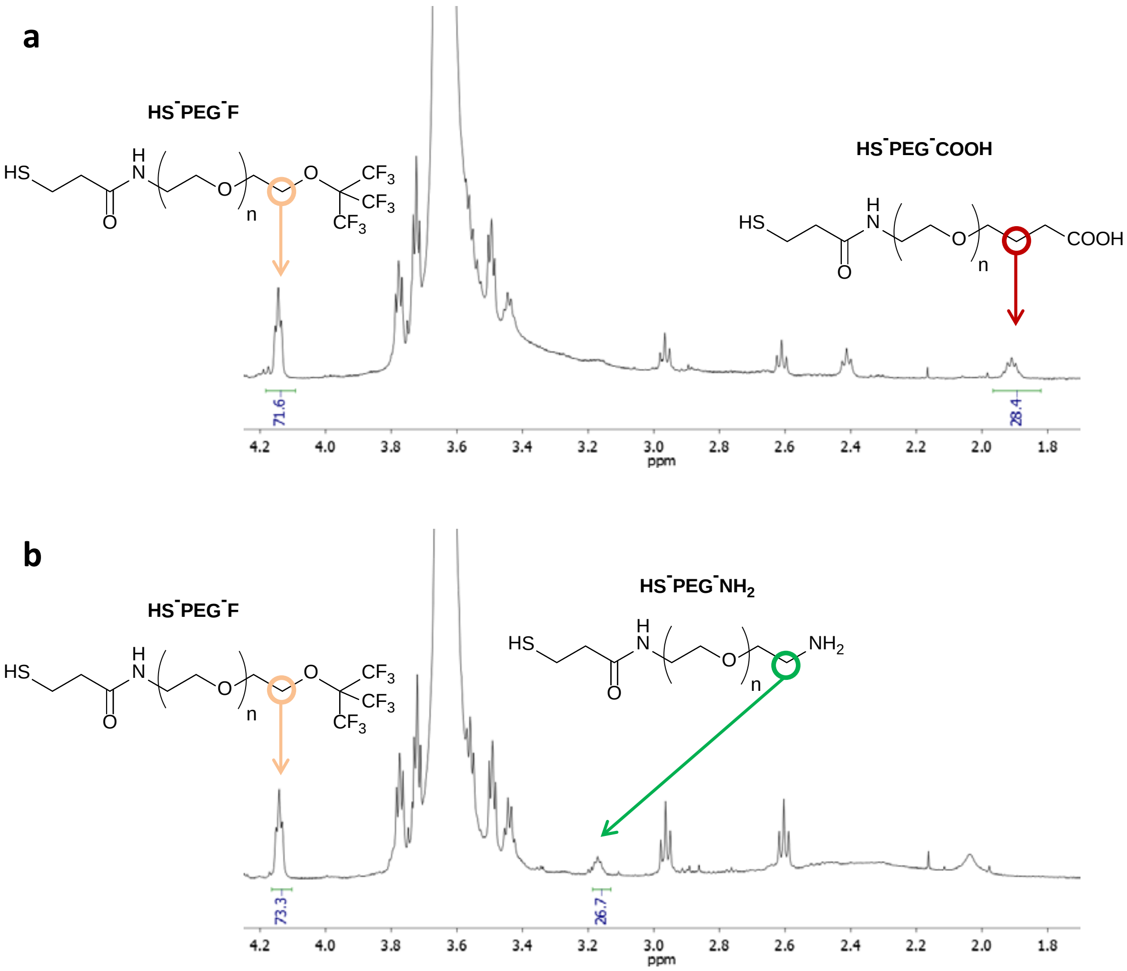

Supplementary Figure 16: **a** ^{1}H-NMR of the recovered unbound ligand mixture after the synthesis of NP-F/COOH with the integrated signals showing the ligand ratio. **b** ^{1}H-NMR of the recovered unbound ligand mixture after the synthesis of NP-F/NH_2 with the integrated signals showing the ligand ratio.

^{19}F nuclear magnetic resonance (^{19}F-NMR) spectra measurement

^{19}F-NMR spectra of all NPs were measured in a mixture of water and deuterium oxide (H_2O:D_2O 85:15) and compared with that of the starting ligand HS-PEG-F before being grafted onto NPs. A single peak was obtained for all fluorine atoms, and the shape and signal was almost equal to that of the starting ligand, which suggests that fluorine atoms do not seem to be affected by being grafted on the NP surface. The chemical shift (δ) of the fluorine on the NPs is slightly shifted when compared to free ligand (-71.34 ppm vs. -71.48 ppm, respectively), using TFA as the reference. Supplementary Figure 17 shows the comparison between ^{19}F-NMR spectra of all NPs and the free ligand HS-PEG-F.

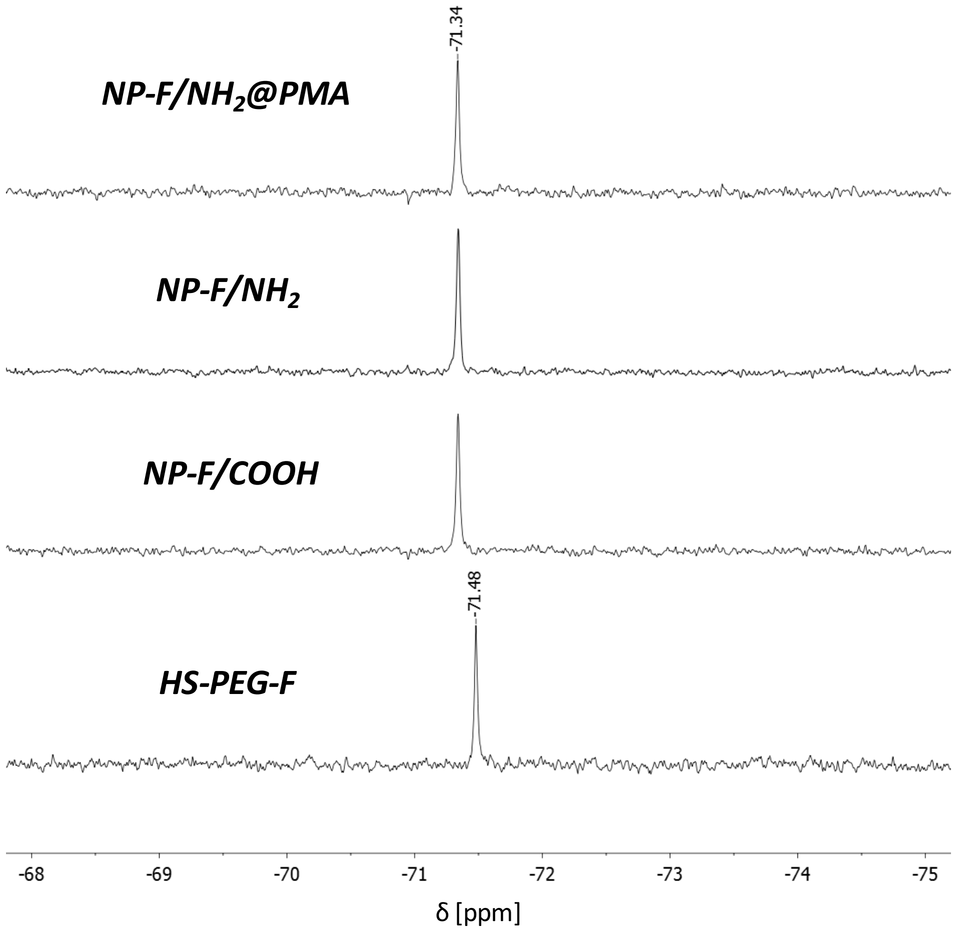

Supplementary Figure 17: ^{19}F-NMR spectra comparison between NP-F/COOH, NP-F/NH_2 and NP-F/NH_2@PMA and ligand HS-PEG-F, recorded in water using TFA as the chemical shift reference.

Elemental Analysis (EA) measurement

To further confirm the ratio of ligands on the NPs, samples of NP-F/COOH and NP-F/NH_2 were submitted to EA in the microanalysis service of the Complutense University of Madrid (Madrid, Spain). By this kind of analysis we obtained the percentage in mass of carbon, hydrogen, and sulfur present in the sample. Since the nitrogen amount was below the quantification limit of the detection method (< 0.5 %) its measurement was not taken into account. The so-obtained results for each sample were fitted as close as possible to the measured sample composition through an iterative process by using the Analysis tool in ChemBioDraw Ultra Software, with the following premises: the amount of sulfur was used to determine the number of thiolated PEG ligands; the parts of the ligands exclusive to each ligand were used to determine the ratio between them; the carbon and hydrogen values were finally adjusted with the addition of ethylene glycol units taking into account that each PEG ligand has an approximate number of ethylene glycol units of 66 ± 9, and the amount of gold atoms per NP was indirectly estimated from the ICP-MS and TEM data to be roughly 800 atoms for NP-F/COOH and 1200 atoms for NP-F/NH_2. Taking these data into account the EA data fitted with a 75/25 ratio of HS-PEG-F with

either HS-PEG-COOH or HS-PEG-NH_2, as we had already observed by ^{1}H-NMR (Supplementary Figure 16).

NP-F/COOH: calculated for $C_{22940}H_{44720}Au_{800}F_{1080}N_{160}O_{11150}S_{160}$ is C: 40.25 %; H: 6.59 %; S: 0.75 %; measured is C: 40.42 ± 0.35 %; H: 6.49 ± 0.30 %; S: 0.76 ± 0.35 %.

NP-F/NH₂: calculated for $C_{22566}H_{43954}Au_{1200}F_{1224}N_{228}O_{10874}S_{182}$ is C: 35.76 %; H: 5.85 %; S: 0.77 %; measured is C: 35.50 ± 0.35 %; H: 5.73 ± 0.30 %; S: 0.77 ± 0.35 %.

Supplementary Table 1: Summary of EA results for each NP.

NP type	Estimated formula	EA	C [%])	H [%]	S [%]
NP-F/COOH	$C_{22940}H_{44720}Au_{800}F_{1080}N_{160}O_{11150}S_{160}$	calculated	40.25	6.59	0.75
		measured	40.42 ± 0.35	6.49 ± 0.30	0.76 ± 0.35
NP-F/NH_2	$C_{22566}H_{43954}Au_{1200}F_{1224}N_{228}O_{10874}S_{182}$	calculated	35.76	5.85	0.77
		measured	35.50 ± 0.35	5.73 ± 0.30	0.77 ± 0.35

Transverse relaxation time (T_2) measurement of fluorine

T_2 values for fluorine in the unbound ligand HS-PEG-F and when bound to NPs in NP-F/COOH, NP-F/NH_2, and NP-F/NH_2@PMA were measured twice for each sample in a mixture of water and D_2O. ^{19}F T_2 measurements were performed using Bruker's standard CPMG sequence at 470.55 MHz with the following parameters: TR = 6 s, 16 echos covering a range between 0.002 and 2.5 s, SW = 20 ppm, NS = 16, and 32K points. T_2 values for each sample were calculated by fitting the NMR signal intensity (I) decay to a mono-exponential decay equation (Supplementary Equation 2, GraphPad), with the scaling factor A:

$$I = A \cdot e^{-\tau/T_2} \qquad \text{(Supplementary Equation 2)}$$

where I is directly measured on the NMR spectra, τ is automatically obtained in MestReNova from the experiment parameters and the T_2 value is obtained from the fitting. In our case, we observed very high values for the free ligand and NPs without PMA, as expected. For the NPs with PMA linked on the surface we observed a slight decrease in T_2 value with respect to NPs without PMA, which is expected considering that the PMA coating may partially entrap some of the fluorine atoms leading to restricted mobility, although the T_2 value obtained for those NPs is still very high (Supplementary Figure 18).

a

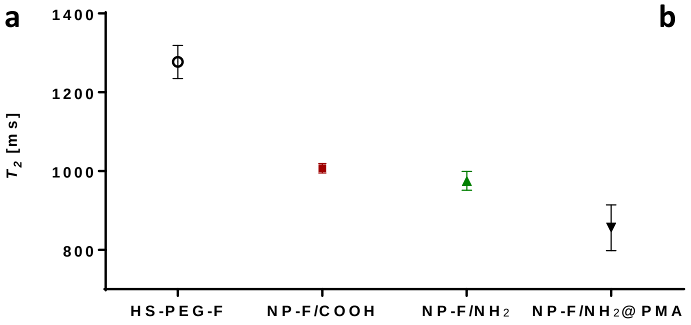

b

Sample	T_2 [ms]
HS-PEG-F	1277 ± 42
NP-F/COOH	1007 ± 12
NP-F/NH_2	975 ± 24
NP-F/NH_2@PMA	856 ± 58

Supplementary Figure 18: **a** Graphical representation of T_2 values for the studied samples. **b** Table with the average T_2 values for each sample with the standard deviation (SD) corresponding to 2 measurements.

Protein covalent binding (HSA and aTR)

For the protein binding studies, 3 different proteins were used, namely Albumin from human serum (HSA), apo-Transferrin human (aTR) and Transferrin human (TR). For covalent binding studies HSA and aTR were used and for natural adsorption studes HSA and TR were employed, as it will be described in the following section.

All reagents were purchased and directly used without further purification. The proteins were purchased from Sigma Aldrich: Albumin from human serum (HSA, #A3782) and apo-Transferrin human (aTR, #T2036). N-(3-Dimethylaminopropyl)-N'-ethylcarbodiimide hydrochloride (EDC, #22980) and N-Hydroxysulfosuccinimide sodium salt (Sulfo-NHS, #24510) were obtained from Thermo Scientific. Incubations at specific temperatures and stirring were carried out in a VWR Incubating Mini Shaker.

Covalent binding protocol

The covalent binding of proteins to NP-F/COOH was carried out by means of EDC chemistry, which allows for the formation of amide bonds between the amino functions of the protein molecules (HSA or aTR) and the carboxylic groups of PMA at the NP surface [9]. An excess of protein molecules was added to each NP (300 HSA or aTR molecules added per NP), and the amount of proteins bound to the NPs was controlled by using different quantities of EDC, *cf.* Supplementary Table 2. In detail, NPs were initially diluted in MES buffer (100 mM, pH 6.5) at a concentration of 0.6 µM, and mixed with EDC/Sulfo-NHS solutions freshly prepared before their use. Sulfo-NHS was used together with the EDC (2.4 eq of Sulfo-NHS per eq of EDC), in order to increase the efficiency of the coupling. The mixture was incubated at 37 °C for 25 min under gentle shaking for the activation of the carboxylic groups. Then, the excess of EDC/Sulfo-NHS was removed by centrifugation (820 g, 8 min) with Amicon Ultra centrifugal filters (Millipore, 100 kDa MWCO), and the activated NPs were immediately mixed with the required amounts of protein solution in HEPES buffer (4-(2-hydroxyethyl)-1-piperazineethanesulfonic acid, 100 mM, pH 8.5). The reaction was left overnight at 25 °C with gentle shaking. Next day, the NP samples were washed with HEPES buffer (100 mM, pH 8.5) by centrifugation (820 g, 8 min) with Amicon Ultra centrifugal filters (100 kDa MWCO), whereby the NPs were retained in the upper part of the filter and excess proteins were eluted. This step was repeated 3 times using 5 mL of buffer solution in each washing step. Elutes were checked with Bradford solution that there was no more protein in the last filtrate. Finally, the samples were redispersed in 400 µL of HEPES buffer (10 mM, pH 8.5), and UV/Vis absorption measurements allowed for fixing all the samples to the same concentration (c_{NP} = 27 nM).

Supplementary Table 2: NP samples prepared with variable amounts of attached protein molecules (HSA or aTR) per NP. The number of protein added per NP was fixed to 300 in each sample, whereas the EDC to NP ratio (EDC/NP) was varied as shown.

HSA			aTR		
**Sample**	**EDC/NP**	**HSA/NP**	**Sample**	**EDC/NP**	**aTR/NP**
NP_HSA_0_cov	0	300	NP_aTR_0_cov	0	300
NP_HSA_1_cov	720	300	NP_aTR_1_cov	700	300
NP_HSA_2_cov	1600	300	NP_aTR_2_cov	1400	300
NP_HSA_3_cov	3300	300	NP_aTR_3_cov	2700	300
NP_HSA_4_cov	6200	300	NP_aTR_4_cov	4200	300
NP_HSA_5_cov	9500	300	NP_aTR_5_cov	18000	300
NP_HSA_6_cov	12000	300	NP_aTR_6_cov	140000	300
NP_HSA_7_cov	15000	300			
NP_HSA_8_cov	16000	300			
NP_HSA_9_cov	33000	300			
NP_HSA_10_cov	57000	300			
NP_HSA_11_cov	160000	300			

Bradford assay

All reagents were purchased and directly used without further purification. Bradford reagent was obtained from AppliChem (Germany). The absorbance measurements were performed in a 96-well plate with a TECAN Genios Pro 96/384 multifunction microplate reader.

The amount of protein bound to the NPs was quantified by the Bradford assay [10]. The samples were diluted up to a final concentration of c_{NP} = 27 nM, so that the analytical signal from the NP sample with the highest amount of linked protein fell into the concentration range of the calibration curve. Even if the absorbance of the NPs is negligible at such concentration level, and therefore there is no contribution to the measurement signal (*i.e.* absorbance at 595 nm), the calibration curve was carried out using the naked NPs (*i.e.* NPs without protein linked) at 27 nM in HEPES buffer (10 mM, pH 8.5) as matrix solution. In this way, the standards were prepared by adding increasing concentrations of protein (0, 5, 10, 15, 20 and 25 µg/mL) to this matrix solution. For the HSA, the standards used were 0, 5, 10, 15, 20, and 25 µg/mL, whereas for the aTR determinations the standards were 0, 1.25, 2.5, 5, 10, and 15 µg/mL.

The protocol used for the assay was the following: 250 µL of standards or NP samples were added into the wells of a 96-well plate. Then, 250 µL of Bradford reagent solution was added to each well, and after 2 min the absorbance at 595 was recorded. The analytical signal (A_{595}) was calculated as the absorbance value of the standard or sample minus the absorbance of the zero standard. All the measurements were carried out in triplicate with the HSA and duplicate with the aTR. The obtained calibration curves with the two studied proteins, HSA and aTR, are presented in Supplementary Figure 19. The interpolation of the signals of the samples from the calibration curve gave the concentrations of protein in µg/mL, which was then converted to mol of protein per mol of NP (HSA/NP or aTR/NP), *cf* Supplementary Table 3 and Supplementary Figure 20.

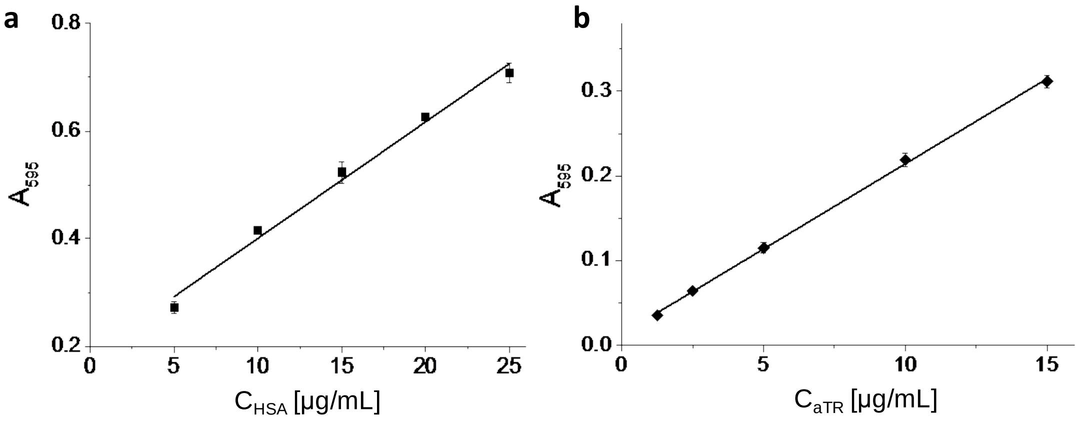

Supplementary Figure 19: Calibration curves obtained for **a** HSA and **b** aTR. Error bars represent the standard deviation (SD) of three replicates. Measurements were performed in 1 cm pathlength cuvettes.

Supplementary Table 3: Quantification of protein content in the NP samples (c_{NP} = 27 nM) prepared with variable amounts of attached protein molecules (HSA or aTR) per NP. Protein content is expressed in µg/mL (C_{HSA} or C_{aTR}), and the HSA/NP or aTR/NP molar ratio is also calculated by using the molecular weights of the proteins (MW_{HSA}=66 kDa and MW_{aTR}=78 kDa). Values are the mean ± the standard deviation (SD) of two or three replicates.

HSA			aTR		
**Sample**	**C_{HSA} [µg/mL]**	**HSA/NP**	**Sample**	**C_{aTR} [µg/mL]**	**aTR/NP**
NP_HSA_1_cov	2.20 ± 0.24	1.24 ± 0.13	NP_aTR_1_cov	1.45 ± 0.06	0.69 ± 0.03
NP_HSA_2_cov	2.64 ± 0.12	1.48 ± 0.07	NP_aTR_2_cov	1.59 ± 0.03	0.76 ± 0.01
NP_HSA_3_cov	3.57 ± 0.51	2.00 ± 0.28	NP_aTR_3_cov	2.44 ± 0.13	1.16 ± 0.06
NP_HSA_4_cov	4.07 ± 0.41	2.28 ± 0.23	NP_aTR_4_cov	3.24 ± 0.07	1.54 ± 0.03
NP_HSA_5_cov	5.82 ± 0.36	3.27 ± 0.20	NP_aTR_5_cov	3.65 ± 0.07	1.73 ± 0.03
NP_HSA_6_cov	7.54 ± 0.38	4.23 ± 0.22	NP_aTR_6_cov	3.71 ± 0.02	1.76 ± 0.01
NP_HSA_7_cov	8.00 ± 0.36	4.49 ± 0.20			
NP_HSA_8_cov	8.03 ± 0.34	4.51 ± 0.19			
NP_HSA_9_cov	9.29 ± 0.31	5.21± 0.17			
NP_HSA_10_cov	8.97 ± 0.07	5.03 ± 0.04			
NP_HSA_11_cov	7.73 ± 0.19	4.34 ± 0.11			

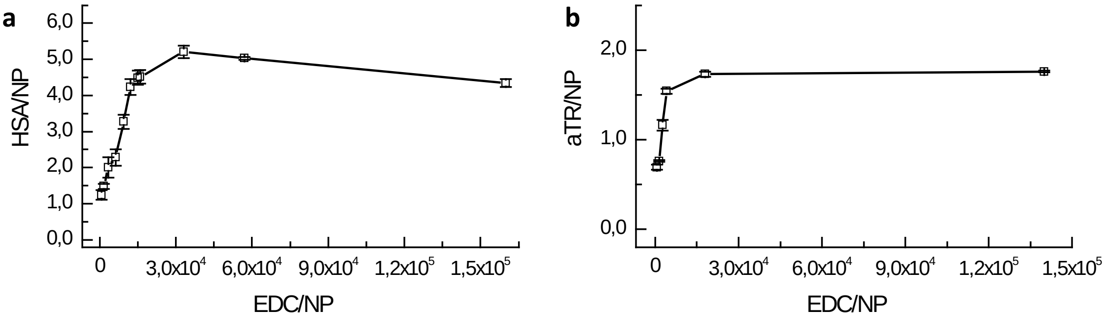

Supplementary Figure 20: Plot of number $N_{P/NP}$ of bound proteins per NP (*i.e.*, HSA/NP or aTR/NP) versus the number $N_{EDC/NP}$ of added EDC molecules per NP (*i.e.*, EDC/NP) for the different samples with **a** HSA and **b** aTR. Error bars represent the standard deviation (SD) of the replicates.

Gel Electrophoresis

The different NP-protein complexes listed in Supplementary Table 3 were concentrated with Amicon Ultra centrifugal filters (molecular weight cut-off (MWCO) 100 kDa), and run in a 2% agarose gel at 5 V·cm^{-1} for 30 min in Tris-Borate-EDTA buffer (TBE 0.5x), aiming to see differences in their electrophoretic mobilities which could be related to different protein binding, *cf.* Supplementary Figure 21 [11].

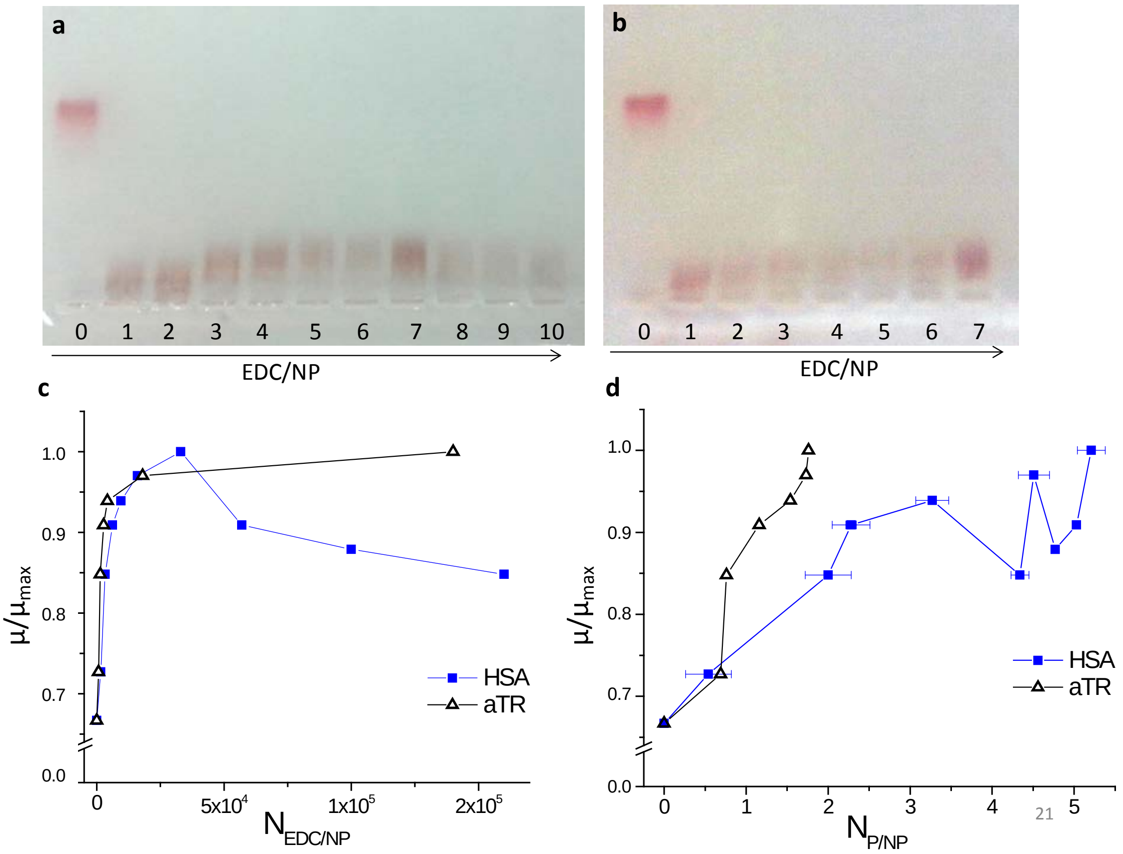

Supplementary Figure 21: Gel electrophoresis of NPs with different proteins attached. The bands of the NP-protein complexes are visible on the photo of the gel. Due to their negative charge the NP-protein complexes migrated from the wells (on the bottom of the photo) towards the plus pole (on the top of the photo). **a** Samples prepared by covalent binding of HSA to NP-F/COOH with increasing amounts of EDC per NP as follows: 1=0, 2=1600, 3=3300, 4=6200, 5=9500, 6=16000, 7=33000, 8=57000, 9=100000, 10=160000. **b** Samples prepared by covalent binding of aTR to NP-F/COOH with increasing amounts of EDC per NP as follows: 1=0, 2=700, 3=1400, 4=2700, 5=4200, 6=18000, 7=140000. As control sample (0), phosphine-Au NPs with 10 nm core diameter were used [11]. **c** Plot of the EDC amount per NP ($N_{EDC/NP}$) versus the relative mobility of the NP samples μ/μ_{max} calculated as the distance l each NP band had migrated,

normalized by the distance l_{max} of the NP band that had migrated fastest: $\mu/\mu_{max} = l/l_{max}$ [11]. **d** Plot of the protein (HSA or aTR) amount per NP ($N_{P/NP}$) versus the relative mobility of the NP samples μ/μ_{max} calculated as the distance l each NP band had migrated, normalized by the distance l_{max} of the NP band that had migrated fastest: $\mu/\mu_{max} = l/l_{max}$

Protein natural adsorption (HSA or TR)

HSA or Transferrin (TR, sigma #T8158) dissolved in PBS was mixed at r.t. with known amounts of NP-F/NH_2@PMA also dissolved in PBS in a total volume of 465 µL, so that the final HSA or TR concentration c_{HSA} or c_{TR} ranged from 0 to 190 µM or to 200 µM, respectively, and NP-F/NH_2@PMA concentration ranged from 0.5-1 µM. The final protein concentration and the protein/NP molar ratio $N_{HSA/NP(added)}$ or $N_{TR/NP(added)}$ in each sample are shown in Supplementary Table 4. The so-obtained mixtures were introduced in NMR tubes and measured as it will be described in the following section.

Supplementary Table 4: Samples of NP-F/NH_2@PMA with increasing amount of HSA or TR protein.

Sample	c_{HSA} [µM]	$N_{HSA/NP(added)}$	Sample	c_{TR} [µM]	$N_{TR/NP(added)}$
NP_HSA_0_ads	0	0	NP_TR_0_ads	0	0
NP_HSA_1_ads	0.2	0.3	NP_TR_1_ads	0.1	0.2
NP_HSA_2_ads	1	2	NP_TR_2_ads	0.4	0.7
NP_HSA_3_ads	6	11	NP_TR_3_ads	1	2
NP_HSA_4_ads	10	19	NP_TR_4_ads	4	7
NP_HSA_5_ads	20	29	NP_TR_5_ads	10	17
NP_HSA_6_ads	52	58	NP_TR_6_ads	40	67
NP_HSA_7_ads	190	185	NP_TR_7_ads	200	333

^{19}F-NMR diffusion constant measurement protocol

All NMR data were collected on a Bruker AVANCE III NMR spectrometer (11.7 T, 470.59 MHz for ^{19}F) equipped with a 5 mm ^{1}H/^{19}F BBI probe with actively shielded z-gradient that was used in combination with a Bruker gradient amplifier providing a maximum current of 10 A, which results in a 65 G/cm gradient. ^{19}F diffusion NMR measurements were performed using stimulated echo with bipolar gradient pulses from Bruker's sequence library (stebpgp1s) with the following parameters: 4k acquisition points, SW 15 ppm, NS ≥ 480, DS 32, D1 2 s, D20 (gradient length) 0.5 s, P30 (diffusion delay/2) 1.5 ms, and 12 equally spaced gradient strengths from 5 to 95%. NMR measurements were performed using deuterium lock and while the sample was spinning. Contradictory indications are given in the literature regarding the spinning or not of the samples during DOSY experiments[12]. However, we performed control experiments with and without rotation and we did not observe significant differences in the diffusion constants obtained. Deuterium oxide (D_2O) used was purchased from euriso-top (#D215F) and TFA used as reference for ^{19}F-NMR was purchased from Riedel de Haën (#61030). All measurements were performed at 25 °C. Diffusion spectra were analyzed using Mnova 11.0 software (http://mestrelab.com/resources/data-analysis/).

As before, TFA was used as a chemical shift reference (δ = -76.55 ppm). All diffusion experiments were performed in 5 mm standard NMR tubes with a coaxial insert carrying TFA in D_2O (0.024 % v/v). Such coaxial insert was introduced inside the NMR tube together with the sample with the idea to diminish as much as possible the modification and manipulation of the sample by adding TFA or D_2O, although this leads to a lower signal to noise ratio, since the insert occupies almost half of the space dedicated for the sample. All samples had at least a total volume of 465 µL, which was the minimum amount observed by us to ensure a reliable measurement in this kind of system. All NMR tubes, coaxial inserts, and NMR Pasteur pipettes were rinsed 3 times with the corresponding buffer used for each sample (HEPES or PBS) before sample loading. For the measurements of natural adsorption of HSA, the NMR tubes, inserts, and pipettes were passivated with a solution of HSA (10 mg/mL) for 15-30 min and then rinsed 3 times with PBS to prevent protein adsorption on the glass, which would then modify the HSA concentration in the sample.

Data analysis. Diffusion constant calculation

Diffusion constants for each sample were calculated by fitting the NMR signal intensity (*I*) decay to a mono-exponential decay equation (Supplementary Equation 3, GraphPad), with the scaling factor A:

$$I = A \cdot e^{-D \cdot Z} \quad \text{(Supplementary Equation 3)}$$

where *I* is directly measured on the NMR spectra, Z corresponds to the Z values, i.e. the gradient strengths scaled according to the Tanner-Stejskal model (Supplementary Equation 4) and the diffusion constant (*D*) is obtained from the fitting (Supplementary Figure 22).

$$Z = (\gamma \delta G)^2 (\Delta - \delta/3) \quad \text{(Supplementary Equation 4)}$$

Where γ [Hz/T] is the gyromagnetic ratio of ^{19}F, δ [s] is gradient length, Δ [s] is diffusion delay and G [T/m] is the gradient strength[13,14].

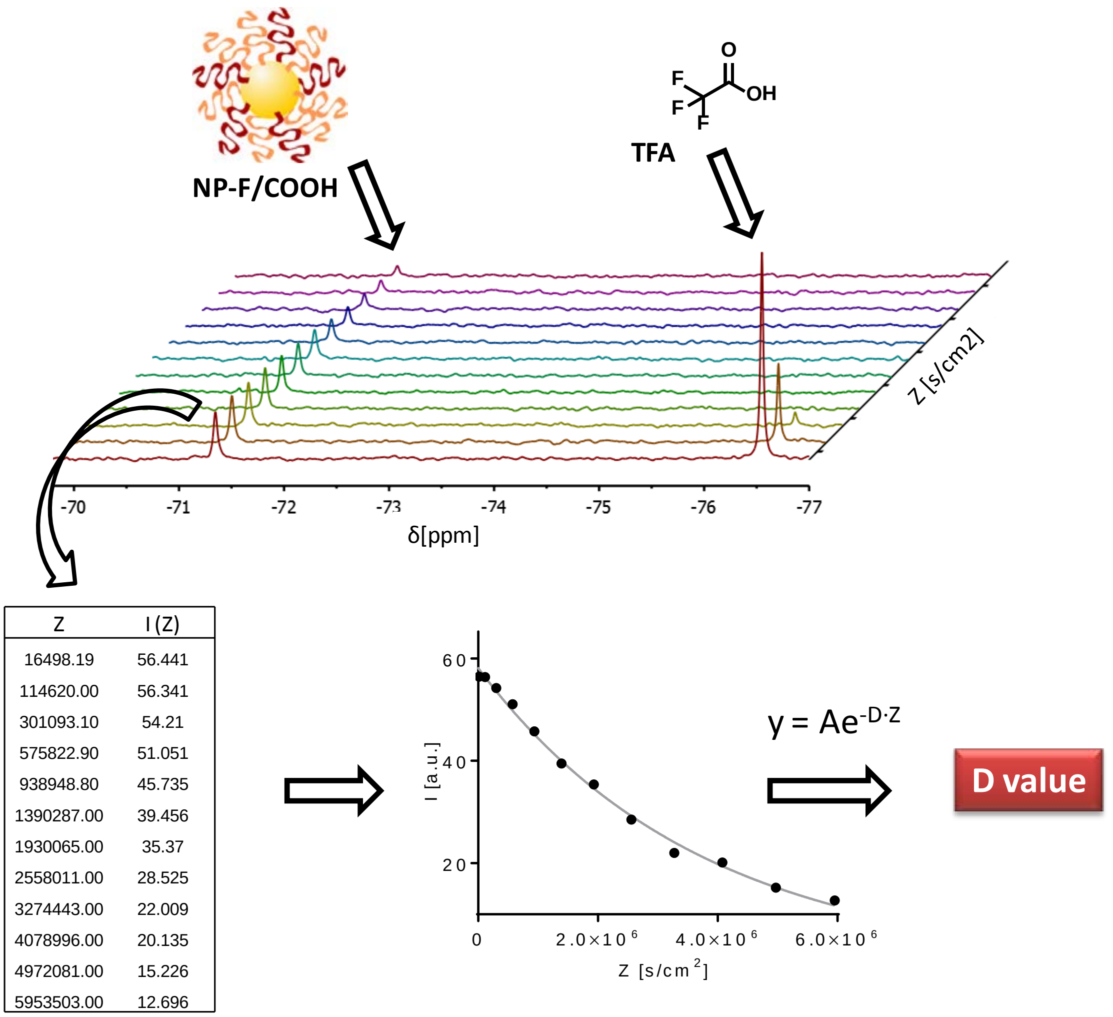

Z	I (Z)
16498.19	56.441
114620.00	56.341
301093.10	54.21
575822.90	51.051
938948.80	45.735
1390287.00	39.456
1930065.00	35.37
2558011.00	28.525
3274443.00	22.009
4078996.00	20.135
4972081.00	15.226
5953503.00	12.696

Supplementary Figure 22: Work-flow to obtain the diffusion constant *D* from ^{19}F diffusion NMR experiment, which shows the raw data extraction and exponential decay fitting to retrieve a value for the diffusion constant *D*.

Covalent binding of HSA and aTR

NMR samples of NP-F/COOH covalently linked to HSA or aTR were prepared as described before. Hence, 465 µL of a protein functionalized NP solution in HEPES of at least 0.7 µM in NPs were introduced in NMR tubes as described before, and the ^{19}F diffusion NMR spectra were recorded. The work-flow shown in Supplementary Figure 22 was followed for each single measurement, and the mean *D* values for each EDC/NP ratio together with the corresponding standard deviations (SD) are shown in Supplementary Table 5 for HSA-modified NPs and Supplementary Table 6 for aTR-modified NPs. The so-obtained fitted decay curves are shown in Supplementary Figure 23 for HSA, and Supplementary Figure 24 for aTR.

Supplementary Table 5: Diffusion constants *D* (± SD) as experimentally obtained from diffusion spectra analysis by fitting the NMR signal intensity to an exponential decay for NP-F/COOH modified with HSA. The *n* value refers to the number of independent measurements performed for each EDC/NP ratio.

Sample	$N_{EDC/NP}$	D [10^{-11} m^2/s]	*n*
NP_HSA_0_cov	0	2.59 ± 0.04	6
NP_HSA_1_cov	720	2.60 ± 0.03	2
NP_HSA_2_cov	1600	2.57 ± 0.07	2
NP_HSA_3_cov	3300	2.48 ± 0.09	2
NP_HSA_4_cov	6200	2.46 ± 0.001	2
NP_HSA_5_cov	9500	2.35 ± 0.06	2
NP_HSA_6_cov	12000	2.26	1
NP_HSA_7_cov	15000	2.14	1
NP_HSA_8_cov	16000	2.12 ± 0.09	3
NP_HSA_9_cov	33000	2.17 ± 0.10	2
NP_HSA_10_cov	57000	2.16 ± 0.09	2
NP_HSA_11_cov	160000	2.13 ± 0.10	3

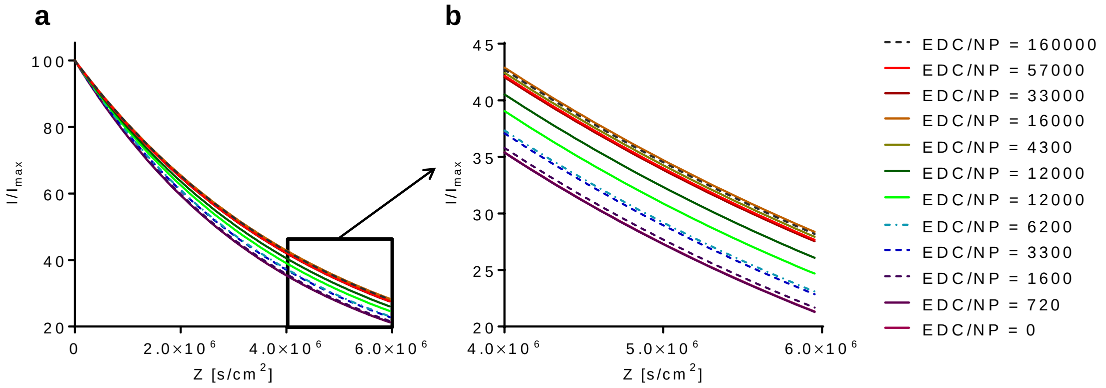

Supplementary Figure 23: **a** Plot of normalized fitted mean mono exponential decay curves for each EDC/NP ratio for NP-F/COOH modified with HSA. **b** Zoomed in area of the same plot as in **a** to better observe the curves.

Supplementary Table 6: Diffusion constants *D* as experimentally obtained from diffusion spectra analysis by fitting the NMR signal intensity to an exponential decay for NP-F/COOH modified with aTR. The *n* value refers to the number of independent measurements performed for each EDC/NP ratio.

Sample	$N_{EDC/NP}$	D [10^{-11} m^2/s]	*n*
NP_aTR_0_cov	0	2.59 ± 0.04	6
NP_aTR _1_cov	700	2.57 ± 0.05	2
NP_aTR _2_cov	1400	2.36 ± 0.03	2
NP_aTR _3_cov	2700	2.19 ± 0.12	2
NP_aTR _4_cov	4200	2.14 ± 0.01	2
NP_aTR _5_cov	18000	2.07 ± 0.14	2
NP_aTR _6_cov	140000	2.11 ± 0.19	2

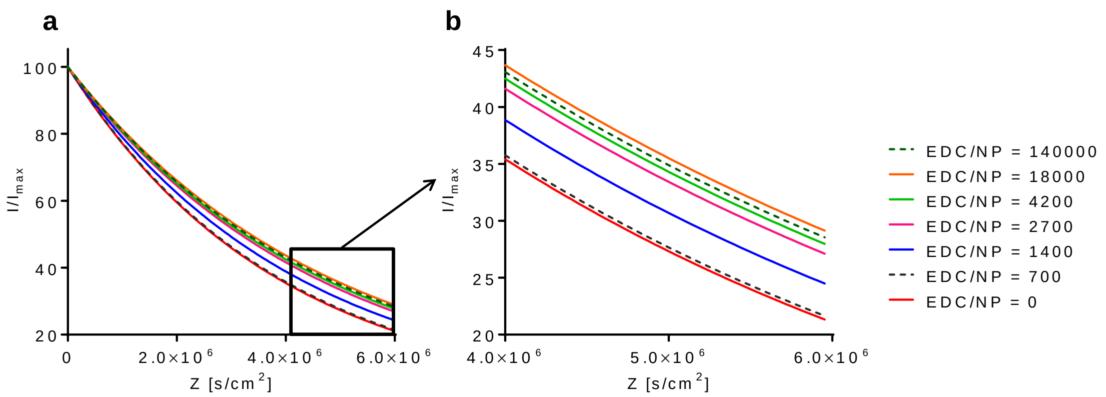

Supplementary Figure 24: **a** Plot of normalized fitted mean mono exponential decay curves for each EDC/NP ratio for NP-F/COOH modified with aTR. **b** Zoomed in area of the same plot as in **a** to better observe the curves.

Natural adsorption of HSA or TR

NMR samples of NP-F/NH_2@PMA mixed with increasing concentrations of HSA or TR in PBS were prepared as described before. Hence, 465 µL of a PBS solution of a NP and protein mixture of at least 0.5 µM in NPs and with HSA concentrations ranging from 0 to 0.19 mM or TR concentrations from 0 to 0.2 mM were introduced in NMR tubes as described before, and the ^{19}F diffusion NMR spectra were recorded. The work-flow shown in Supplementary Figure 22 was followed for each single measurement, and the mean *D* values for each protein concentration together with the corresponding standard deviations (SD) are shown in Supplementary Table 7 and 8. The so-obtained fitted decay curves are shown in Supplementary Figure 25 and 26.

Supplementary Table 7: Diffusion constants *D* as experimentally obtained from diffusion spectra analysis by fitting the NMR signal intensity to an exponential decay for NP-F/NH_2@PMA mixed with HSA. The *n* value refers to the number of independent measurements performed for each HSA concentration.

Sample	c_{HSA} [mM]	*D* [10^{-11} m^2/s]	*n*
NP_HSA_0_ads	0	3.29 ± 0.30	3
NP_HSA_1_ads	0.0002	3.25 ± 0.03	2
NP_HSA_2_ads	0.001	3.08 ± 0.40	2
NP_HSA_3_ads	0.006	2.81 ± 0.10	3
NP_HSA_4_ads	0.01	2.53 ± 0.10	2
NP_HSA_5_ads	0.02	2.16 ± 0.20	3
NP_HSA_6_ads	0.052	1.96 ± 0.06	3
NP_HSA_7_ads	0.19	1.87 ± 0.09	3

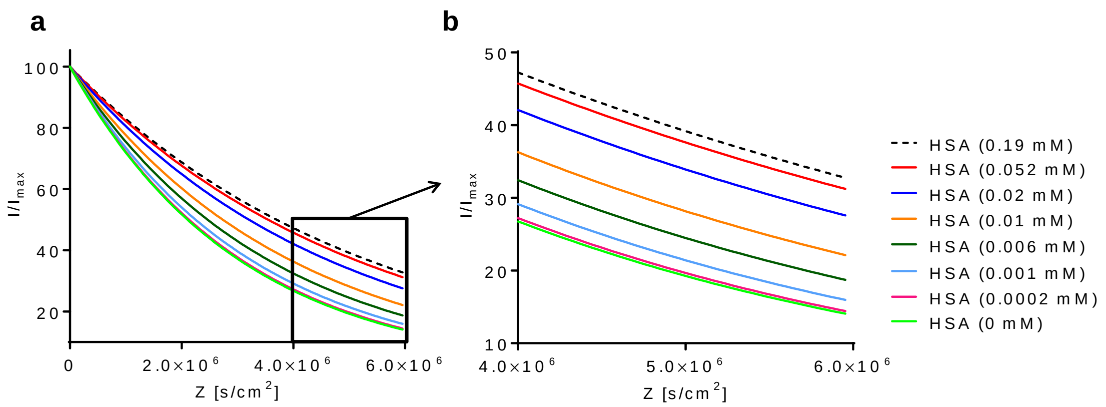

Supplementary Figure 25: **a** Plot of normalized fitted mean mono exponential decay curves for increasing concentration of HSA in the presence of NP-F/NH_2@PMA. **b** Zoomed in area of the same plot as in **a** to better observe the curves.

Supplementary Table 8: Diffusion constants *D* as experimentally obtained from diffusion spectra analysis by fitting the NMR signal intensity to an exponential decay for NP-F/NH_2@PMA mixed with TR. The *n* value refers to the number of independent measurements performed for each TR concentration.

Sample	c_{TR} [mM]	*D* [10^{-11} m^2/s]	*n*
NP_TR_0_ads	0	2.69 ± 0.05	3
NP_TR_1_ads	0.0001	2.64 ± 0.14	3
NP_TR_2_ads	0.0004	2.31 ± 0.09	2
NP_TR_3_ads	0.001	2.09 ± 0.12	3
NP_TR_4_ads	0.004	1.68 ± 0.16	5
NP_TR_5_ads	0.01	1.41 ± 0.05	6
NP_TR_6_ads	0.04	1.34 ± 0.08	6
NP_TR_7_ads	0.2	1.26 ± 0.03	5

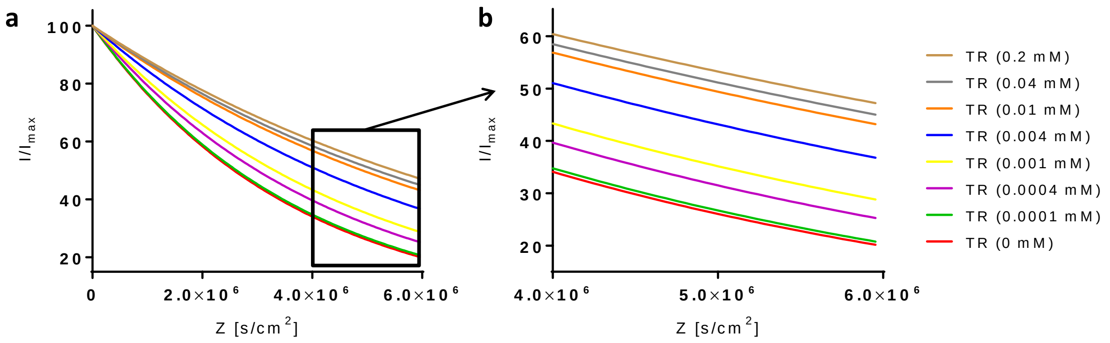

Supplementary Figure 26: **a** Plot of normalized fitted mean mono exponential decay curves for increasing concentration of TR in the presence of NP-F/NH_2@PMA. **b** Zoomed in area of the same plot as in **a** to better observe the curves.

Size calculation by ^{19}F diffusion NMR measurements

The hydrodynamic radii r_h for each NP sample was calculated using the diffusion constant D as calculated from the diffusion measurements and applying the Einstein-Stokes relation, assuming spherical shape for NPs (Supplementary Equation 5).

$$r_h = k_B T/(6\pi\eta D) \qquad \text{Supplementary Equation 5}$$

Where, r_h is the hydrodynamic radius, η is the dynamic viscosity, T is the absolute temperature in Kelvin, D is the diffusion constant, and k_B is the Boltzmann constant.

Calculation of r_h for NP-F/COOH covalently linked to HSA and aTR

r_h was calculated for the case of NP-F/COOH conjugated either with HSA or aTR by introducing the previously calculated diffusion constants D for each sample in the Einstein-Stokes relation (Supplementary Table 9 and 10) and by using the following parameters:

T = 298 K

$k_B = 1.38065\cdot10^{-23}$ $m^2kg\cdot s^{-2}K^{-1}$

$\eta_{H2O} = 0.000891$ $kg\cdot m^{-1}s^{-1}$

Supplementary Table 9: Hydrodynamic radii r_h obtained from experimentally measured diffusion constants D for NPs covalently modified with HSA and the corresponding standard deviation (SD). The n value refers to the number of independent measurements performed for each EDC/NP ratio.

Sample	$N_{EDC/NP}$	r_h [nm]	n
NP_HSA_0_cov	0	9.45 ± 0.15	6
NP_HSA_1_cov	720	9.44 ± 0.13	2
NP_HSA_2_cov	1600	9.55 ± 0.25	2
NP_HSA_3_cov	3300	9.89 ± 0.37	2
NP_HSA_4_cov	6200	9.96 ± 0.01	2
NP_HSA_5_cov	9500	10.43 ± 0.28	2
NP_HSA_6_cov	12000	10.86	1
NP_HSA_7_cov	15000	11.44	1
NP_HSA_8_cov	16000	11.60 ± 0.48	3
NP_HSA_9_cov	33000	11.35 ± 0.70	2
NP_HSA_10_cov	57000	11.38 ± 0.50	2
NP_HSA_11_cov	160000	11.56 ± 0.81	3

Supplementary Table 10: Hydrodynamic radii r_h as obtained from the experimentally measured diffusion constants D for NPs covalently modified with aTR, together with the corresponding standard deviation (SD). The n value refers to the number of independent measurements performed for each EDC/NP ratio.

Sample	$N_{EDC/NP}$	r_h [nm]	*n*
NP_aTR_0_cov	0	9.45 ± 0.15	6
NP_aTR _1_cov	700	9.54 ± 0.17	2
NP_aTR _2_cov	1400	10.38 ± 0.15	2
NP_aTR _3_cov	2700	11.20 ± 0.61	2
NP_aTR _4_cov	4200	11.75 ± 0.36	2
NP_aTR _5_cov	18000	11.87 ± 0.83	2
NP_aTR _6_cov	140000	11.66 ± 0.66	2

Calculation of r_h for HSA or TR naturally adsorbed onto NP-F/NH_2@PMA

r_h was calculated for the case of NP-F/NH_2@PMA mixed with increasing concentrations of HSA, by introducing the value for the diffusions constants *D* as previously calculated for each sample (*cf*. Supplementary Table 7) in the Einstein-Stokes relation (*cf*. Supplementary Equation 5). The viscosity of the HSA-water mixture $\eta_{HSA/H2O}$ was calculated from the viscosity of water η_{H2O} and the additional increase in viscosity due to the presence of HSA. The following parameters were used for the calculations (Supplementary Table 11):

T = 298 K

k_B = 1.38065 x 10^{-23} $m^2kg{\cdot}s^{-2}K^{-1}$

$\eta_{HSA/H2O} = \eta_{H2O}$ x (1 + c_{HSA} x η_{HSA}) ; η_{HSA} = 4.2 cm^3g^{-1} = viscosity of HSA

Supplementary Table 11: Hydrodynamic radii r_h obtained from the experimentally measured diffusion constants *D* for NPs mixed with increasing amounts of HSA, and the corresponding standard deviation (SD). The *n* value refers to the number of independent measurements performed for each HSA concentration.

Sample	c_{HSA} [mM]	r_h [nm]	*n*
NP_HSA_0_ads	0	7.50 ± 0.82	3
NP_HSA_1_ads	0.0002	7.54 ± 0.06	2
NP_HSA_2_ads	0.001	7.84 ± 0.30	2
NP_HSA_3_ads	0.006	8.71 ± 0.36	3
NP_HSA_4_ads	0.01	9.66 ± 0.39	2
NP_HSA_5_ads	0.02	11.54 ± 0.78	3
NP_HSA_6_ads	0.052	12.36 ± 0.35	3
NP_HSA_7_ads	0.19	12.44 ± 0.58	3

The same procedure was followed for calculating the size of NP-F/NH_2@PMA mixed with increasing concentrations of TR. The viscosity of the TR-water mixture $\eta_{TR/H2O}$ was calculated from the viscosity of water η_{H2O} and the additional increase in viscosity due to the presence of TR. The following parameters were used for the calculations (Supplementary Table 12):

T = 298 K

k_B = 1.38065 x 10^{-23} $m^2kg{\cdot}s^{-2}K^{-1}$

$\eta_{TR/H2O} = \eta_{H2O}$ x (1 + c_{TR} x η_{TR}) ; η_{TR} = 4.4 cm^3g^{-1} = viscosity of TR

Supplementary Table 12: Hydrodynamic radii r_h obtained from the experimentally measured diffusion constants D for NPs mixed with increasing amounts of TR, and the corresponding standard deviation (SD). The n value refers to the number of independent measurements performed for each TR concentration.

Sample	c_{TR} [mM]	r_h [nm]	n
NP_TR_0_ads	0	9.10 ± 0.17	3
NP_TR_1_ads	0.0001	9.32 ± 0.48	3
NP_TR_2_ads	0.0004	10.61 ± 0.42	2
NP_TR_3_ads	0.001	11.76 ± 0.64	3
NP_TR_4_ads	0.004	14.72 ± 1.55	5
NP_TR_5_ads	0.01	17.33 ± 0.61	6
NP_TR_6_ads	0.04	18.07 ± 1.03	6
NP_TR_7_ads	0.2	18.18 ± 0.49	5

Size correlation for NP-F/COOH covalently bound to either HSA or aTR

The mean radii obtained by diffusion measurements for the samples of HSA (*cf.* Supplementary Table 9) or aTR (*cf.* Supplementary Table 10) covalent binding to NP-F/COOH, display sigmoidal distributions, which were fitted with a logistic function (OriginLab), *cf.* fitting parameters in Supplementary Table 13, as follows:

$$r_h(N_{EDC/NP}) = r_{h,max} + \frac{r_{h,max} - r_{h,min}}{1 + \left(\frac{N_{EDC/NP\,(50)}}{N_{EDC/NP}}\right)^p}$$ Supplementary Equation 6

Hereby, r_h is the mean hydrodynamic radius obtained by diffusion measurements, $r_{h,max}$ and $r_{h,min}$ are the highest (*i.e.*, saturation) and lowest radii (*i.e.*, without EDC added, $r_{h,min} = r_h(0)$), respectively, $N_{EDC/NP\,(50)}$ is the number of EDC molecules added per NP at the midpoint of the curve, and p is the slope at the inflection point.

Supplementary Table 13: Fitting parameters corresponding to Supplementary Figure 27 and Supplementary Figure 28 for the covalent binding of HSA and aTR to NP-F/COOH, respectively. $\Delta r_h = r_{h,max} - r_{h,min}$ = increase of the NP radius upon saturation conditions, i.e. when the NP surface has been covered as much as possible with proteins, which corresponds to the maximum thickness of the protein layer.

parameter	fit values - HSA	fit values - aTR
$r_{h,max}$ [nm]	11.50 ± 0.10	11.78 ± 0.09
$r_{h,min}$ [nm]	9.60 ± 0.09	9.41 ± 0.12
$N_{EDC/NP\ (50)}$	(6.66 ± 0.50)·10^3	(1.66 ± 0.15)·10^3
p	4.94± 1.59	2.84± 0.60
Δr_h [nm]	1.90 ± 0.19	2.37 ± 0.21

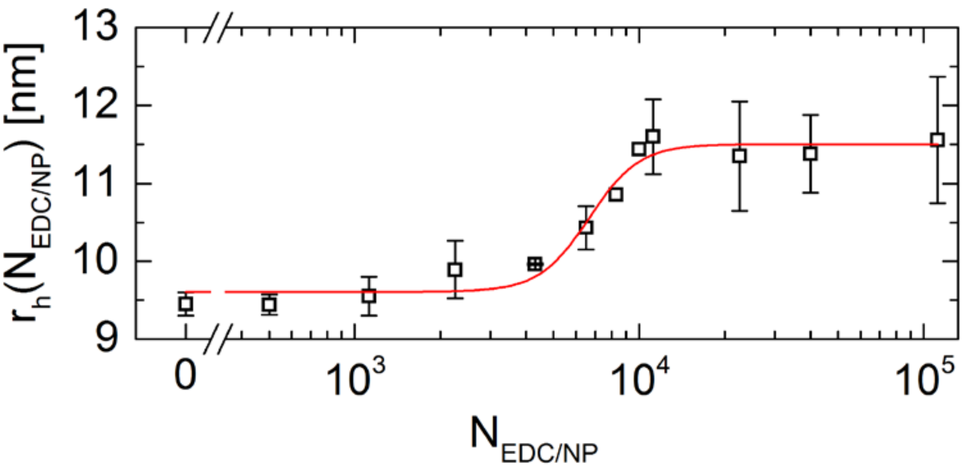

Supplementary Figure 27: Mean values ± standard deviation of the hydrodynamic radii r_h for NP-F/COOH covalently conjugated with increasing numbers of HSA molecules (*i.e.*, increasing number of EDC molecules per NP, *cf.* Supplementary Table 9) and the corresponding fit (red line).

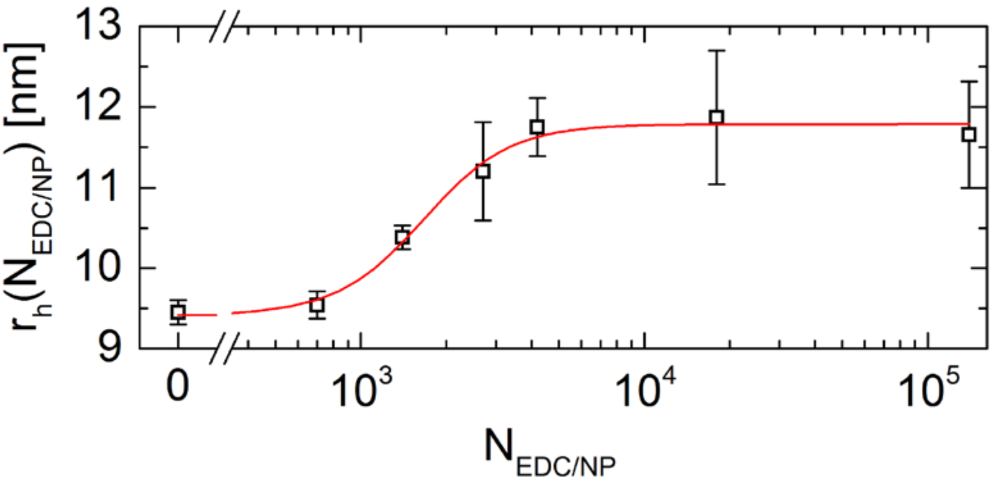

Supplementary Figure 28: Mean values ± standard deviation of the hydrodynamic radii for NP-F/COOH covalently conjugated with increasing numbers of aTR molecules (*i.e.*, increasing number of EDC molecules per NP, *cf.* Supplementary Table 10) and the corresponding fit (red line).

Size correlation for HSA or TR naturally adsorbed onto NP-F/NH$_2$@PMA

Radii of samples of HSA or TR naturally adsorbed onto NP-F/NH$_2$@PMA, which were obtained by diffusion experiments present a sigmoidal distribution, as shown in Supplementary Figure 29 and 30, which were fitted with a Hill model (OriginLab), *cf.* fitting parameters in Supplementary Table 14, as follows [15,16]:

$$r_h(N_P) = r_h(0) \sqrt[3]{1 + \frac{V_P}{\frac{4\pi}{3} r_h^3(0)} \cdot N_P} \qquad \text{Supplementary Equation 7}$$

Where P stands for Protein (HSA or TR) and the number of proteins per NP (N_P) is given by the Hill equation,

$$N_P = \frac{N_{max}}{1 + \left(\frac{K_d'}{c_P}\right)^n} \qquad \text{Supplementary Equation 8}$$

Supplementary Table 14: Compilation of fit parameters as obtained from the diffusion data shown in Supplementary Figures 29 and 30. $r_h(0)$ is the hydrodynamic diameter of the NPs without HSA or TR added, as obtained from fitting the measured $r_h(c)$ data points with the Hill model. The fit also returns the parameters K'_d, N_{max}, Δr_h and n, which are the apparent dissociation coefficient, maximum number of proteins bound per NP, corona thickness, *i.e.*, the difference in radius of NPs saturated with proteins and NPs without proteins, and the Hill coefficient, respectively.

fit parameters	Values for HSA	Values for TR
$r_h(0)$ [nm]	7.62 ± 0.08	9.16 ± 0.28
Δr_h [nm]	4.87 ± 0.08	9.22 ± 0.30
K_d' [μM]	13.6 ± 0.9	4.1 ± 0.7
N_{max}	65.6 ± 2.6	191.0 ± 11.4
n	2.3 ± 0.2	1.2 ± 0.1

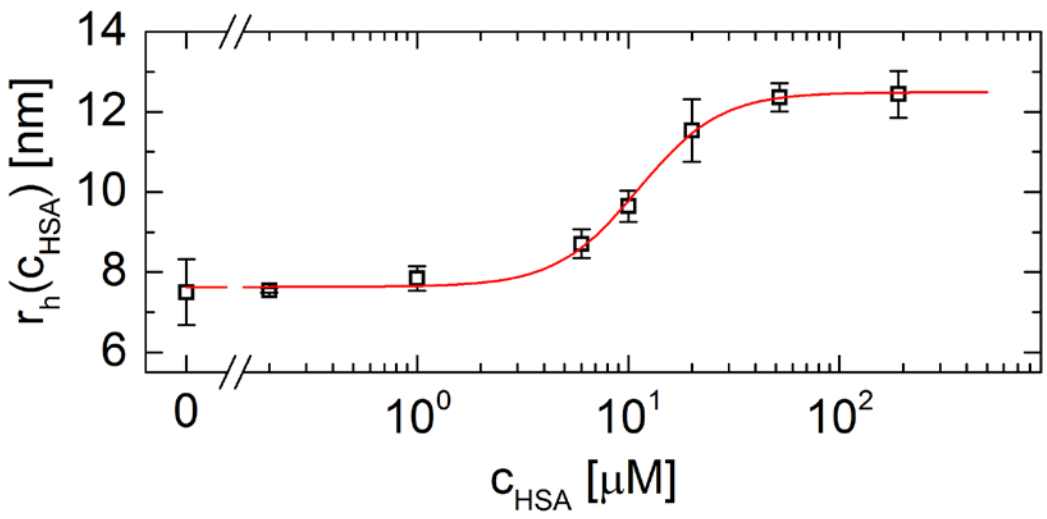

Supplementary Figure 29: Hydrodynamic radius r_h ± standard deviation as measured for the different NP-protein complexes upon the presence of different concentrations c_{HSA} of HSA in PBS, and the corresponding fits based on the Hill model. The fit parameters are shown in Supplementary Table 14.

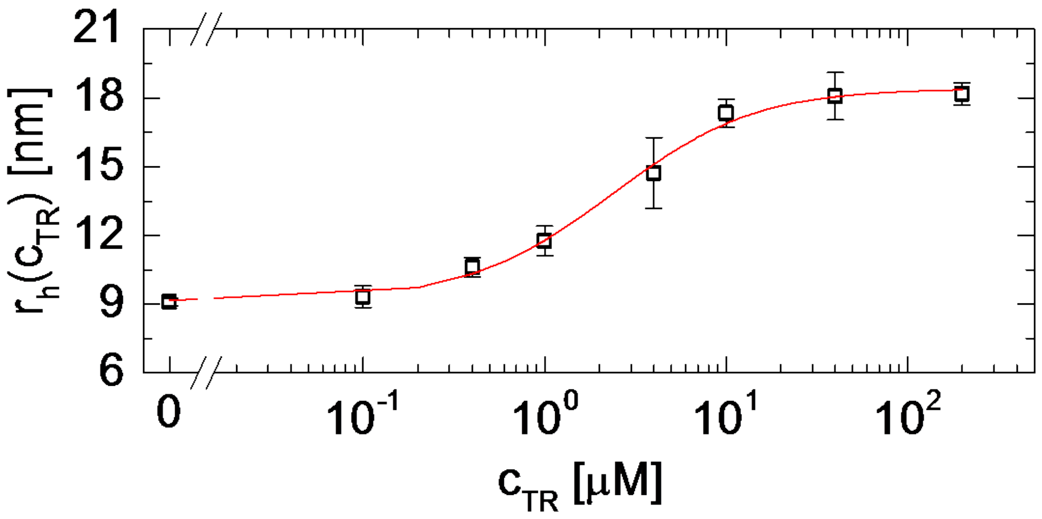

Supplementary Figure 30: Hydrodynamic radius r_h ± standard deviation as measured for the different NP-protein complexes upon the presence of different concentrations c_{TR} of TR in PBS, and the corresponding fits based on the Hill model. The fit parameters are shown in Supplementary Table 14.

Size correlation for NP-F/COOH covalently linked with either HSA or aTR by DLS

Samples of covalent binding of HSA or aTR onto NP-F/COOH were analyzed by dynamic light scattering (DLS) at 173° in water using a Nano ZS (Malvern, Worcesterhire, UK) equipped with a 633 nm laser. Results are shown in Supplementary Figure 31 and Supplementary Figure 32 for

HSA and aTR, respectively. Raw data of the mean r_h for the DLS distributions in number ($r_{h(N)}$), volume ($r_{h(V)}$), and intensity ($r_{h(I)}$) are shown in Supplementary Table 15 and Supplementary Table 16 for HSA and aTR, respectively.

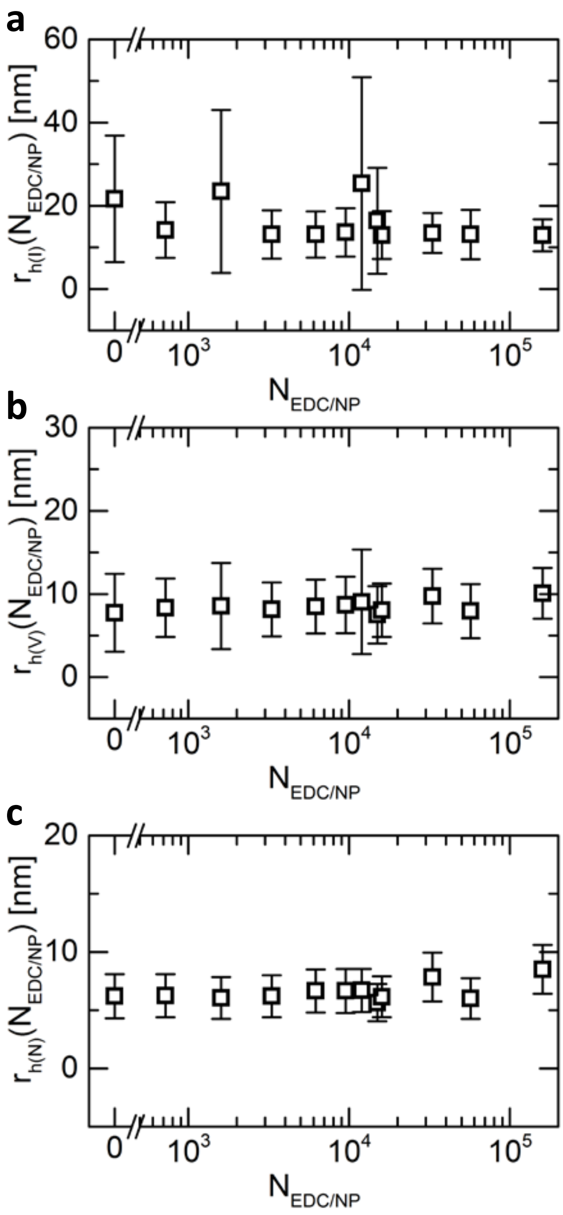

Supplementary Figure 31: Mean hydrodynamic radius ± standard deviation derived from the DLS distributions in **a** intensity, **b** volume and **c** number for HSA covalently attached to NP-F/COOH by using increasing numbers of EDC molecules per NP.

Supplementary Table 15: Hydrodynamic radius r_h (mean value ± SD) derived from DLS measurements of the different NP-F/COOH covalently conjugated with increasing number of HSA molecules. Data correspond to the raw data shown in Supplementary Figure 31. PDI is the polydispersity index.

Sample	$N_{EDC/NP}$	$N_{HSA/NP}$	$r_{h(I)}$ [nm]	$r_{h(V)}$ [nm]	$r_{h(N)}$ [nm]	PDI
NP_HSA_0_cov	0	300	21.6 ± 15.2	7.7 ± 4.7	6.2 ± 1.9	0.24
NP_HSA_1_cov	720	300	14.1 ± 6.7	8.3 ± 3.5	6.2 ± 1.8	0.20
NP_HSA_2_cov	1600	300	23.4 ± 19.6	8.5 ± 5.2	6.0 ± 1.8	0.27
NP_HSA_3_cov	3300	300	13.1 ± 5.8	8.1 ± 3.2	6.2 ± 1.8	0.17
NP_HSA_4_cov	6200	300	13.1 ± 5.5	8.5 ± 3.2	6.6 ± 1.8	0.17
NP_HSA_5_cov	9500	300	13.6 ± 5.8	8.7 ± 3.4	6.6 ± 1.9	0.18
NP_HSA_6_cov	12000	300	25.3 ± 25.5	9.0 ± 6.3	6.7 ± 1.8	0.26
NP_HSA_7_cov	15000	300	16.4 ± 12.7	7.5 ± 3.4	5.6 ± 1.6	0.24
NP_HSA_8_cov	16000	300	12.9 ± 5.7	8.0 ± 3.2	6.1 ± 1.7	0.14
NP_HSA_9_cov	33000	300	13.4 ± 4.8	9.7 ± 3.3	7.8 ± 2.1	0.20
NP_HSA_10_cov	57000	300	13.0 ± 5.9	7.9 ± 3.2	6.0 ± 1.7	0.15
NP_HSA_11_cov	160000	300	12.9 ± 3.8	10.1 ± 3.0	8.5 ± 2.1	0.08

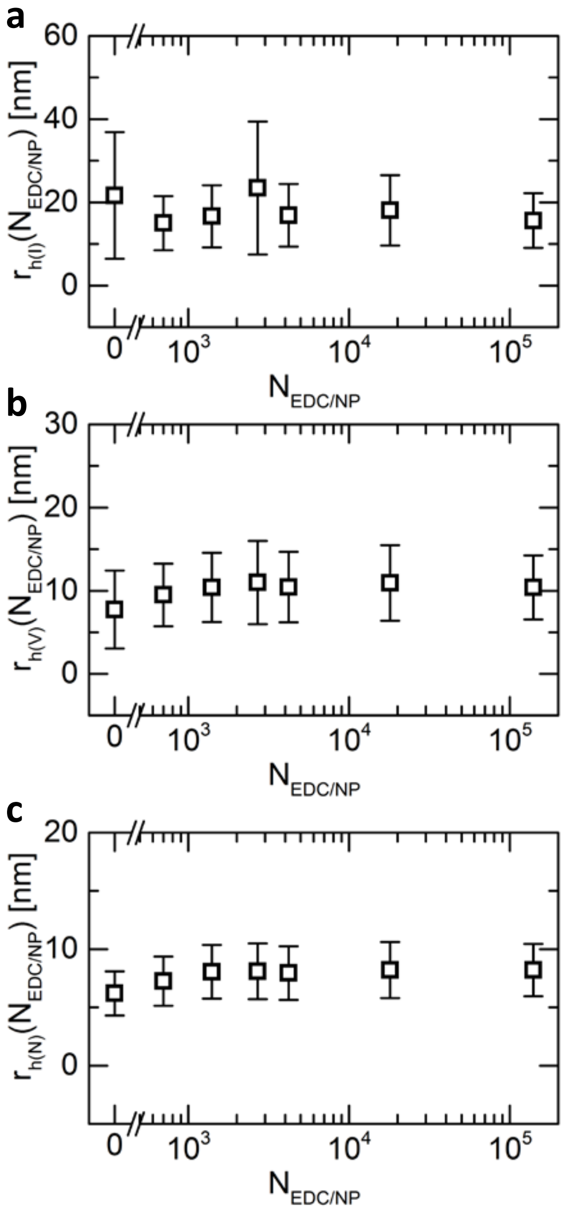

Supplementary Figure 32: Mean hydrodynamic radii ± standard deviation derived from the DLS distributions in **a** intensity, **b** volume, and **c** number for aTR covalently attached to NP-F/COOH, by using increasing numbers of EDC molecules per NP.

Supplementary Table 16: Hydrodynamic radii r_h (mean value ± SD) as derived from DLS measurements of the different NP-F/COOH covalently conjugated with increasing number of aTR molecules. Data correspond to the raw data shown in Supplementary Figure 32.

Sample	$N_{EDC/NP}$	$N_{aTR/NP}$	$r_{h(I)}$ [nm]	$r_{h(V)}$ [nm]	$r_{h(N)}$ [nm]	PDI
NP_aTR_0_cov	0	300	21.6 ± 15.2	15.5 ± 4.7	6.2 ± 1.9	0.24
NP_aTR _1_cov	700	300	15.0 ± 6.5	9.5 ± 3.7	7.2 ± 2.1	0.15
NP_aTR _2_cov	1400	300	16.6 ± 7.4	10.4 ± 4.1	8.0 ± 2.3	0.22
NP_aTR _3_cov	2700	300	23.4 ± 16.0	11.0 ± 5.0	8.1 ± 2.4	0.36
NP_aTR _4_cov	4200	300	16.9 ± 7.5	10.4 ± 4.2	7.9 ± 2.3	0.15
NP_aTR _5_cov	18000	300	18.1 ± 8.4	10.9 ± 4.5	8.2 ± 2.4	0.25
NP_aTR _6_cov	140000	300	15.6 ± 6.6	10.4 ± 3.8	13.4 ± 3.7	0.23

Size correlation for HSA or TR naturally adsorbed onto NP-F/NH_2@PMA by DLS

Samples of natural adsorption of HSA or TR onto NP-F/NH_2@PMA were analyzed by dynamic light scattering (DLS) at 173° in water using a Nano ZS (Malvern, Worcesterhire, UK) equipped with a 633 nm laser, with the following results, as shown in Supplementary Figure 33 and 34. Raw data of the mean r_h for the DLS distributions in number ($r_{h(N)}$), volume ($r_{h(V)}$), and intensity ($r_{h(I)}$) are shown in Supplementary Table 17 and 18. The actual DLS distributions are shown in Supplementary Figure 35-38.

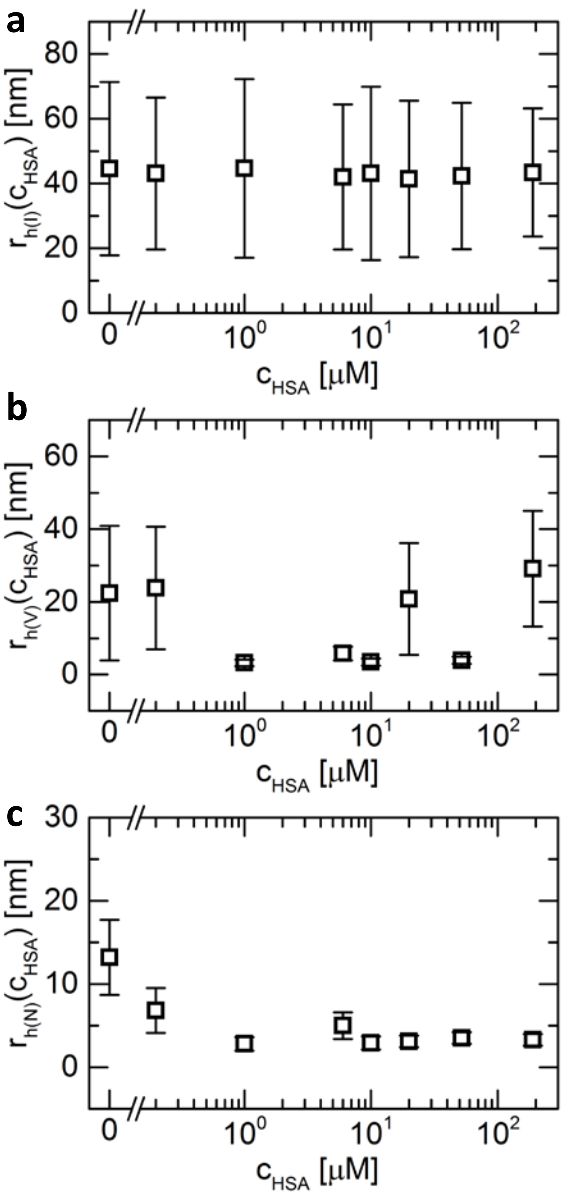

Supplementary Figure 33: Mean hydrodynamic radii ± standard deviation derived from the DLS distributions in **a** intensity, **b** volume, and **c** number for HSA naturally absorbed to NP-F/NH_2@PMA, by using increasing concentrations of HSA.

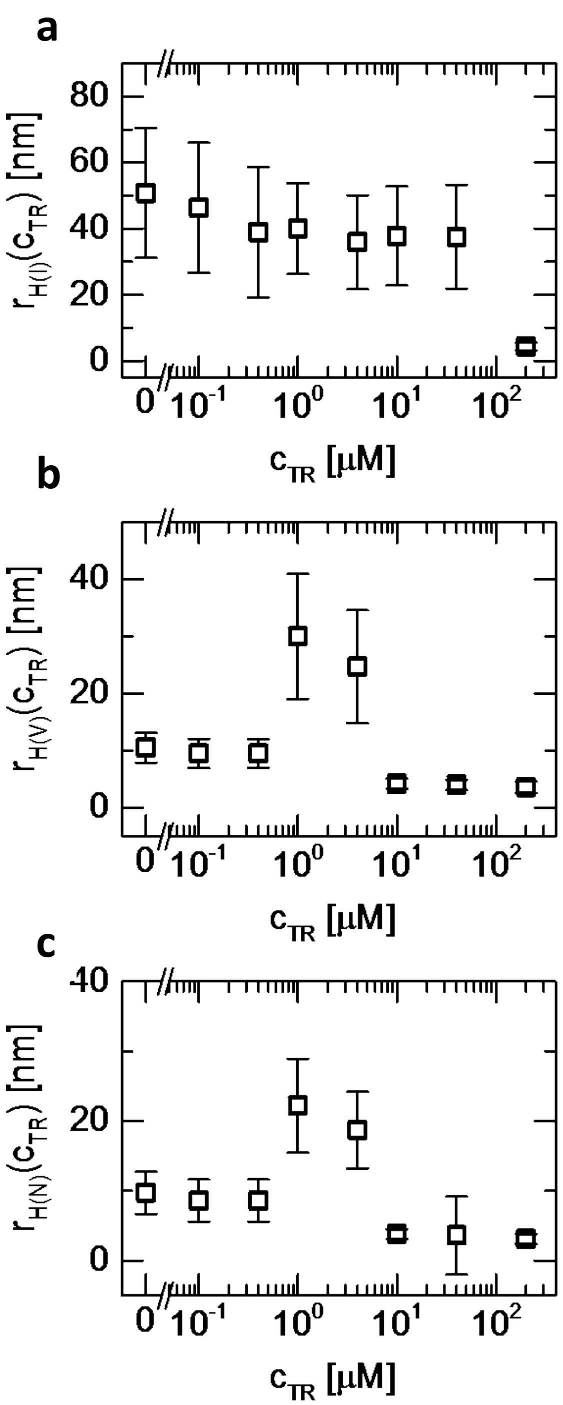

Supplementary Figure 34: Mean hydrodynamic radii ± standard deviation derived from the DLS distributions in **a** intensity, **b** volume, and **c** number for TR naturally absorbed to NP-F/NH_2@PMA, by using increasing concentrations of TR.

Supplementary Table 17: Mean hydrodynamic radii from the intensity $r_{h(I)}$, volume $r_{h(V)}$, and number $r_{h(N)}$ distributions (mean value ± SD) of the different NP-F/NH_2@PMA exposed at increasing concentrations of HSA. Data correspond to the raw data shown in Supplementary

Figure 33. Notice the mean values from the most populated species were used, as in most cases two peaks appeared (*i.e.*, from the NPs and the free HSA).

Sample	c_{HSA} [µM]	$r_{h(I)}$ [nm]	$r_{h(V)}$ [nm]	$r_{h(N)}$ [nm]	PDI
NP_HSA_0_ads	0	44.6 ± 26.8	22.4 ± 18.5	13.2 ± 4.5	0.24
NP_HSA_1_ads	0.2	43.1 ± 23.5	23.8 ± 16.9	6.8 ± 2.7	0.24
NP_HSA_2_ads	1	44.7 ± 27.6	3.2 ± 0.9	2.8 ± 0.8	0.26
NP_HSA_3_ads	6	42.0 ± 22.4	5.8 ± 1.9	5.0 ± 1.6	0.32
NP_HSA_4_ads	10	43.1 ± 26.8	3.4 ± 1.0	2.9 ± 0.8	0.29
NP_HSA_5_ads	20	41.4 ± 24.2	20.8 ± 15.4	3.1 ± 0.7	0.41
NP_HSA_6_ads	52	42.3 ± 22.6	3.9 ± 1.0	3.5 ± 0.7	0.46
NP_HSA_7_ads	190	43.4 ± 19.8	29.1 ± 15.9	3.3 ± 0.7	0.60

Supplementary Table 18: Mean hydrodynamic radii from the intensity $r_{h(I)}$, volume $r_{h(V)}$, and number $r_{h(N)}$ distributions (mean value ± SD) of the different NP-F/NH_2@PMA exposed at increasing concentrations of TR. Data correspond to the raw data shown in Supplementary Figure 34. Notice the mean values from the most populated species were used, as in most cases two peaks appeared (*i.e.*, from the NPs and the free TR).

Sample	c_{TR} [µM]	$r_{h(I)}$ [nm]	$r_{h(V)}$ [nm]	$r_{h(N)}$ [nm]	PDI
NP_TR_0_ads	0	50.8 ± 19.6	10.5 ± 2.6	9.7 ± 3.0	0.21
NP_TR_1_ads	0.1	46.3 ± 19.7	9.5 ± 2.5	8.6 ± 3.0	0.19
NP_TR_2_ads	0.4	38.9 ± 16.2	26.7 ± 11.1	20.1 ± 5.9	0.16
NP_TR_3_ads	1	40.0 ± 13.7	30.0 ± 11.0	22.2 ± 6.7	0.16
NP_TR_4_ads	4	35.9 ± 14.2	24.7 ± 9.9	18.7 ± 5.5	0.22
NP_TR_5_ads	10	37.8 ± 15.0	4.2 ± 0.9	3.8 ± 0.7	0.26
NP_TR_6_ads	40	37.5 ± 15.7	4.0 ± 0.9	3.6 ± 5.6	0.49
NP_TR_7_ads	200	4.4 ± 1.2	3.6 ± 1.0	3.1 ± 0.7	0.42

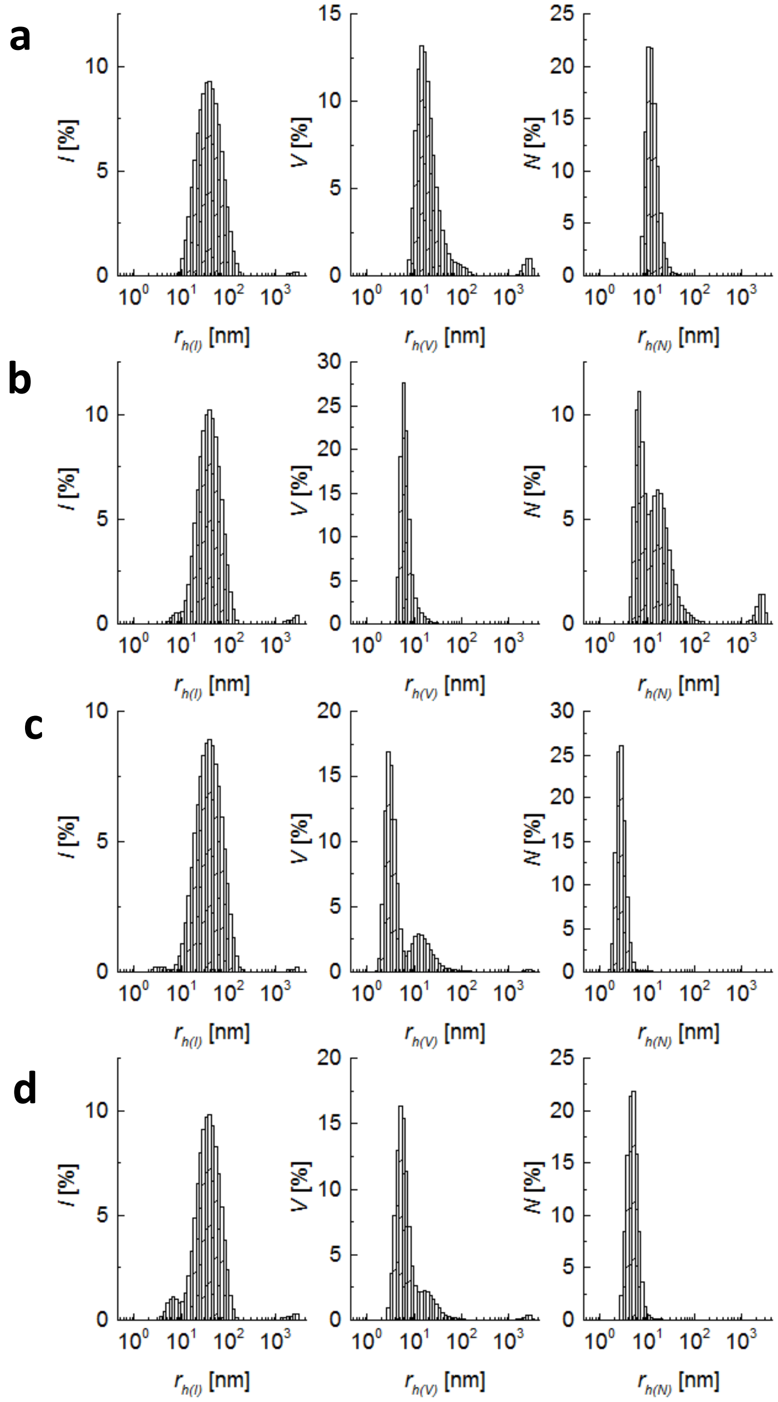

Supplementary Figure 35: DLS intensity, volume, and number distributions of samples exposed to different concentrations of HSA, *i.e.*, **a** 0 mM, **b** 0.0002 mM, **c** 0.001 mM and **d** 0.006 mM.

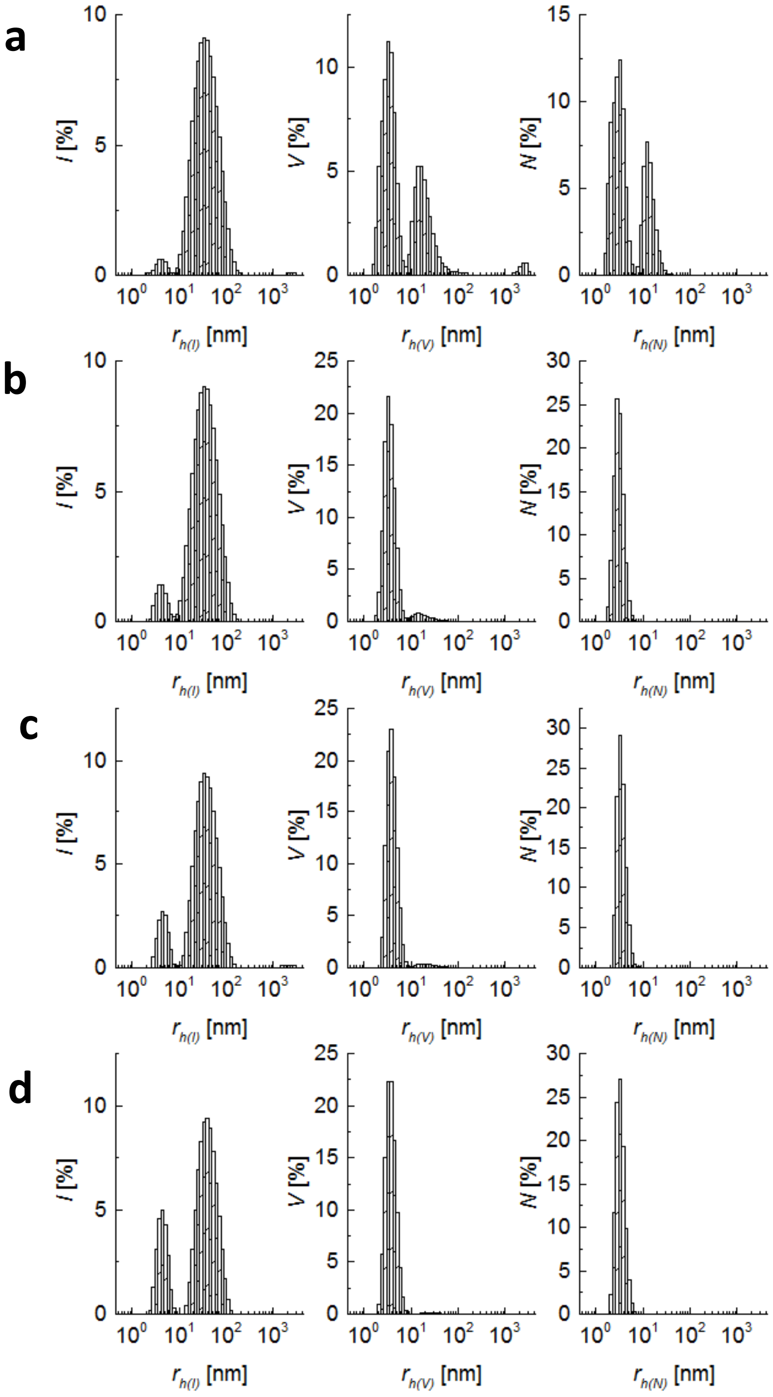

Supplementary Figure 36: DLS distributions of samples exposed to different concentrations of HSA, *i.e.*, **a** 0.01 mM, **b** 0.02 mM, **c** 0.05 mM and **d** 0.19 mM.

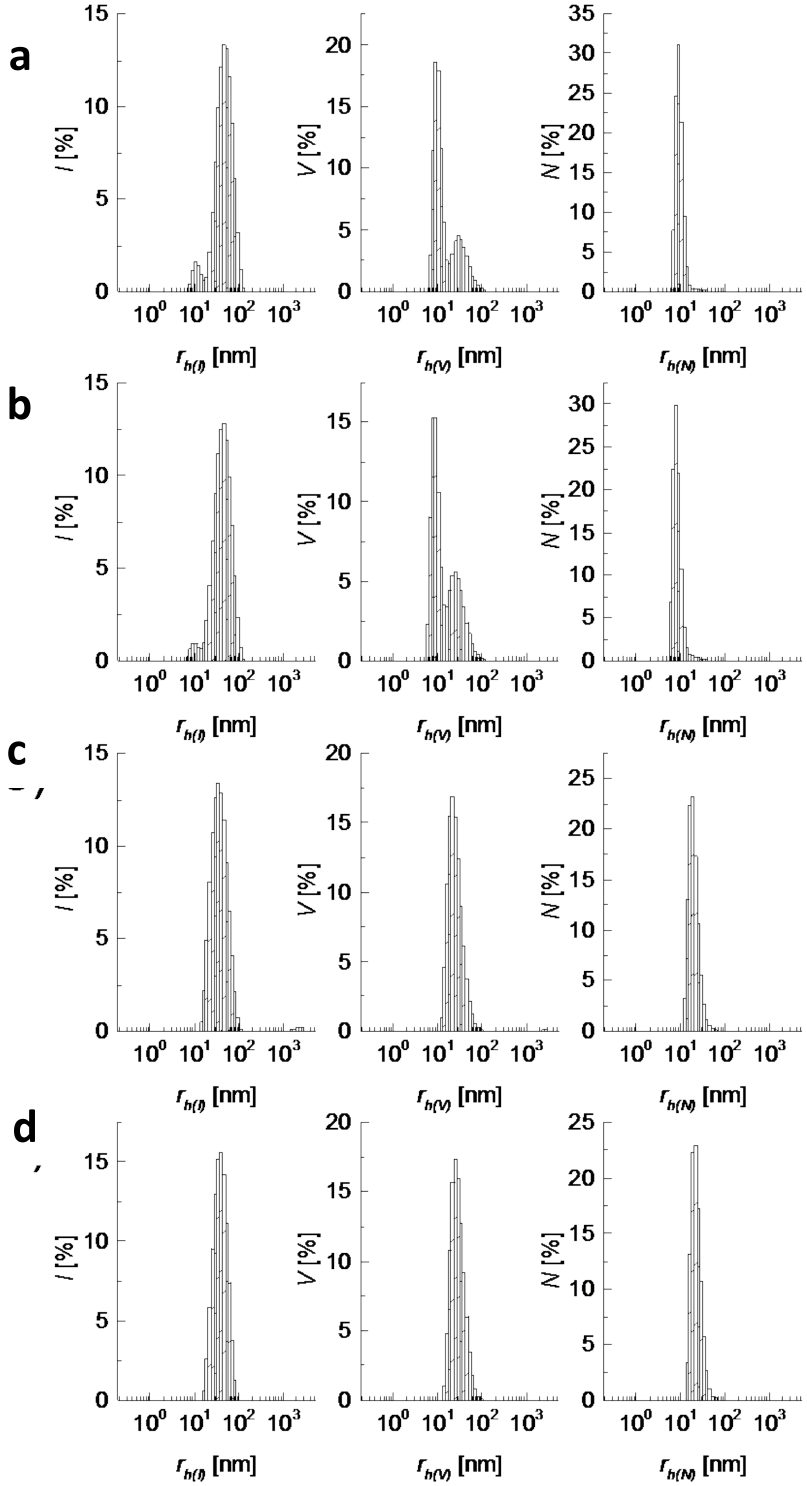

Supplementary Figure 37: DLS distributions of samples exposed to different concentrations of TR, *i.e.*, **a** 0 mM, **b** 0.0001 mM, **c** 0.0004 mM and **d** 0.001 mM.

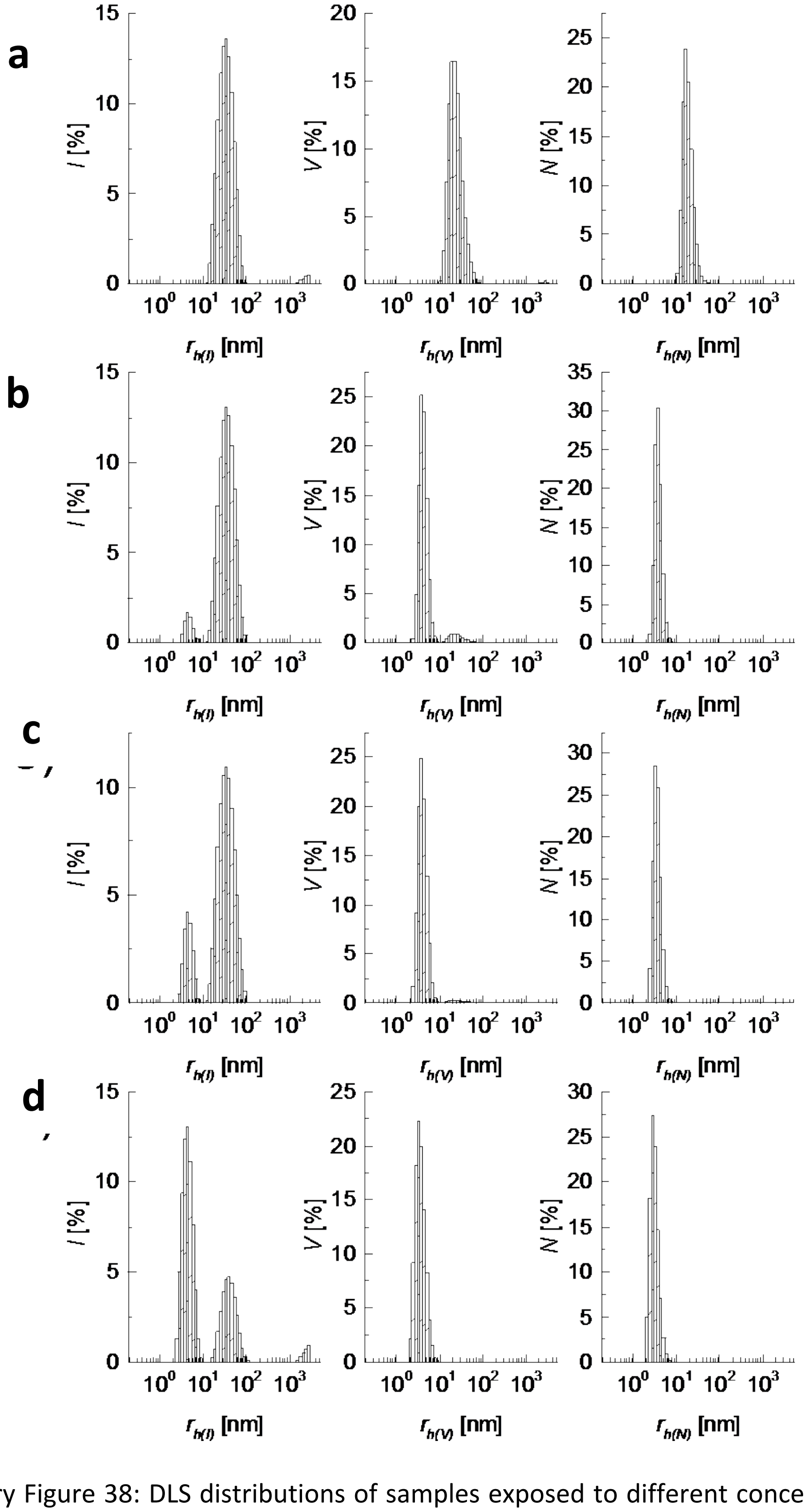

Supplementary Figure 38: DLS distributions of samples exposed to different concentrations of TR, *i.e.*, **a** 0.004 mM, **b** 0.01 mM, **c** 0.04 mM and **d** 0.2 mM.

Diffusion constant and size measurements in blood by ^{19}F-NMR

Blood was extracted by authorized personnel at Policlínica Gipuzkoa (San Sebastián, Spain) from a single donor and co-author of the present work (C. Carrillo-Carrión). Blood samples were collected in citrate loaded 4.5 mL tubes (Vacuette, 9NC coagulation sodium citrate 3.2 %, 13 x 75, blue cap). Part of the blood was directly mixed with NPs in experiments type I that will be explained later. Due to the use of sodium citrate as anti-clotting agent no clots were formed and thus no NPs could stick to clots. The rest of the blood was centrifuged at 1500 g for 10 min at 10 °C and the top plasma fraction was collected in aliquots of 400 µL and stored at -20 °C for 48 hours until further use in experiments type II.

NP-F/COOH, NP-F/NH_2 and NP-F/NH_2@PMA were transferred to PBS and concentrated with amicon filters (MWCO 100 kDa) down to NP concentrations of 5 µM (NP-F/COOH) and 6 µM (NP-F/NH_2 and NP-F/NH_2@PMA). Then, NPs were separately incubated with either whole blood or plasma.

Experiment type I: NP incubation with whole blood

In separate 1.5 mL Eppendorf vials, 735 µL of freshly extracted whole blood were mixed with 65 µL of a PBS solution containing either NP-F/COOH, NP-F/NH_2 or NP-F/NH_2@PMA at the aforementioned concentrations. The mixture was incubated at 37 °C for 30 min, gently shaking at 130 rpm. After that time, phase separation between plasma and blood cells was already starting to take place. Each vial was then centrifuged at 10^3 g for 8 minutes at 10 °C and the plasma with the NPs dispersed in it was separated and transferred to an NMR tube for diffusion measurements (*cf.* Supplementary Figure 39).

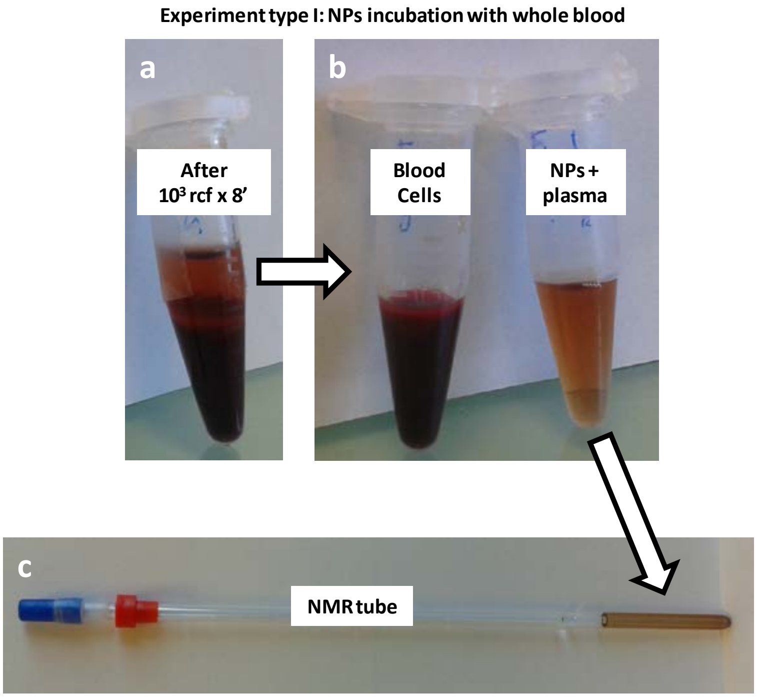

Supplementary Figure 39: **a** Photographs of NPs incubated with blood for 30 min at 37 °C and centrifuged (1000 g for 8 min), in which phase separation is clearly observed. **b** Subsequent phase separation between blood cells (bottom fraction) and plasma with NPs dispersed (top

fraction). **c** Fraction with plasma with NP as introduced in a NMR tube with a coaxial insert filled with TFA in D_2O ready for diffusion measurements.

Experiment type II: NP incubation with plasma

400 µL aliquots of previously separated plasma were mixed with 65 µl of a PBS solution containing either NP-F/COOH, NP-F/NH_2 or NP-F/NH_2@PMA at the aforementioned concentrations. The so-obtained mixture was transferred to an NMR tube for diffusion measurements.

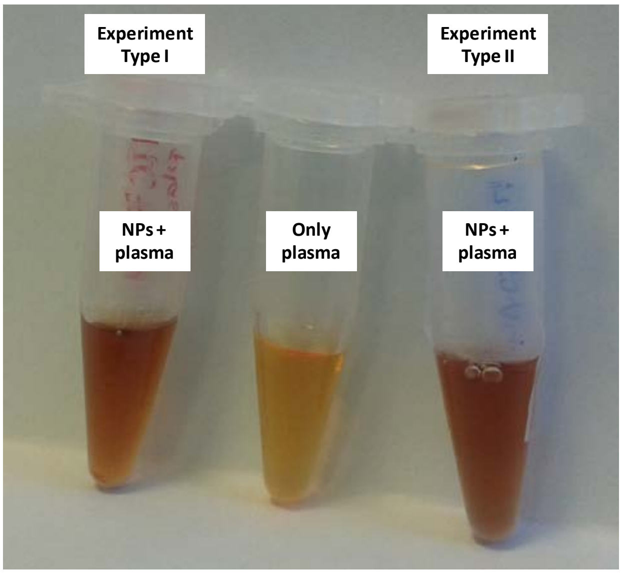

Supplementary Figure 40: (from left to right) Photographs for comparison between plasma with NPs obtained after NP incubation with whole blood and after centrifugation (experiment type I), only plasma without NPs, and plasma mixed with NPs (experiment type II). The plasma samples with NPs have a darker color due to the NP presence.

Diffusion constant (D) measurement by ^{19}F diffusion NMR in blood

Diffusion experiments were measured as described before using 5 mm standard NMR tubes with a coaxial insert carrying TFA in D_2O (0.024 % v/v). The diffusion measurements were carried out at 37 °C to mimic physiological conditions as much as possible. Before every diffusion experiment, a standard ^{19}F-NMR spectrum of about 10 minutes was run, to let the sample reach 37 °C and stabilize before running the diffusion experiment. The diffusion constant values *D* were then obtained from the signal intensity decay fitting to a mono exponential equation as described previously. The obtained results are summarized below.

Supplementary Table 19: Diffusion constant values *D* obtained for each type of NP after incubation with either whole blood (experiment type I) or only plasma (experiment type II), and the corresponding standard deviation (SD). The *n* value refers to the number of independent measurements performed in each type of medium.

NP type	Medium	*D* [10^{-11} m^2/s]	*n*
NP-F/COOH	HEPES	2.59 ± 0.04	6
NP-F/COOH	BLOOD	2.70 ± 0.05	2
NP-F/COOH	PLASMA	2.88 ± 0.20	2
NP-F/NH_2	PBS	2.79 ± 0.08	3
NP-F/NH_2	BLOOD	3.19 ± 0.15	2
NP-F/NH_2	PLASMA	4.28 ± 0.51	3
NP-F/NH_2@PMA	PBS	3.29 ± 0.30	3
NP-F/NH_2@PMA	BLOOD	2.31 ± 0.03	2
NP-F/NH_2@PMA	PLASMA	1.59 ± 0.05	2

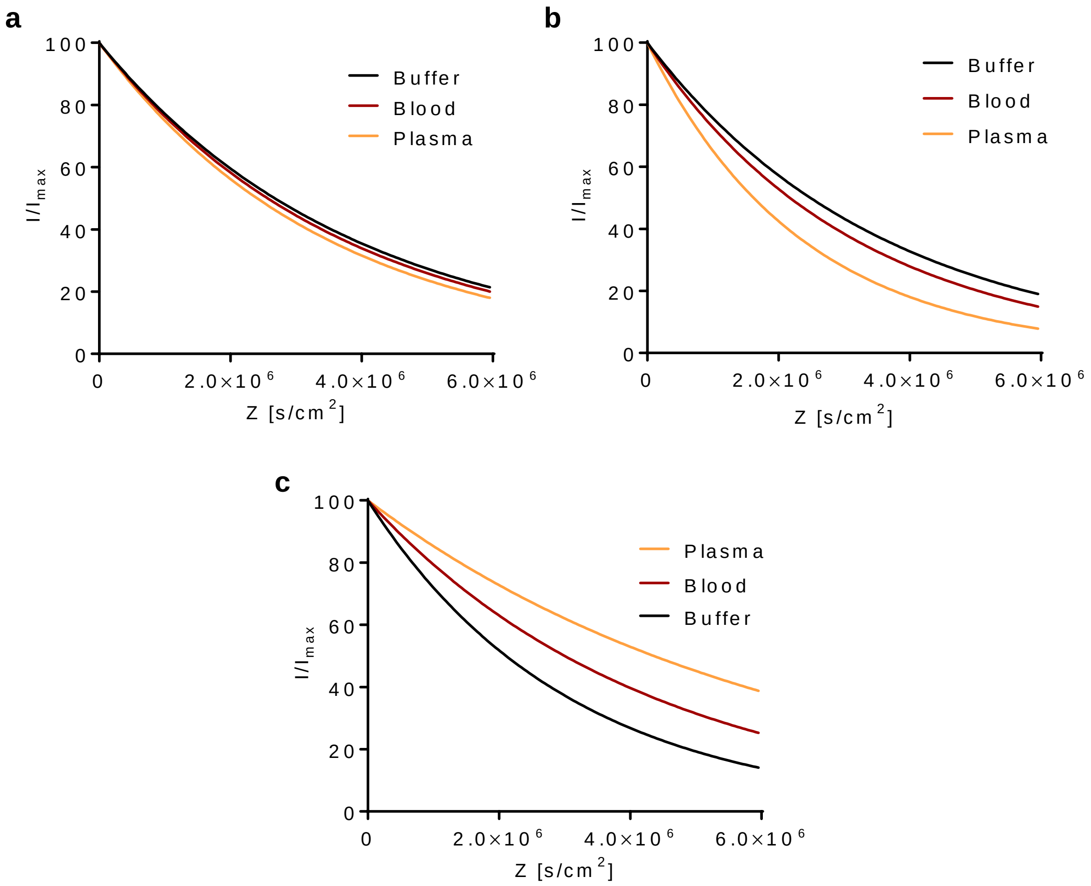

Supplementary Figure 41: Plot of normalized fitted mean mono exponential decay curves for: **a** NP-F/COOH and **b** NP-F/NH_2 and **c** NP-F/NH_2@PMA in buffer (HEPES or PBS), whole blood or plasma.

Plasma viscosity calculation

For an accurate size calculation according to the Einstein-Stokes relation (Supplementary Equation 5), it is important to know the viscosity η of the medium. In the present case, plasma isolated from blood from a single donor was used, that was treated with an anticoagulant solution (sodium citrate 3.2 %), in a theoretical ratio of 9:1 blood:citrate solution, which may obviously vary depending on the exact amount of blood extracted per tube. Moreover, that

plasma was diluted with PBS by the addition of 65 µL of a NP solution in PBS, hence the plasma content in the samples was approximately 75% of the total volume. For this reason, it came apparent that literature values for plasma dynamic viscosity, which often range from 0.0011-0.0013 $kg{\cdot}m^{-1}s^{-1}$, might not be suitable for the calculations, as they could only give a rough estimation. Hence, it was envisaged that performing diffusion experiments with a small molecule that would not interfere with the surrounding environment could be used for viscosity measurements, e.g. diluted TFA. The idea was to measure the value of the diffusion constant *D* for TFA by ^{19}F diffusion NMR in two different media, out of which one would be plasma of unknown viscosity and the other one would be one of known viscosity, such as water. Hence, by assuming that TFA does not change size when placed in water or plasma and applying Supplementary Equation 5, it should be possible to calculate the viscosity of plasma (Supplementary Figure 42).

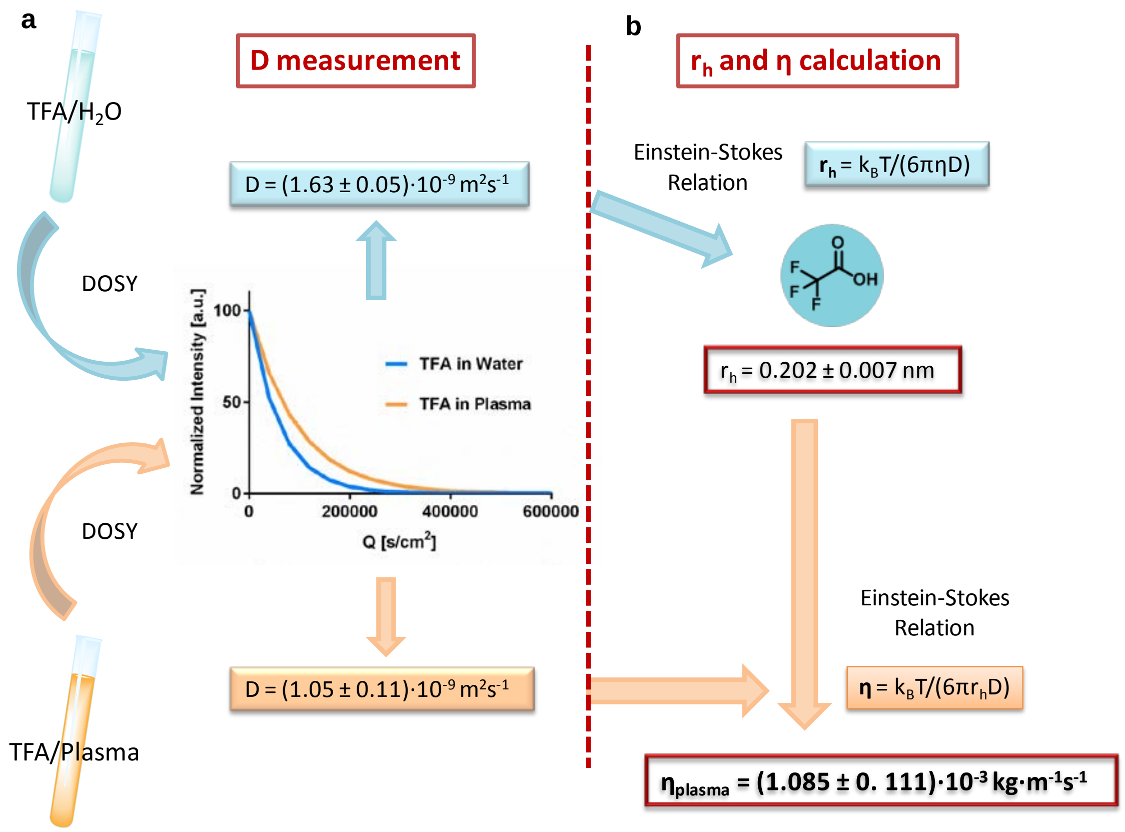

Supplementary Figure 42: Work-flow for the calculation of plasma viscosity by measuring diffusion of TFA in both water and plasma. **a** Firstly, diffusion constants *D* for TFA in each medium were obtained by ^{19}F diffusion NMR experiments. **b** Then, by introducing the value of the diffusion constant *D* in water into the Einstein-Stokes relation it is possible to obtain the hydrodynamic radius r_h of TFA. With this r_h value and the *D* value for TFA in plasma it is possible to obtain the viscosity of plasma η_{plasma}.

Diffusion experiments for TFA were run in 5 mm standard NMR tubes with a coaxial insert carrying only D_2O. The diffusion parameters were adapted for TFA, since it is much smaller than the NPs measured before. Experiments were run first in PBS at 25 °C and secondly in plasma treated with citrate and diluted with 65 µL of PBS at 37 °C in the same exact manner as

performed before in experiments type I and II. The final concentration of TFA in all cases was 0.005 % (v/v).

Diffusion measurements were performed as described before, but with the following parameters: 16k acquisition points, SW 15 ppm, NS 32, DS 32, D1 2 s, D20 (gradient length) 0.1 s, P30 (diffusion delay/2) 0.8 ms and 12 equally spaced gradient strengths from 5 to 95%. NMR measurements were performed spinning and using deuterium lock.

By measuring the diffusion constant for TFA in PBS at 25 °C ($D = (1.63 \pm 0.05)\cdot10^{-9}\ m^2s^{-1}$) and applying the Einstein-Stokes relation, a hydrodynamic radius r_h for TFA of 0.202 ± 0.007 nm was obtained as an average of 4 measurements. Subsequently, the diffusion constant for TFA in plasma treated with citrate and diluted with PBS at 37 °C was measured to be $(1.05 \pm 0.11)\cdot10^{-9}\ m^2s^{-1}$ as an average of 7 measurements. Assuming that TFA does not change size when placed in plasma and applying the Einstein-Stokes equation a value of dynamic viscosity for diluted plasma of $\eta_{plasma} = 0.001085 \pm 0.000111\ kg\cdot m^{-1}s^{-1}$ was obtained, which is close to literature values, but somehow lower, as expected for a diluted sample.

With this viscosity value the size of NP-F/COOH, NP-F/NH_2 and NP-F/NH_2@PMA after incubation with either blood or plasma was calculated, using the diffusion constants previously measured (*cf*. Supplementary Table 19).

Supplementary Table 20: Values of hydrodynamic radii r_h as obtained from experimentally measured diffusion constants *D* for NPs incubated with blood or plasma, and the corresponding standard deviation (SD). The *n* value refers to the number of independent measurements performed for each medium.

NP type	Medium	r_h [nm]	*n*
NP-F/COOH	HEPES	9.45 ± 0.15	6
NP-F/COOH	BLOOD	7.74 ± 0.14	2
NP-F/COOH	PLASMA	7.28 ± 0.42	2
NP-F/NH_2	PBS	8.79 ± 0.25	3
NP-F/NH_2	BLOOD	6.58 ± 0.31	2
NP-F/NH_2	PLASMA	4.94 ± 0.56	3
NP-F/NH_2@PMA	PBS	7.50 ± 0.82	3
NP-F/NH_2@PMA	BLOOD	9.08 ± 0.13	2
NP-F/NH_2@PMA	PLASMA	13.21 ± 0.39	2

Natural adsorption of HSA onto NP-F/COOH and NP-F/NH_2

For comparison purposes with the results obtained in the presence of whole blood or plasma, and taking into account that HSA is the most abundant protein in human blood, the natural adsorption of HSA onto NP-F/COOH and NP-F/NH_2 was studied. Briefly, the NP diffusion constant *D* in the presence of a high concentration of HSA (1.5 mM) was measured by ^{19}F diffusion NMR and the r_h was obtained by introducing in the Einstein-Stokes relation (*cf*. Supplementary Equation 5) the so-obtained values of the diffusions constants *D* for each sample and the following parameters:

$T = 298$ K

$k_B = 1.38065 \cdot 10^{-23}\ m^2 kg \cdot s^{-2} K^{-1}$

$\eta_{HSA/H2O} = \eta_{H2O} \times (1 + c_{HSA} \times \eta_{HSA})$; $\eta_{HSA} = 4.2\ cm^3 g^{-1}$

Supplementary Table 21: Values for diffusion constants *D* and the derived hydrodynamic radii r_h for NP-F/COOH and NP-F/NH_2 incubated with HSA (0 and 1.5 mM), and the corresponding standard deviation. The *n* value refers to the number of independent measurements performed for each HSA concentration.

NP type	c_{HSA} [mM]	*D* [10^{-11} m^2/s]	r_h [nm]	*n*
NP-F/COOH	0	2.59 ± 0.04	9.45 ± 0.15	6
NP-F/COOH	1.5	1.87 ± 0.10	9.19 ± 0.49	2
NP-F/NH_2	0	2.79 ± 0.08	8.79 ± 0.25	3
NP-F/NH_2	1.5	2.38 ± 0.24	7.32 ± 0.78	2

In Supplementary Figure 43 the comparison between the hydrodynamic radii r_h as calculated for NP-F/COOH, NP-F/NH_2 and NP-F/NH_2@PMA in water, PBS, after incubation with whole blood, in the presence of plasma and in the presence of a high concentration of HSA (c_{HSA} = 0.19 mM for NP-F/NH_2@PMA and 1.5 mM for NP-F/COOH and NP-F/NH_2), is shown.

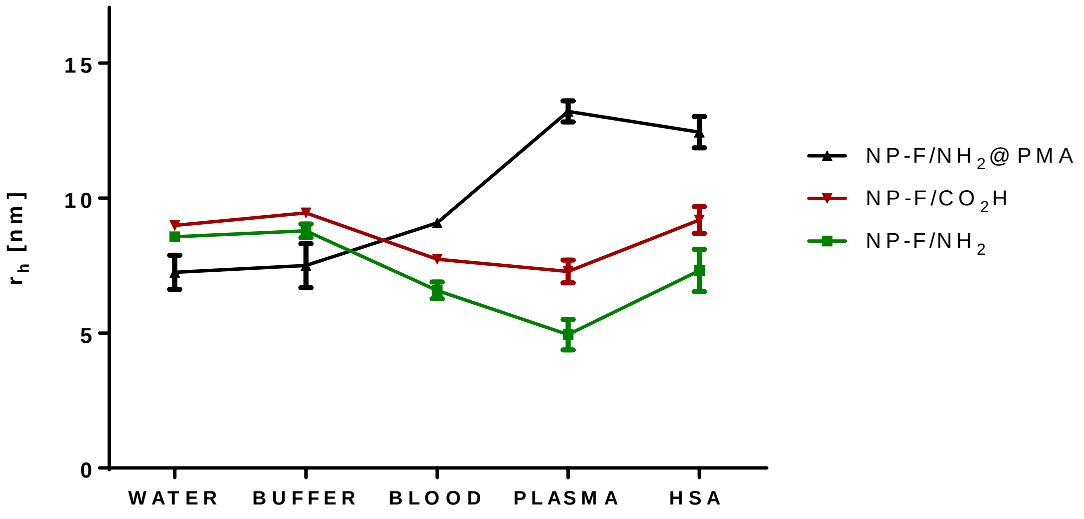

Supplementary Figure 43: Hydrodynamic radii r_h ± standard deviation for NP-F/COOH, NP-F/NH_2 and NP-F/NH_2@PMA in water, buffer (HEPES or PBS), blood, plasma, and in the presence of HSA (1.5 mM).

As it can be seen in Supplementary Figure 43, in the case of NP-F/COOH and NP-F/NH_2 placed in blood or plasma, a decrease in size was observed, which was more pronounced in the presence of plasma alone (*cf.* Supplementary Table 20). However, when the same NPs were placed in a 1.5 mM solution of HSA, the decrease in size was much less pronounced and for the case of NP-F/CO_2H it remained basically unchanged (*cf.* Supplementary Table 21).

On the contrary, in the case of NP-F/NH_2@PMA an increase of size was observed in all cases, particularly in the case of incubation with plasma (from 7.50 ± 0.82 to 13.21 ± 0.39 nm, Supplementary Table 20) where a similar size albeit higher than that obtained in the presence of HSA was measured (13.21 ± 0.39 vs. 12.44 ± 0.58, Supplementary Table 20 and 11). Interestingly, the NPs incubated in whole blood registered a much lower increase of size than when incubated with plasma or HSA (from 7.50± 0.82 to 9.08 ± 0.13 nm, Supplementary Table 20). All these results may suggest that NPs interact differently with whole blood, plasma or isolated proteins.

Influence of fluorine loading

To study the potential influence of the amount of fluorine present on the NP surface on the interaction with proteins, we prepared NPs that only had HS-PEG-F (NP-F) and NPs that had a mixture of 50 % of HS-PEG-F and 50% of HS-PEG-COOH (NP-F/COOH(50)) and compared them with the already prepared NP-F/COOH, that has 75 % of HS-PEG-F and 25 % of HS-PEG-COOH ligands attached. These NPs were prepared following the typical procedure described in Gold NP synthesis section for NP-F/COOH, only adjusting the amount of each ligand to the corresponding percentage.

The newly prepared NPs were characterized by TEM, negative staining, UV-Vis, and ζ-potential.

Transmission Electron Microscopy (TEM)

Inorganic core radii (r_C) of all types of gold NPs were obtained from analysis of TEM images, following the procedure detailed before. The following core radii were obtained for each type of NP: NP-F/COOH (50) $r_C = 1.5 \pm 0.8$ nm and NP-F $r_C = 1.9 \pm 0.5$ nm (Supplementary Figure 44). The organic ligand shell does not provide sufficient contrast and thus is not included in the core diameter.

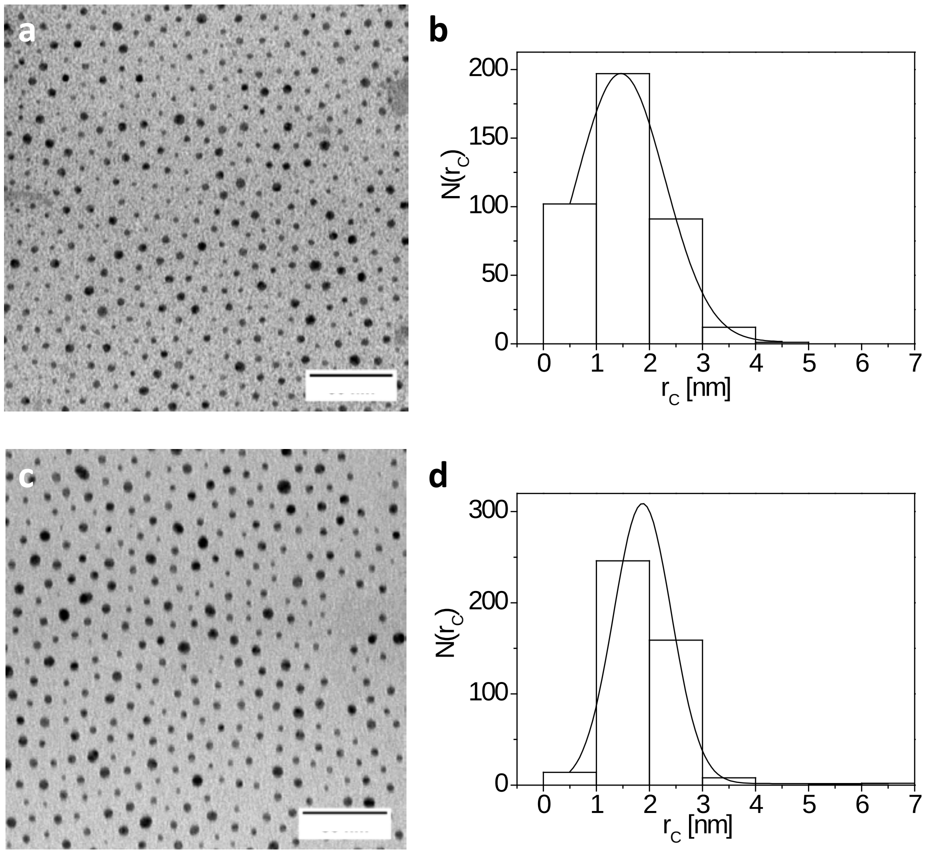

Supplementary Figure 44: Selected TEM micrographs (scale bar, 50 nm) and histograms for the size distribution of the Au NP core radius without organic shell (r_c) for NP-F/COOH (50) **a**-**b**; NP-F **c**-**d**. Histograms were obtained after analysis of at least 400 NPs.

Negative staining

The radii of the NP-organic shell system (r_{CS}) of NP-F/COOH (50) and NP-F were analyzed by negative staining TEM analysis, following the staining protocol with uranyl acetate described before. For morphological characterization of NP-organic shell system, the borders between the organic shells and the stained grid were automatically segmented by ImageJ[4,5] (function Analyzed Particles), *cf.* Supplementary Figure 45 and Supplementary Figure 46 for NP-F/COOH (50) and NP-F, respectively. The total area limited by the outlines was divided by the total number of NPs analyzed, to give a spherically-idealized mean area occupied by the NP-organic shell system (A_{CS}), from which the mean r_{CS} can be extracted (*i.e.*, $r_{CS}=\sqrt[2]{A_{CS}/\pi}$).

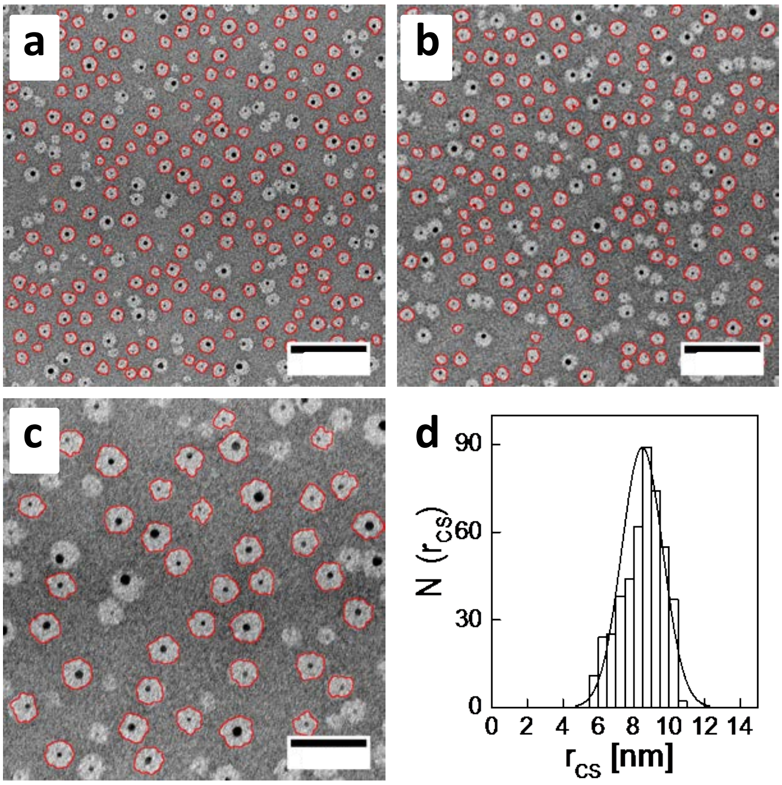

Supplementary Figure 45: **a-c** Selected low magnification negative staining TEM micrographs (**a-b** scale bar, 100 nm; **c** scale bar 50 nm) obtained with the sample NP-F/COOH(50), and **d** the histogram of the corresponding size distribution (r_{cs} = 8.4 ± 1.1 nm, as obtained from > 400 NPs). Red outlines correspond to the NPs analyzed in these images.

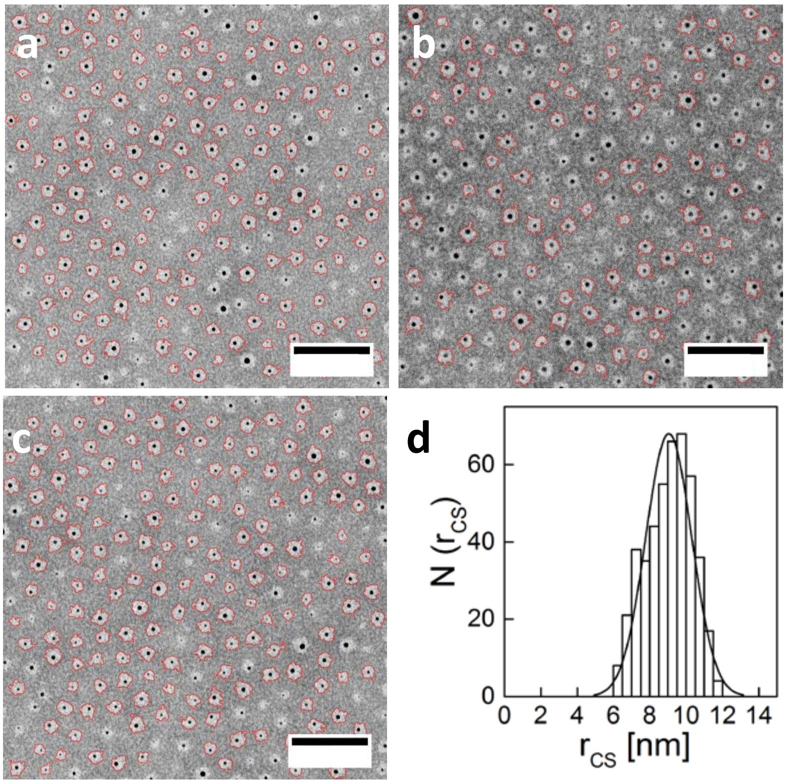

Supplementary Figure 46: **a-c** Selected low magnification negative staining TEM micrographs (scale bar, 100 nm) obtained with the sample NP-F, and the histogram of the corresponding size distribution (r_{cs} = 9.0 ± 1.2 nm, as obtained from > 400 NPs). Red outlines correspond to the NPs analyzed in these images.

Ultraviolet – Visible absorption spectra measurement (UV-Vis)

UV-Vis absorption spectra were recorded in water as described before, and the absorption A(λ) was normalized at λ = 450 nm. The maximum absorption for NP-F/COOH (50) was at λ = 511 nm and for NP-F was at λ = 509 nm (Supplementary Figure 47).

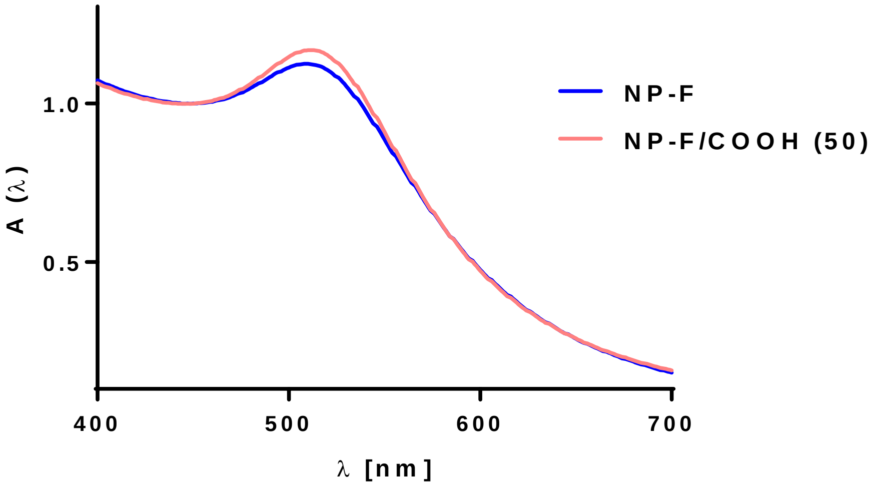

Supplementary Figure 47: UV-Vis absorption spectra of NP-F/COOH(50) and NP-F measured in water. A(λ) is normalized at λ = 450 nm.

ζ potential measurement

The zeta potential values of NPs were recorded in water as described before. The distributions N(ζ) of obtained zeta potentials are depicted in Supplementary Figure 48 along with the obtained values.

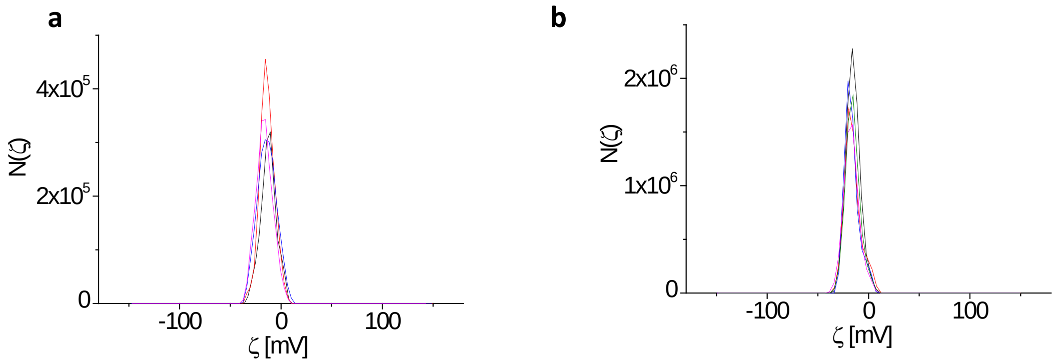

c

NP type	ζ [mV]
NP-F/COOH (50)	-16.0 ± 0.7
NP-F	-13.1 ± 0.7

Supplementary Figure 48: Zeta potential distributions N(ζ) recorded in water for **a** NP-F/COOH(50) and **b** NP-F. **c** Table with the average zeta potential values for each sample with the standard deviation (SD) corresponding to at least 3 measurements.

Size calculation from ^{19}F-diffusion measurements in the presence of proteins

Firstly, we incubated NP-F, NP-F/COOH, NP-F/COOH(50), and NP-F/NH_2 with HSA (1.5 mM) or fibrinogen (FIB, 1 µM) in PBS and measured the D value for each NP with and without the presence of proteins by ^{19}F diffusion NMR. Fibrinogen was purchased from Sigma (FIB, #F3879). As before, from the D values we obtained r_h from the Einstein-Stokes relation (Supplementary Table 22).

Supplementary Table 22: Values of hydrodynamic radii r_h as obtained from experimentally measured diffusion constants D for NPs incubated with HSA (1.5 mM) or FIB (1 µM), and the corresponding standard deviation (SD). The n value refers to the number of independent measurements performed for each experiment.

NP	Protein	c_{Prot}	D [10^{-11} m^2/s]	r_h [nm]	n
NP-F	None	0	2.81 ± 0.10	8.74 ± 0.33	5
	HSA	1.5 mM	2.05 ± 0.09	8.45 ± 0.36	5
	FIB	1 µM	3.51 ± 0.13	6.94 ± 0.26	2
NP-F/COOH	None	0	2.59 ± 0.04	9.45 ± 0.15	6
	HSA	1.5	1.87 ± 0.10	9.19 ± 0.49	2
	FIB	1 µM	2.51 ± 0.03	9.58 ± 0.10	2
NP-F/NH_2	None	0	2.79 ± 0.08	8.79 ± 0.25	3
	HSA	1.5	2.38 ± 0.24	7.32 ± 0.78	2
	FIB	1 µM	2.99 ± 0.18	8.14 ± 0.48	
NP-F/COOH(50)	None	0	2.62 ± 0.04	9.34 ± 0.14	3
	HSA	1.5	1.94 ± 0.04	8.95 ± 0.17	3

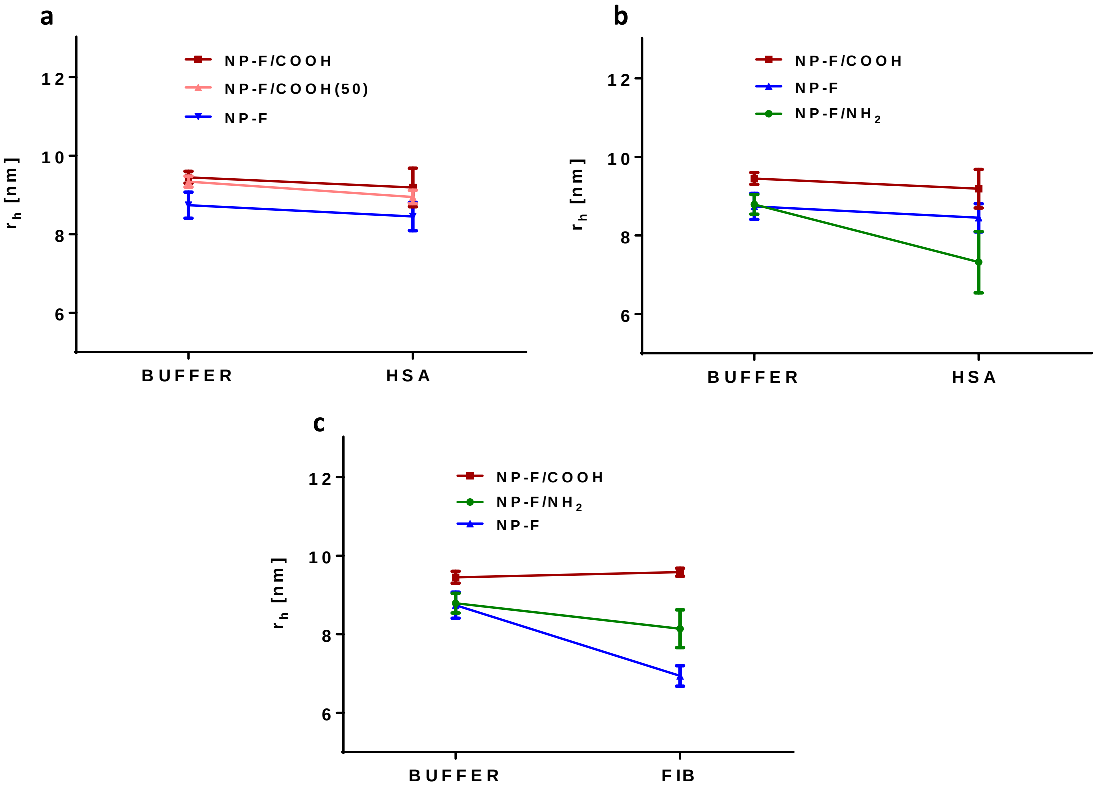

Supplementary Figure 49: Hydrodynamic radii r_h ± standard deviation for NP-F/COOH, NP-F/COOH(50), NP-F/NH_2, and NP-F in buffer (PBS), **a**-**b** in the presence of HSA (1.5 mM) or **c** FIB (1 µM).

On the one hand, for the case of different fluorine loadings (100-50%) in the presence of high amount of HSA, we did not observe a different behaviour due to an increased amount of fluorine, and the size of all NPs in Supplementary Figure 49**a** remained mostly the same in buffer or in the presence of HSA.

On the other hand, we observed different behaviour when changing proteins and non-fluorinated ligand (Supplementary Figure 49**b-c**). Given that the isoelectric points (pI) for HSA

and FIB are rather similar and well below 7.4 (pI = 5.7-5.9 for HSA; pI = 5.1-6.3 for FIB) we can assume that in PBS both proteins are more or less negatively charged. Interestingly, when NPs were placed in the presence of HSA, the most affected NP was NP-F/NH_2 (positively charged), whereas NP-F/COOH and NP-F/COOH(50) (negatively charged) remained mostly unchanged (Supplementary Figure 49**b**). However, in the case of FIB we observed another trend, and the most affected NP was the most hydrophobic one, NP-F, and the second most affected was the second most hydrophobic, NP-F/NH_2, according to IFT measurements (Supplementary Figure 50 and Supplementary Figure 15). FIB is known to be more hydrophobic than HSA and may interact preferably through hydrophobic interactions. HSA interactions, on the contrary, may be mainly driven by electrostatic interactions between oppositely charged molecules. In any case, what we observe in all cases is shrinkage of the hydrodynamic size.

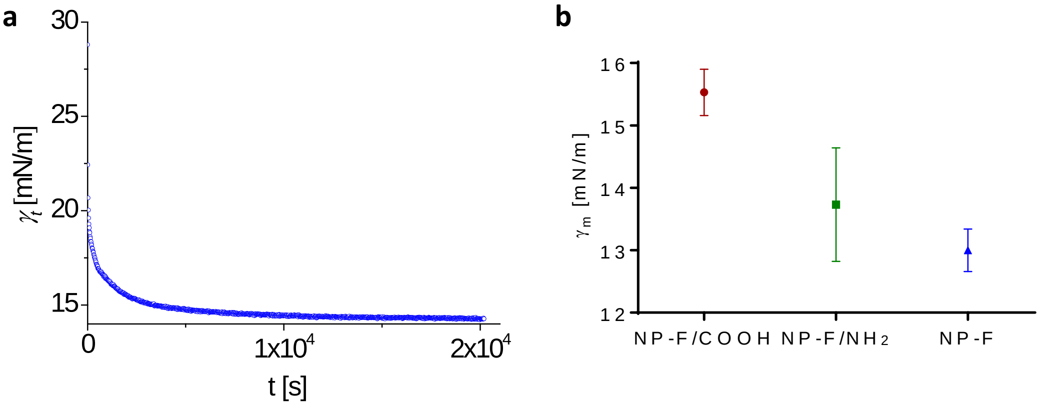

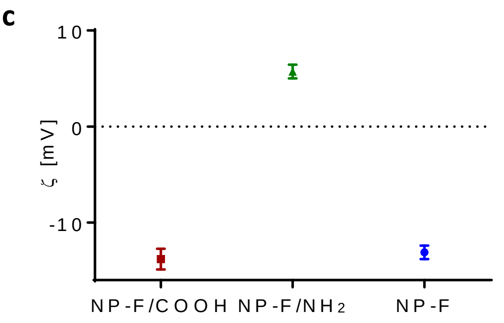

d

NP type	γ_m [mN/m]	ζ [mV]
NP-F/COOH	15.53 ± 0.37	-13.8 ± 1.1
NP-F/NH_2	13.73 ± 0.91	5.7 ± 0.7
NP-F	13.00 ± 0.34	-13.1 ± 0.7

Supplementary Figure 50: **a** Selected dynamic surface tension (γ_t) plot for NP-F. **b** Graphical representation of IFT values obtained for NP-F/COOH, NP-F/NH_2 and NP-F for comparison purposes. **c** Graphical representation of zeta potential values obtained for NP-F/COOH, NP-F/NH_2 and NP-F for comparison purposes. **d** Table with the average γ_m and ζ values for each type of NP with the standard deviation (SD) corresponding to at least 3 measurements.

Diffusion constant (D) measurement by ^{19}F diffusion NMR of NPs with ligands other than PEG

By using PEGylated ligands we ensure the water solubility of the resulting NPs, since the presence of a big amount of ethylene groups seems to counterbalance the high hydrophobicity of fluorine atoms. In addition, the flexibility of PEG warrants for the high mobility of the fluorinated head as reflected in T_2 values (Supplementary Figure 18). In any case, other non-

PEGylated systems have also been measured for the sake of completeness. As previously described by us[17], the following ligands (HSC_{11}TEG-GlcF; HSC_{11}TEG-F and HSC_{11}HEG-OH) and corresponding NPs (NP-GlcF/OH and NP-TEGF) were prepared and characterized. NP-GlcF/OH is water dispersible but NP-TEGF is not, hence the latter was coated with PMA functionalized with Dodecylamine (PMA-DDA), as reported before[17,2], to afford water dispersible NP-TEGF@PMA. All the ligands and NPs structures are depicted in Supplementary Figure 51.

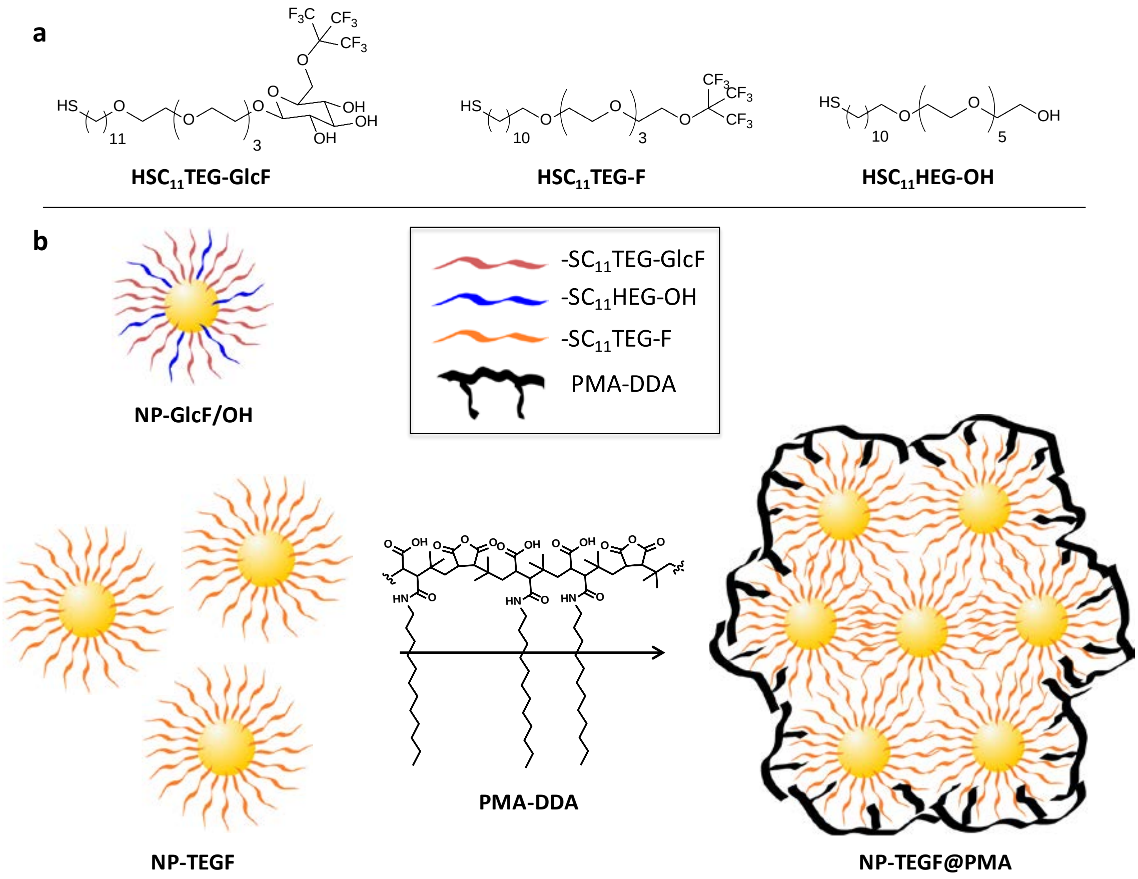

Supplementary Figure 51: Schematic representation of **a** non-PEGylated ligands and **b** water dispersible fluorinated NPs obtained with them.

TEM analysis of NP-GlcF/OH and NP-TEGF afforded r_c values of 0.9 ± 0.6 nm and 1.1 ± 0.2 nm, respectively. However, when NP-TEGF was coated with PMA, groups of NPs were coated together, most likely due to F-F non covalent interactions. Those groups of NPs had a mean radius of 12.6 ± 3.8 nm (Supplementary Figure 52).

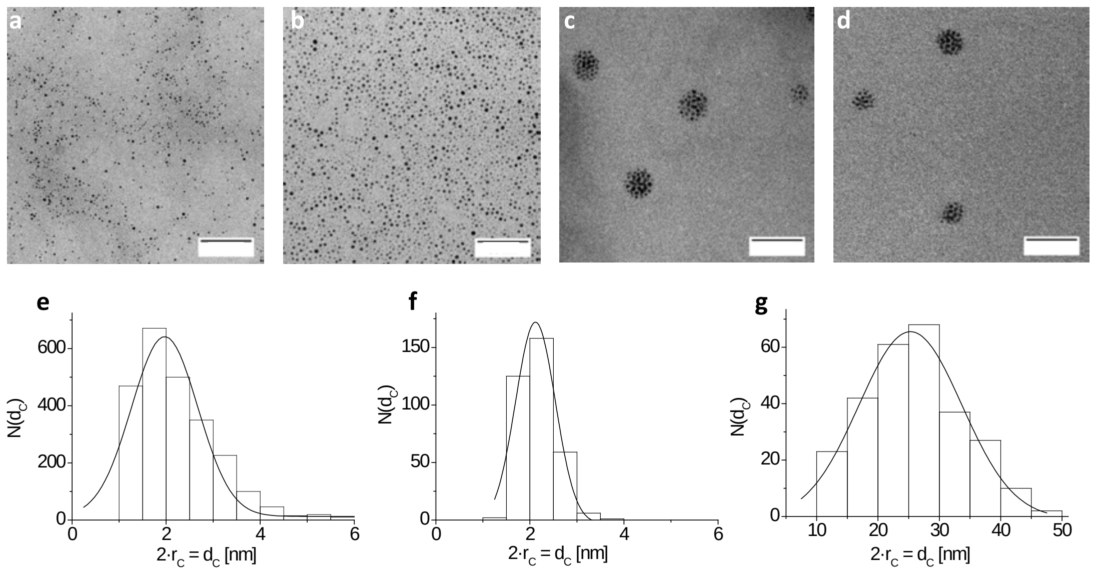

Supplementary Figure 52: Selected TEM micrographs (**a** scale bar, 100 nm; **b-d** scale bar, 50 nm) and histograms for size distribution of the Au NP radii without organic shell (r_C) for NP-GlcF/OH **a**, **e**; NP-TEGF **b, f** and NP-TEGF@PMA **c-d, g**. Histograms were obtained after analysis of at least 300 NPs.

Regarding the ^{19}F-NMR signal of NP-GlcF/OH, the presence of 2 peaks accounts for 2 populations of fluorine due to the surrounding environment. For the case of NP-TEGF@PMA a considerable broadening was observed due to PMA entrapment and possibly a broad distribution of environments around fluorine atoms (Supplementary Figure 53**a**). UV-Vis absorption spectra were also measured and showed the lack of plasmon band typical of very small gold NPs, such as these ones (Supplementary Figure 53**b**). The synthesis and characterization of these NPs has already been described by us[17].

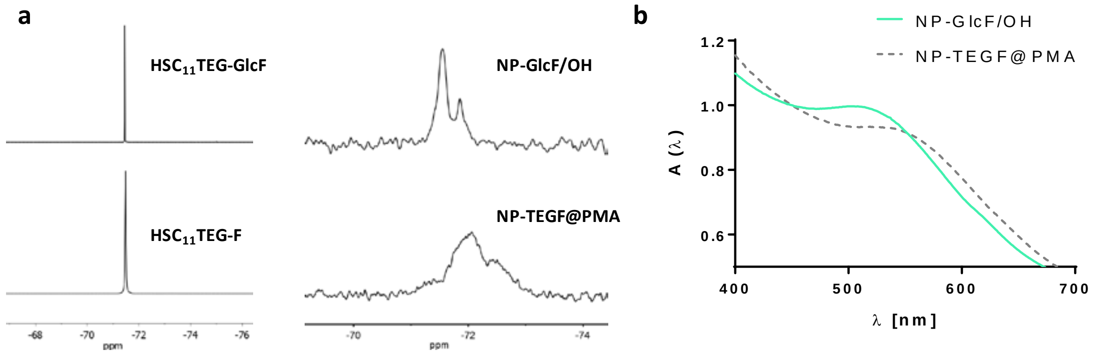

Supplementary Figure 53: ^{19}F-NMR spectra of **a** ligands HSC_{11}TEG-GlcF and HSC_{11}TEG-F (left) and NP-GlcF/OH and NP-TEGF@PMA (centre) and **b** their UV-Vis absorption spectra measured in water. A(λ) is normalized at λ = 450 nm.

The T_2 values of these NPs were calculated as described before and were 76 ± 2 ms and 71 ± 6 ms for NP-GlcF/OH and NP-TEGF@PMA, respectively. The diffusion constant of these NPs was measured by ^{19}F-NMR diffusion in water and used to calculate the hydrodynamic radius following the Einstein-Stokes relation following the protocols described before.

Supplementary Table 23: Values of hydrodynamic radii r_h as obtained from experimentally measured diffusion constants *D* for NPs in water and the corresponding standard deviation (SD) after two independent measurements.

NP type	D [10^{-11} m^2/s]	r_h [nm]
NP-GlcF/OH	8.19 ± 0.23	3.00 ± 0.08
NP-TEGF@PMA	1.97 ± 0.19	12.56 ± 1.33

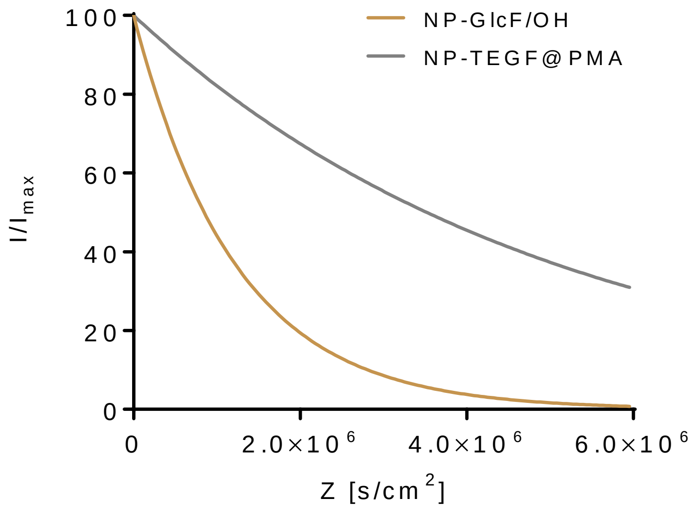

Supplementary Figure 54: Plot of normalized fitted mean mono exponential decay curves for NP-GlcF/OH and NP-TEGF@PMA.

Diffusion constant (D) measurement by ^{19}F diffusion NMR of NPs of larger size

We prepared PMA coated NPs as those described before Gold NP synthesis section, but using a PMA monomer to surface ratio of 0.16 mmol/m^2 to achieve only partial reaction of the amino groups of the starting NP-F/NH_2. The partial coating obtained led to dimmer formation in solution due to electrostatic interactions. From now on these dimmers will be called [NP-F/NH_2@PMA]*2.

*Synthesis of [NP-F/NH_2@PMA]*2.*

In a typical procedure, NP-F/NH_2 (1.42 nmol) was dried under high vacuum to remove all water molecules and resuspended in 6.48 mL of CH_2Cl_2 in a glass vial under stirring at room temperature (r.t.). PMA solution in tetrahydrofuran (9.2 mL, 17.5 mM in monomers) was added onto the NPs, followed by 4-(dimethylamino)pyridine (DMAP) (95 µL, 1.5 M in CH_2Cl_2) as a catalyst for the amide bond formation through the reaction between cyclic anhydrides on PMA and amine moieties on the NPs (Supplementary Figure 7**a**). The vial was capped and the mixture was allowed to stir overnight at r.t. Next morning, the NPs were extensively washed with THF by centrifugation to remove unbound PMA, which remained dissolved in THF in the supernatant (above the NP pellet) and was discarded (4 times at 2 x 10^3 g during 4 min). Subsequently, the

NPs obtained as a pellet after centrifugation were treated with 0.5 mL of NaOH (0.1 M) followed by 1 mL of NaOH (5 M) to transform all anhydride moieties present in PMA into the corresponding carboxylates[2]. The so-obtained NPs were further centrifuged twice at 125 x 10^3 g for 45 min using PBS (phosphate buffered saline solution, pH=7.4) as the washing solution. The supernatant was discarded and the so-obtained pellet of NPs was resuspended and centrifuged once at 10^4 g for 4 min to remove bigger aggregates. The supernatant with [NP-F/NH_2@PMA]*2 dissolved in it was kept and further characterized.

For the particular batch and example presented herein, the PMA/surface ratio was calculated as follows:

$N_{NP} = 1.42\cdot10^{-10}$ mol x $6.023\cdot10^{23}$ mol^{-1} = $8.55\cdot10^{14}$ NPs per reaction

From ^{19}F diffusion measurements the hydrodynamic radius for this particular batch is known as r_h = 9.58 nm, hence:

$A_{h,tot} = 4 \times \pi \times (9.58\cdot10^{-9}\ m)^2 \times 8.55\cdot10^{14} = 9.86\cdot10^{-1}\ m^2$ total surface of all NPs per reaction

The total amount of PMA monomers added to the reactions was:

n_P = 9.2 mL x $1.75\cdot10^{-2}$ M = $1.61\cdot10^{-1}$ mmol of PMA monomers per reaction

Hence, the used ratio between PMA monomers and NP surface was:

$n_p/A_{h,tot} = 1.61\cdot10^{-1}$ mmol / $9.86\cdot10^{-1}\ m^2$ = 0.16 mmol/m^2

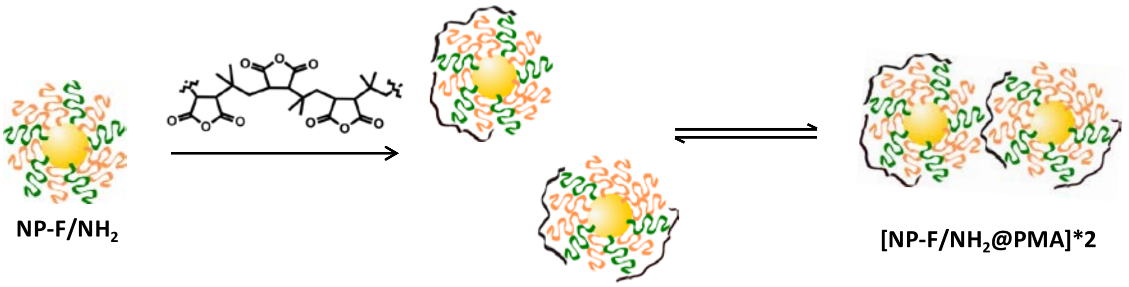

Supplementary Figure 55: Schematic representation of [NP-F/NH_2@PMA]*2.

The so-obtained nanoparticles were analyzed by TEM, negative staining, UV-Vis absorption spectroscopy, ζ-potential measurements, ^{19}F-NMR spectroscopy, T_2 measurements, and IFT calculations, following the protocols and with the equipment already described before. The results are summarized below.

Transmission Electron Microscopy (TEM)

After TEM analysis, a core radius of r_C = 1.9 ± 0.6 nm was obtained for [NP-F/NH_2@PMA]*2 (Supplementary Figure 56). The organic ligand shell does not provide sufficient contrast and thus is not included in the core diameter.

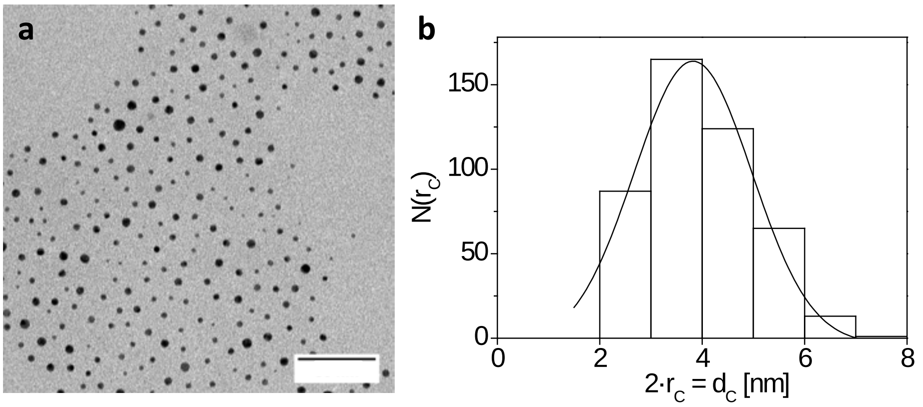

Supplementary Figure 56: **a** TEM micrograph (scale bar, 50 nm) and **b** histogram for size distribution of the Au NP radius without organic shell (r_c) for [NP-F/NH_2@PMA]*2. Histogram was obtained after analysis of at least 500 NPs. The core radius r_c is half the core diameter d_c.

Negative staining

The image analysis of several negative staining TEM micrographs of [NP-F/NH_2@PMA]*2 was done. For morphological characterization of NP-organic shell system, the borders between the organic shells and the stained grid were automatically segmented by ImageJ[4,5] (function Analyzed Particles) (Supplementary Figure 57). The total area limited by the outlines was divided by the total number of NPs analyzed, to give a spherically-idealized mean area occupied by the NP-organic shell system (A_{CS}), from which the mean r_{CS} can be extracted (*i.e.*, $r_{CS}=\sqrt[2]{A_{CS}/\pi}$).

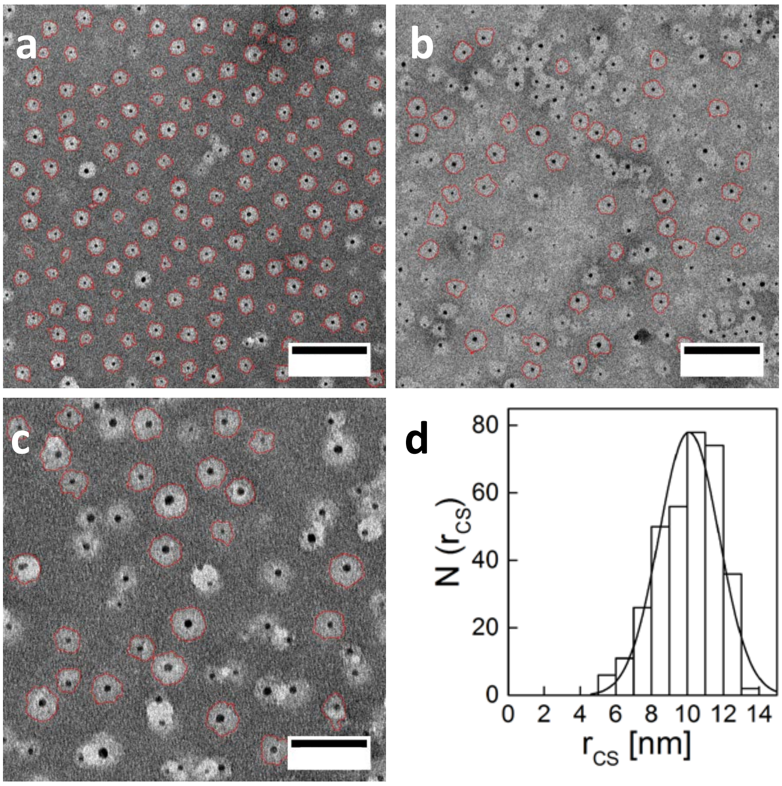

Supplementary Figure 57: **a-c** Selected low magnification negative staining TEM micrographs (**a-b** scale bar, 100 nm; **c** scale bar, 50 nm) obtained with the sample [NP-F/NH_2@PMA]*2, and **b** the histogram of the corresponding size distribution (r_{cs} = 10.0 ± 1.6 nm, as obtained from > 300 NPs). Red outlines correspond to the NPs analyzed in these images.

Ultraviolet – Visible absorption spectra measurement (UV-Vis)

UV-Vis absorption spectra were recorded in water and the absorption A(λ) was normalized at λ = 450 nm. The maximum absorption for [NP-F/NH_2@PMA]*2 was at λ = 513 nm (Supplementary Figure 58)

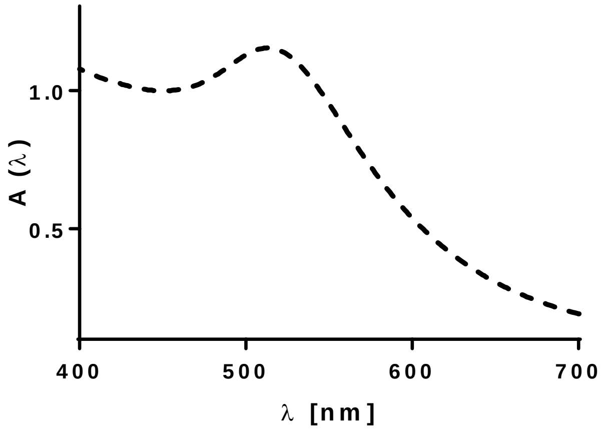

Supplementary Figure 58: UV-Vis absorption spectrum of [NP-F/NH_2@PMA]*2 measured in water. A(λ) is normalized at λ = 450 nm.

ζ potential measurement

The zeta potential value of [NP-F/NH_2@PMA]*2 was recorded in water at a NP concentration of c_{NP} ≈ 20 nM. The distribution N(ζ) of obtained zeta potential is depicted in Supplementary Figure 59 along with the obtained mean value. For the sake of comparison, zeta potential values for NP-F/NH_2 and NP-F/NH_2@PMA were also depicted and they varied accordingly with the degree of PMA coating from neutral or slightly positive in the absence of PMA (5.7 ± 0.7 mV for NP-F/NH_2) to negative due to the presence of carboxylic groups (-10.7 ± 0.8 mV and -36.2 ± 1.2 mV for [NP-F/NH_2@PMA]*2 and NP-F/NH_2@PMA, respectively).

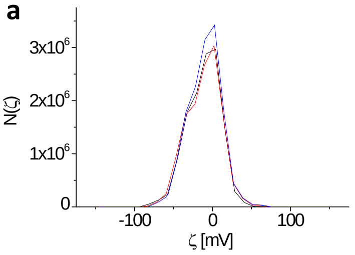

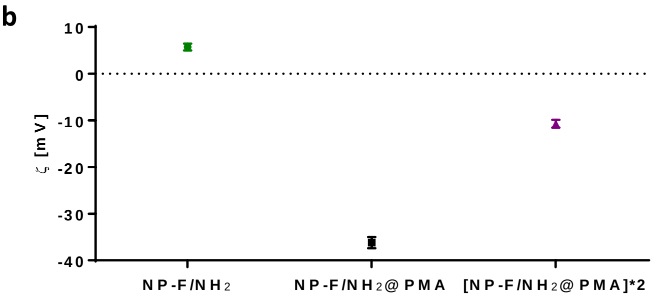

c

NP type	ζ [mV]
NP-F/NH_2	5.7 ± 0.7
NP-F/NH_2@PMA	-36.2 ± 1.2
[NP-F/NH_2@PMA]*2	-10.7 ± 0.8

Supplementary Figure 59: **a** Zeta potential distribution N(ζ) for [NP-F/NH_2@PMA]*2 recorded in water. **b** Graphical representation of zeta potential values obtained for NP-F/NH_2, NP-F/NH_2@PMA and [NP-F/NH_2@PMA]*2. **c** Table with the average zeta potential values for each sample with the standard deviation (SD) corresponding to at least 3 measurements.

Interfacial Tension (IFT) measurement

Dynamic interfacial tension (IFT) of [NP-F/NH_2@PMA]*2 was evaluated by pendant drop tensiometry and depicted in Supplementary Figure 60. For the sake of comparison, IFT values for NP-F/NH_2 and NP-F/NH_2@PMA were also depicted and they also varied with the degree of PMA coating from more hydrophobic in the absence of PMA (13.73 ± 0.91 mN/m for NP-F/NH_2) to more hydrophilic due to the increased presence of carboxylic groups from PMA (17.48 ± 0.39 mN/m and 18.37 ± 0.69 mN/m for [NP-F/NH_2@PMA]*2 and NP-F/NH_2@PMA, respectively) (Supplementary Figure 60).

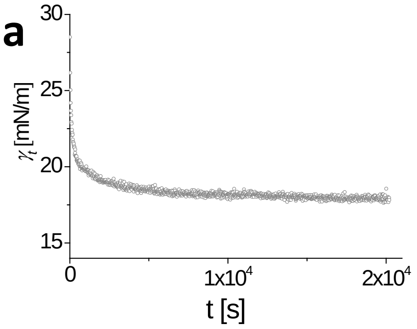

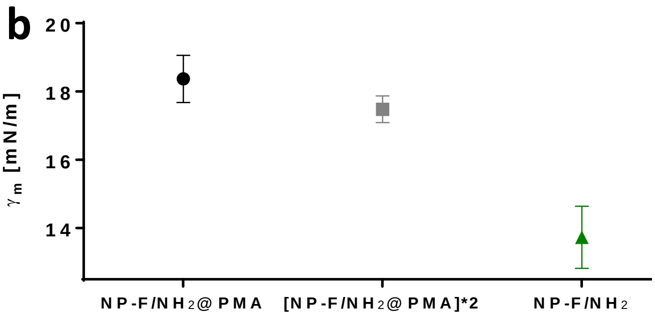

c

NP type	γ_m [mN/m]
NP-F/NH_2	13.73 ± 0.91
NP-F/NH_2@PMA	18.37 ± 0.69
[NP-F/NH_2@PMA]*2	17.48 ± 0.39

Supplementary Figure 60: **a** Selected dynamic surface tension (γ_t) plot for [NP-F/NH_2@PMA]*2, where the interfacial tension decreases with time, approaching an equilibrium value. **b** Graphical representation of IFT values obtained for NP-F/NH_2, NP-F/NH_2@PMA and [NP-F/NH_2@PMA]*2.

c Table with the average γ_m values for each type of NP with the standard deviation (SD) corresponding to 3 measurements.

^{19}F nuclear magnetic resonance (^{19}F-NMR) spectra measurement

^{19}F-NMR spectrum of [NP-F/NH_2@PMA]*2 was measured in a mixture of water and deuterium oxide (H_2O:D_2O 85:15) as before. As expected, a single peak was obtained for all fluorine atoms. The chemical shift (δ) of the fluorine on the NPs was slightly shifted when compared to NP-F/NH_2@PMA (-71.24 ppm vs. -71.34 ppm, respectively), using TFA as the reference. Supplementary Figure 61 shows the comparison between ^{19}F-NMR spectra of NP-F/NH_2@PMA and [NP-F/NH_2@PMA]*2.

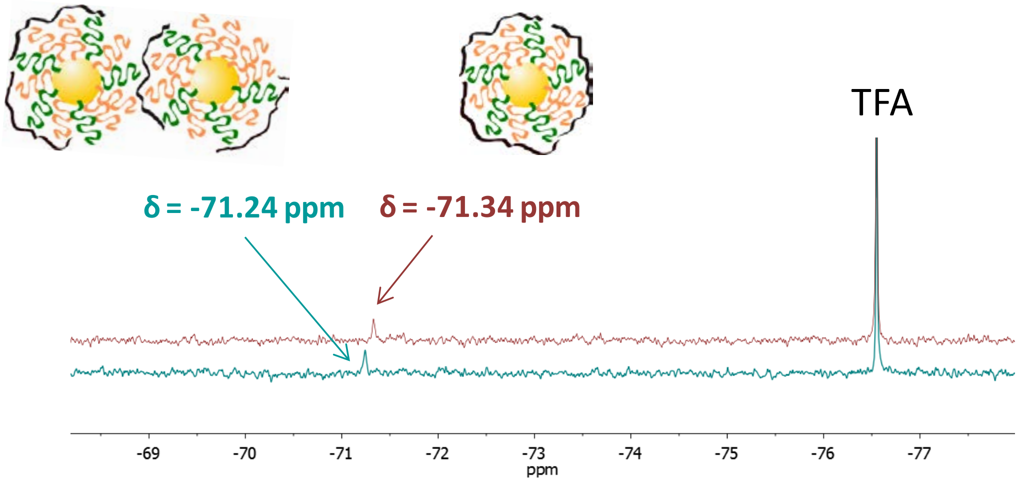

Supplementary Figure 61: ^{19}F-NMR spectra comparison between [NP-F/NH_2@PMA]*2 and NP-F/NH_2@PMA, recorded in water using TFA as the chemical shift reference.

Transverse relaxation time (T_2) measurement of fluorine

The T_2 value for fluorine when bound to [NP-F/NH_2@PMA]*2 was measured in a mixture of water and D_2O and was recorded to be 654 ± 84 ms after two independent measurements. As shown in Supplementary Figure 62, the so-obtained T_2 value was compared to those of NP-F/NH_2 and NP-F/NH_2@PMA and we observe a progressive decrease in fluorine T_2 value as we moved from NP-F/NH_2 to NP-F/NH_2@PMA and [NP-F/NH_2@PMA]*2, which might indicate a greater mobility constrain for fluorine in [NP-F/NH_2@PMA]*2, although the value for the latter is still very high.

a

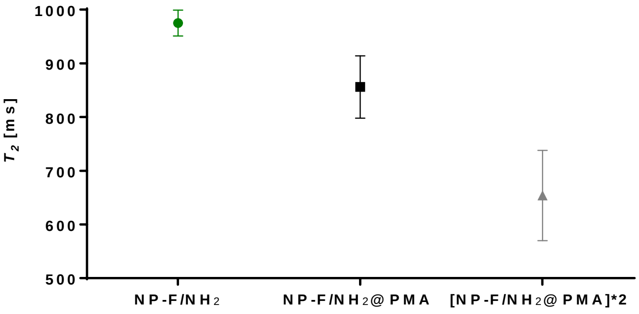

b

NP type	T_2 [ms]
NP-F/NH_2	975 ± 24
NP-F/NH_2@PMA	856 ± 58
[NP-F/NH_2@PMA]*2	654 ± 84

Supplementary Figure 62: **a** Graphical representation of T_2 values obtained for NP-F/NH_2, NP-F/NH_2@PMA and [NP-F/NH_2@PMA]*2. **b** Table with the average T_2 values for each type of NP with the standard deviation (SD) corresponding to 2 measurements.

*Size correlation for TR naturally adsorbed onto [NP-F/NH_2@PMA]*2 by ^{19}F diffusion NMR*

NMR samples of [NP-F/NH_2@PMA]*2 mixed with increasing concentrations of TR in PBS were prepared as described before. Hence, 465 µL of a PBS solution of [NP-F/NH_2@PMA]*2 and TR mixture of 1 µM in NP and with TR concentrations ranging from 0 to 0.3 mM were introduced in NMR tubes as described before, and the ^{19}F diffusion NMR spectra were recorded. ^{19}F diffusion NMR protocol had to be adjusted for bigger NPs by only modifying D20 (gradient length) and P30 (diffusion delay/2) values to 0.6 s and 2.0 ms, respectively. The work-flow shown in Supplementary Figure 22 was followed for each single measurement, and the mean *D* values for each TR concentration together with the corresponding standard deviations (SD) are shown in Supplementary Table 24. The so-obtained fitted decay curves are shown in Supplementary Figure 63. Subsequently, r_h was calculated by introducing the value for the diffusions constants *D* in the Einstein-Stokes relation (*cf*. Supplementary Equation 5). The viscosity of the TR-water mixture $\eta_{TR/H2O}$ was calculated from the viscosity of water η_{H2O} and the additional increase in viscosity due to the presence of TR. The following parameters were used for the calculations:

T = 298 K

k_B = 1.38065 x 10^{-23} $m^2 kg \cdot s^{-2} K^{-1}$

$\eta_{TR/H2O} = \eta_{H2O} \times (1 + c_{TR} \times \eta_{TR})$; η_{TR} = 4.4 $cm^3 g^{-1}$ = viscosity of TR

Supplementary Table 24: Diffusion constants *D* as experimentally obtained from diffusion spectra analysis by fitting the NMR signal intensity to an exponential decay for [NP-F/NH_2@PMA]*2 mixed with TR. Hydrodynamic radii r_h obtained from the experimentally measured diffusion constants *D* for NPs mixed with increasing amounts of TR, and the corresponding standard deviation (SD). The *n* value refers to the number of independent measurements performed for each TR concentration.

Sample	c_{TR} [mM]	D [10^{-11} m^2/s]	r_h [nm]	*n*
NP*2_TR_0_ads	0	1.22 ± 0.06	20.20 ± 1.03	2
NP*2_TR_1_ads	0.0002	1.20 ± 0.03	20.51 ± 0.57	2
NP*2_TR_2_ads	0.0005	1.17 ± 0.02	20.93 ± 0.50	2
NP*2_TR_3_ads	0.001	1.15 ± 0.05	21.43 ± 0.94	3
NP*2_TR_4_ads	0.003	1.11 ± 0.03	22.17 ± 0.50	3
NP*2_TR_5_ads	0.006	1.07 ± 0.02	22.77 ± 0.41	3
NP*2_TR_6_ads	0.01	1.04 ± 0.03	23.42 ± 0.69	3
NP*2_TR_7_ads	0.05	0.99 ± 0.06	24.24 ± 1.33	3
NP*2_TR_8_ads	0.3	0.92 ± 0.03	24.17 ± 0.84	2

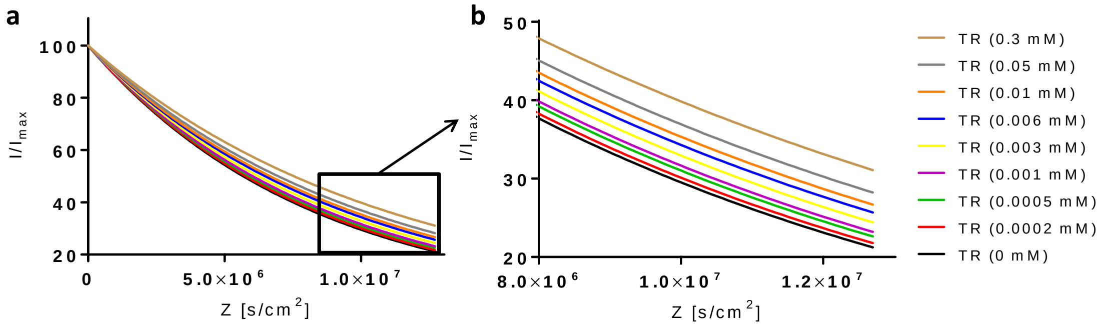

Supplementary Figure 63: **a** Plot of normalized fitted mean mono exponential decay curves for increasing concentration of TR in the presence of [NP-F/NH_2@PMA]*2. **b** Zoomed in area of the same plot as in (**a** .

Radii of samples of TR naturally adsorbed onto [NP-F/NH_2@PMA]*2, which were obtained by diffusion experiments present a sigmoidal distribution, are shown in Supplementary Figure 64, which were fitted with a Hill model (OriginLab), *cf.* fitting parameters in Supplementary Table 25, as follows [15,16]:

$$r_h(N_{TR}) = r_h(0)\sqrt[3]{1+\frac{V_{TR}}{\frac{4\pi}{3}r_h^3(0)}\cdot N_{TR}}$$ Supplementary Equation 9

Where the number of proteins adsorbed per NP N_{TR} is given by the Hill equation,

$$N_{TR} = \frac{N_{max}}{1+\left(\frac{K_d^{'}}{c_P}\right)^n}$$ Supplementary Equation 10

Supplementary Table 25: Compilation of fit parameters as obtained from the diffusion data shown in Supplementary Figure 64. $r_h(0)$ is the hydrodynamic diameter of the NPs without TR added, as obtained from fitting the measured $r_h(c)$ data points with the Hill model. The fit also returns the parameters K'_d, N_{max}, Δr_h and n, which are the apparent dissociation coefficient, maximum number of proteins bound per NP, corona thickness, *i.e.*, the difference in radius of NPs saturated with proteins and NPs without proteins, and the Hill coefficient, respectively.

fit parameters	Values
$r_h(0)$ [nm]	20.23 ± 0.13
Δr_h [nm]	4.12 ± 0.32
$K_d^{'}$ [μM]	3.8 ± 0.6
N_{max}	216.6 ± 11.6
n	0.9 ± 0.1

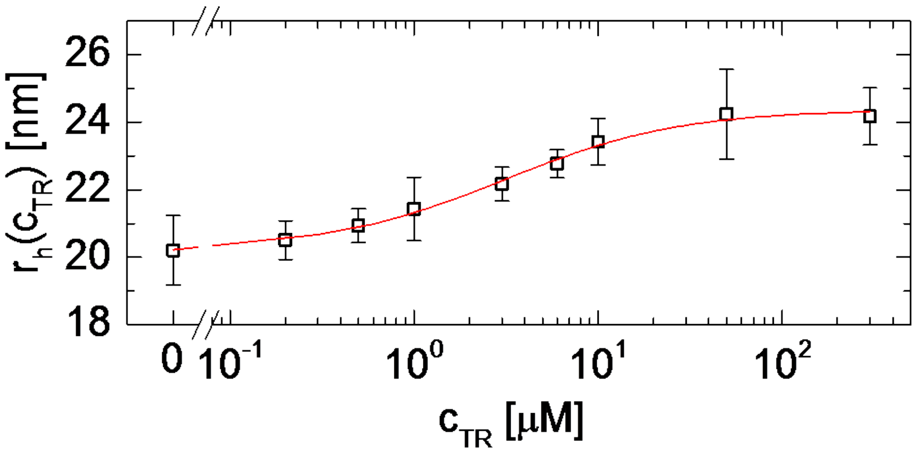

Supplementary Figure 64: Hydrodynamic radius r_h ± standard deviation as measured for the different NP-protein complexes upon the presence of different concentrations c_{TR} of TR in PBS, and the corresponding fits based on the Hill model. The fit parameters are shown in Supplementary Table 25.

*Size correlation for TR naturally adsorbed onto [NP-F/NH$_2$@PMA]*2 by DLS*

Samples of natural adsorption of TR onto [NP-F/NH_2@PMA]*2 were analyzed by dynamic light scattering (DLS) at 173° in water using a Nano ZS (Malvern, Worcesterhire, UK) equipped with a 633 nm laser, with the following results, as shown in Supplementary Figure 65. Raw data of the mean r_h for the DLS distributions in number ($r_{h(N)}$), volume ($r_{h(V)}$), and intensity ($r_{h(I)}$) are shown in Supplementary Table 25. The actual DLS distributions are shown in Supplementary Figure 66 and 67.

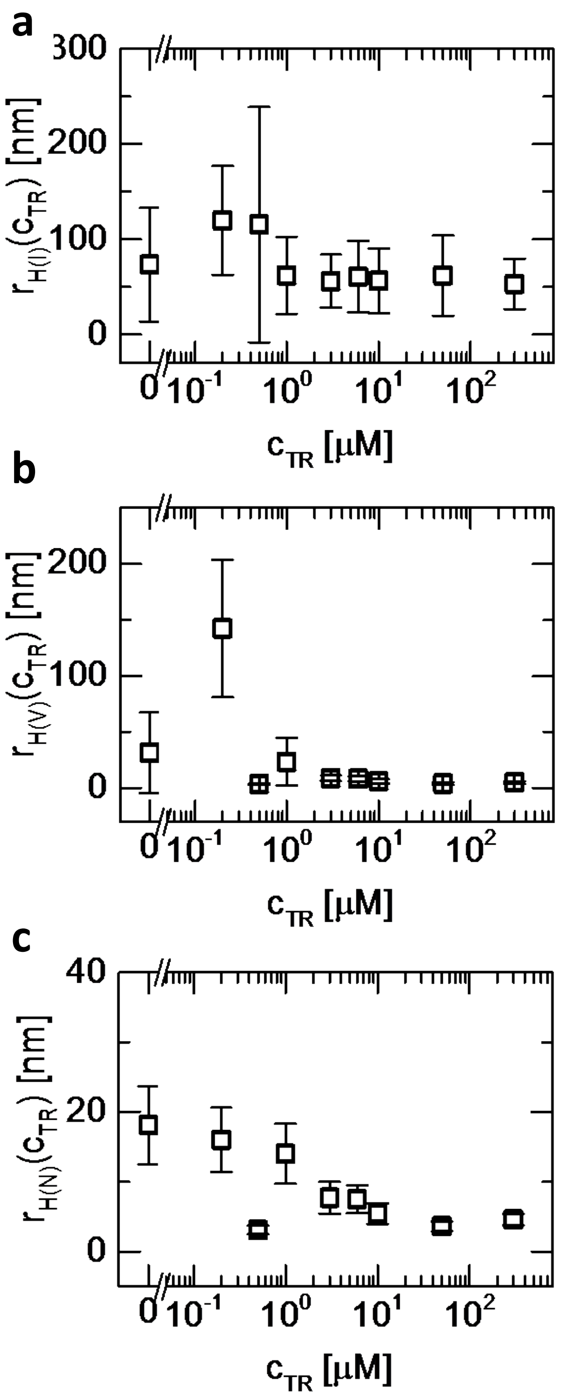

Supplementary Figure 65: Mean hydrodynamic radii ± standard deviation derived from the DLS distributions in **a** intensity, **b** volume, and **c** number for TR naturally absorbed to [NP-F/NH_2@PMA]*2, by using increasing concentrations of TR.

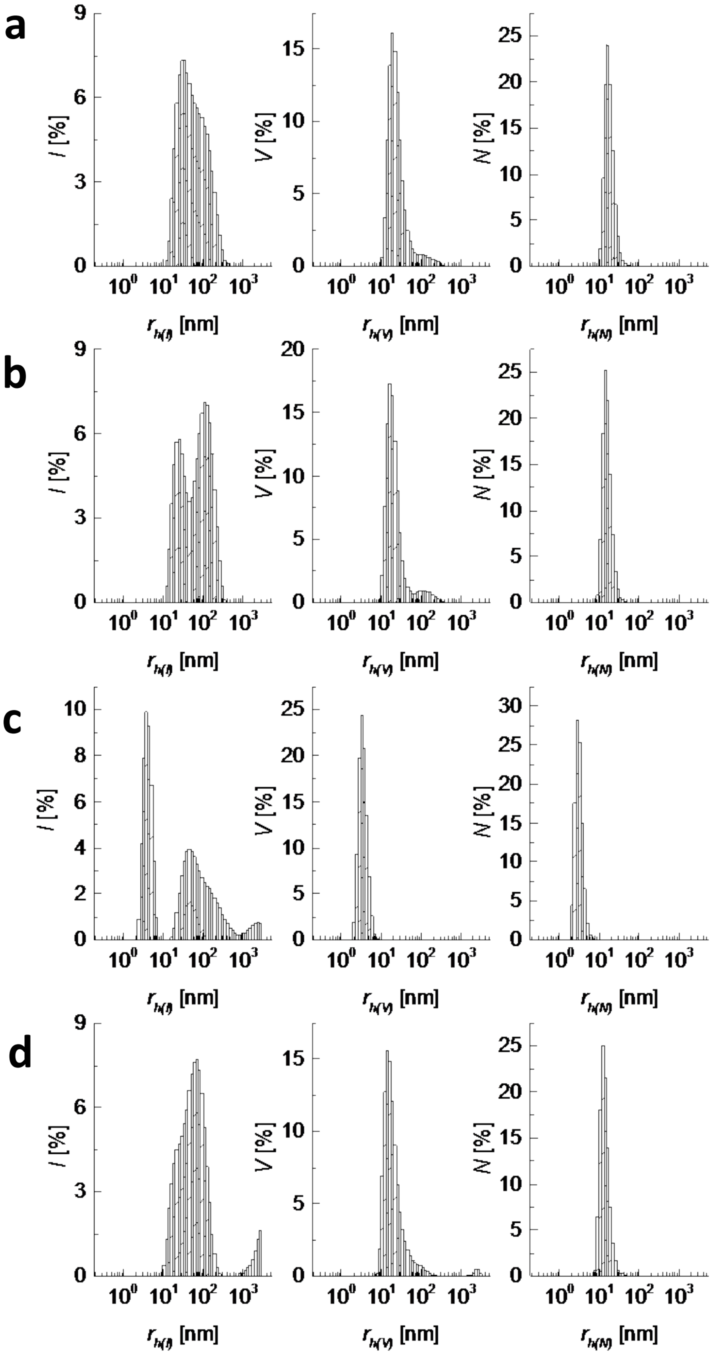

Supplementary Figure 66: DLS distributions of samples exposed to different concentrations of TR, *i.e.*, **a** 0 mM, **b** 0.0002 mM, **c** 0.0005 mM and **d** 0.001 mM.

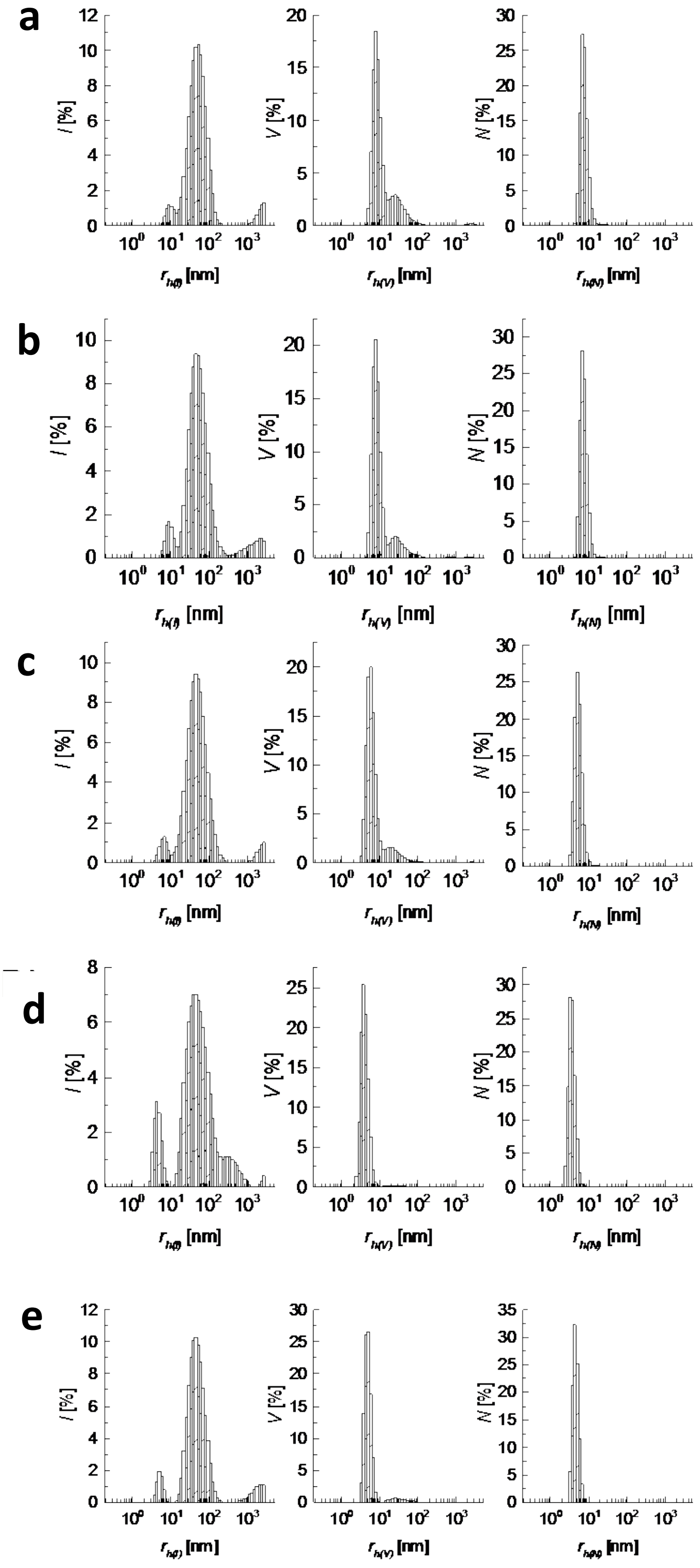

Supplementary Figure 67: DLS distributions of samples exposed to different concentrations of TR, *i.e.*, **a** 0.003 mM, **b** 0.006 mM, **c** 0.01 mM, **d** 0.05 mM and **e** 0.3 mM.

Supplementary Table 26: Mean hydrodynamic radii from the intensity $r_{h(I)}$, volume $r_{h(V)}$, and number $r_{h(N)}$ distributions (mean value ± SD) of the NP-F/NH_2@PMA]*2 exposed at increasing concentrations of TR. Notice the mean values from the most populated species were used, as in most cases two peaks appeared (*i.e.*, from the NPs and the free TR).

Sample	c_{TR} [mM]	$r_{h(I)}$ [nm]	$r_{h(V)}$ [nm]	$r_{h(N)}$ [nm]	PDI
NP*2_TR_0_ads	0	73.1 ± 59.7	31.6 ± 35.8	18.1 ± 5.6	0.28
NP*2_TR_1_ads	0.0002	119.6 ± 57.2	142.2 ± 61.2	16.0 ± 4.6	0.37
NP*2_TR_2_ads	0.0005	115.2 ± 123.7	3.5 ± 0.8	3.1 ± 0.6	0.72
NP*2_TR_3_ads	0.001	61.9 ± 40.4	23.4 ± 21.2	14.0 ± 4.3	0.43
NP*2_TR_4_ads	0.003	55.8 ± 27.8	8.8 ± 2.4	7.7 ± 2.3	0.32
NP*2_TR_5_ads	0.006	60.5 ± 37.4	8.3 ± 2.1	7.5 ± 2.0	0.40
NP*2_TR_6_ads	0.01	56.2 ± 33.9	6.2 ± 1.9	5.4 ± 1.5	0.41
NP*2_TR_7_ads	0.05	61.7± 42.3	4.0 ± 0.9	3.6± 0.7	0.57
NP*2_TR_8_ads	0.3	52.8 ± 26.5	4.9 ± 0.9	4.6± 0.8	0.41

At this point it is also worth to discuss what the size-limit of diffusion measurements would be theoretically. Indeed, we have estimated the smallest diffusion constant which would correspond to the biggest NP system that we could measure taking into account the used scanning conditions, *i.e.* the NMR scanner being used, and assuming high enough signal intensity to detect the sample. Under these circumstances, the signal attenuation under the influence of field gradient pulses in a stimulated echo using bipolar gradients follows the equation:

$$I = \exp[-\gamma^2\delta^2 g^2 D(\Delta - \delta/3)] \quad \text{Supplementary Equation 11}$$

Such equation corresponds to a combination of previous Supplementary Equations 3 and 4. The assumption that a reliable measurement of is only possible if the field gradient pulses lead to a signal attenuation of 1/e, with typical maximum values for the field gradient amplitude (g = 53 G/cm), diffusion delay (Δ = 0.6 s) and the gradient length (δ = 6 ms) gives an estimation of the minimal diffusion value of $D = 2.5\ 10^{-12}\ m^2s^{-1}$. If we introduce this value in the Einstein Stokes relation, assuming dispersion of the NP in water at 25 °C we obtain a r_h value of approximately 100 nm as the maximum theoretical size we could measure in our conditions. Of course, this value would only be possible if long enough T_2 values for fluorine are maintained and detectable fluorine concentration is achieved. These two factors are highly dependent on the design of the fluorinated nanomaterial. Hence a realistic upper limit for diffusion cannot be estimated in a reliable manner, as it is anticipated that T_2 values and sample concentration will decrease as the size of the nanomaterial increases, leading to a maximum measurable value smaller than 100 nm. In any case, larger radii would also conceptually not help for the determination of the protein corona. In case of a 5 nm protein layer around a NP with 5 nm and 100 nm hydrodynamic diameter, the effective NP diameter with the adsorbed protein layer would be different by 5 nm / 5 nm = 100% and 5 nm / 100 nm = 5%, respectively. Protein adsorption as probed by change in effective hydrodynamic radii thus can only be reasonably applied for small NP.

Diffusion constant (D) measurement by ^{19}F diffusion NMR in a mixture

The ^{19}F-NMR chemical shift range is very broad and hence fluorine nuclei can be very sensitive to changes in the surrounding electronic microenvironment. As an example, in Supplementary Figure 61 we observed this feature when comparing ^{19}F-NMR spectra of NP-F/NH_2@PMA with [NP-F/NH_2@PMA]*2 where a difference of 0.1 ppm was observed and it is enough to distinguish them. In ^{19}F diffusion NMR it would in principle be possible to take advantage of this feature to distinguish different species with different chemical shift in ^{19}F-NMR. When the chemical shift is different enough, there would be no signal overlap and analysis of each signal individually would be straightforward. However, when signals are distinguishable between them, but close enough to be overlapped, we would need to de-convolve the different signals to be able to analyse each signal separately. To illustrate this, we have measure ^{19}F diffusion NMR of a mixture of NP-F/COOH and unbound ligand HS-PEG-F (Supplementary Figure 68). First, it would be necessary to split the arrayed spectrum into single spectra to de-convolve and analyse each of them separately following the same methodology as we have described before.

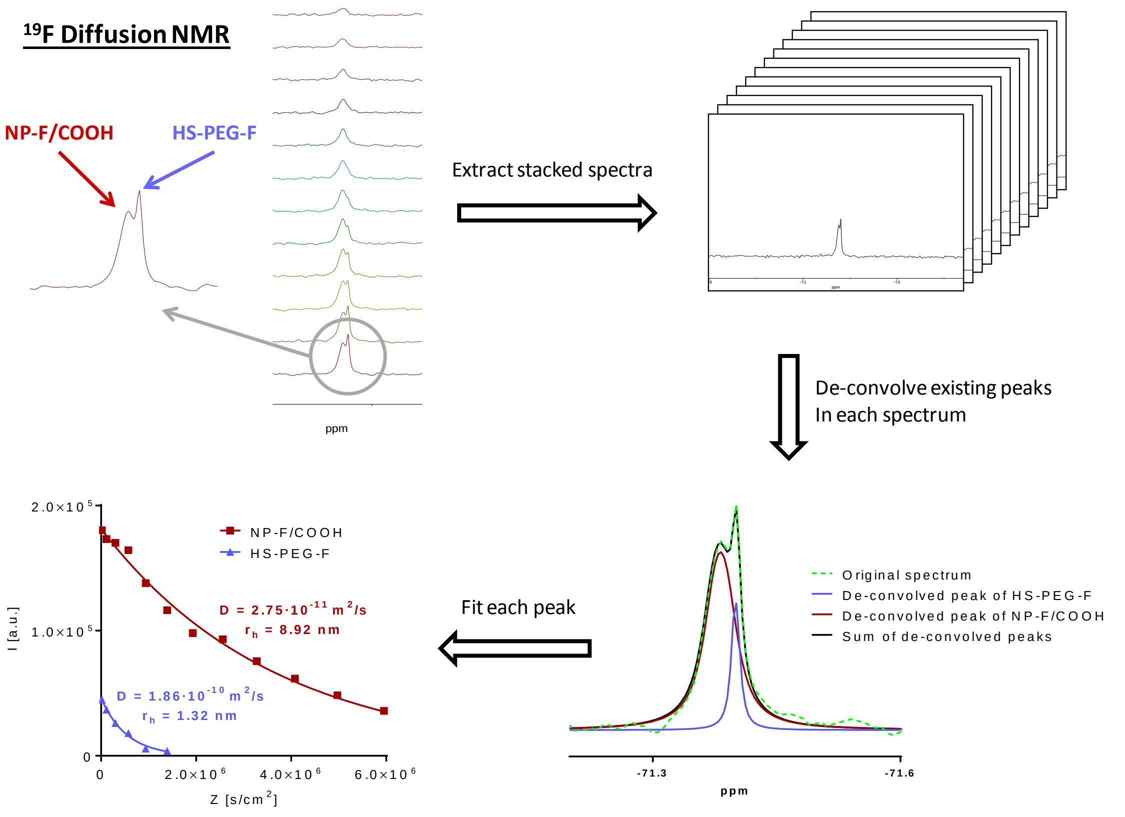

Supplementary Figure 68: Work-flow to obtain the diffusion constant *D* from ^{19}F diffusion NMR experiment in a mixture of NP-F/COOH and HS-PEG-F, which shows the stacked spectra extraction, signal de-convolution for each single spectra and exponential decay fitting to retrieve a value for each diffusion constant *D*.

Following the protocol depicted in Supplementary Figure 68 and for this particular batch we measured *D* values of $2.75 \cdot 10^{-11}$ m^2/s and $1.86 \cdot 10^{-10}$ m^2/s for NP-F/COOH and HS-PEG-F, respectively. With these values and the Einstein-Stokes relation we obtained r_h values of 8.92 nm and 1.32 nm for NP-F/COOH and HS-PEG-F, respectively.

Diffusion constant and size measurements on sedimented cells

In order to emulate presence of NPs above cells, THP-1 cells were incubated with NP-F and then the hydrodynamic radii of the NPs were measured. Cell-NP mixtures placed inside NMR tubes eventually led to precipitation of cells at the bottom of the tube, while non-internalized NPs remained in suspension. Hydrodynamic radii of the NPs in the suspension above the cells were measured. This cell precipitation phenomenom is enhanced inside the scanner by spinning of the NMR tube during measurement as shown in Supplementary Figure 69**a**-**b** for a test measurement only with cells and without NPs.

THP-1 cell culture

Human acute monocytic leukemia THP-1 cells were cultured as suspension cells in 75 cm^2 bottles in Dulbecco's modified Eagle's medium (DMEM) supplemeted with 10% heat-inactivated fetal calf serum (FBS), 2 mM L-glutamine, 100 µg/mL streptomycin, and 100 U/mL penicillin at 37 °C in a humidified environment with 5% CO_2. THP-1 cells were grown for up to 1 month at a density of 2–6 × 10^5 cells/mL, which was maintained by passages performed every 2 – 3 days. For cell counting, the cell suspension was serially diluted 1:10 in PBS and 1:2 in the exclusion dye Trypan Blue solution (0.4 % Trypan Blue in 0.81 % sodium chloride and 0.06 % potassium phosphate). 10 µL of the diluted cell suspension was counted in a haemocytometer chamber under transmitted light in an inverted microscope (DMIL, Leica). Cell viability was determined to be higher than 96 % assessed by trypan blue exclusion method.

NMR sample preparation

The measurements were performed in 5 mm standard NMR tubes with coaxial insert loaded with TFA/D_2O, as described for previous diffusion measurements, in a total volume of at least 465 µL and at 37 °C to mimic physiological conditions. 5 x 10^7 THP-1 cells were centrifuged at 600 g for 5 min to remove excess cell medium, and the cell pellet was resuspended in 465 µL of NP-F solution in water (0.8 µM). The mixture was inmediatelly placed inside an NMR tube and cells were allowed to precipitate leaving a pellet at the bottom of the tube and a transparent NP-F solution on top (Supplementary Figure 69**c**).

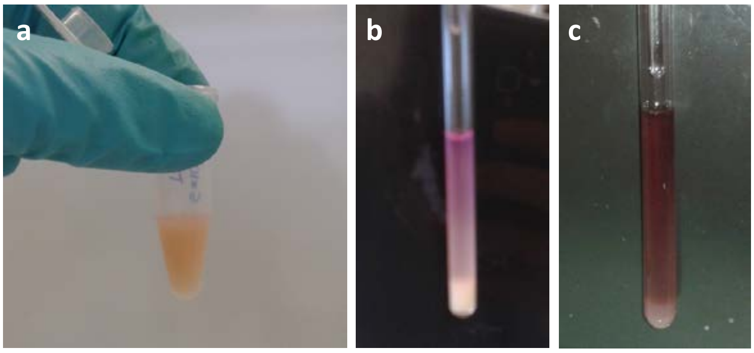

Supplementary Figure 69: **a** Cells suspended in approximately 450 µL of DMEM freshly resuspended before being placed in the NMR tube, and **b** after NMR measurement showing the white cell pellet at the bottom of the tube. **c** Cells suspended in 465 µL of NP-F solution where it is possible to distinguish the cell pellet at the bottom and the NP-F solution on the top.

^{19}F-NMR diffusion measurements of two replicates were performed as described before obtaining a r_h value for NP-F above THP-1 cells of 8.71 ± 0.44 nm, which is similar to that reported for the same NPs in water (r_h = 8.74 ± 0.33 nm, Supplementary Table 22). Hence, data indicate that diffusion measurements of NPs above cells are not affected by the presence of cells (which are not in the zone of detection), and that thus measurements in complex environment are possible.

Diffusion constant and size measurements on cells embedded in agarose

Next, to avoid cell deposition on the bottom of the NMR tubes during the duration of measurements, NPs were incubated with THP-1 cells and embedded in thin agarose gels to measure r_h of NP-F surrounded by cells.

The measurements were performed in 5 mm standard NMR tube without coaxial insert in a total volume of approximately 650 µL and at 37 °C to ensure a degree of fluidity and mimic physiological conditions. The diffusion constant of NP-F when added to THP-1 cells was measured at 0 and 24 hour incubation. The THP-1 cell culture was carried out as described above.

NMR sample preparation

For each NMR measurement, 5 x 10^7 cells were centrifuged at 600 g for 5 min, and the cell pellet was resuspended in 100 µL of DMEM to have the cells in a minimum volume before NP solution, D_2O and agarose solution addition. For the sample named NP-F_gel_cell_0 h, cells were placed on a shaker at 37 °C and 150 rpm, and 240 µL of NP-F (2.2 µM) followed by 150 µL of D_2O were added. Right afterwards, 20 mg of agarose were dissolved in 980 µL of boiling water. 160 µL of this agarose solution were added to the NP-cell mixture and immediately transferred to a NMR tube previously warmed at 37 °C using a warm pipette. The sample was put in the fridge at 4 °C to accelerate gelation and avoid deposition of any of the components before gel formation. For the sample named NP-F_gel_cell_24 h, 100 µL of cell suspension were mixed with 240 µL of NP-F (2.2 µM) at 37 °C and 150 rpm for 24 hours. After that time, 150 µL of D_2O and 160 µL of freshly prepared new agarose solution were added and transferred to an NMR tube in the same manner as described above. Control samples without cells were also prepared by embedding NP-F in agarose gels with and without 100 µL of DMEM.

TFA sample preparation for gel viscosity measurement

For each sample and new agarose gel formation, an equivalent sample with TFA instead of NP-F was prepared to measure the viscosity of the medium and to account for differences in the gel formation from day to day. The final concentration of TFA in all cases was 0.004 % (v/v). All samples prepared are summarized in Supplementary Figure 70 and the viscosity and diffusion results are shown in Supplementary Table 27.

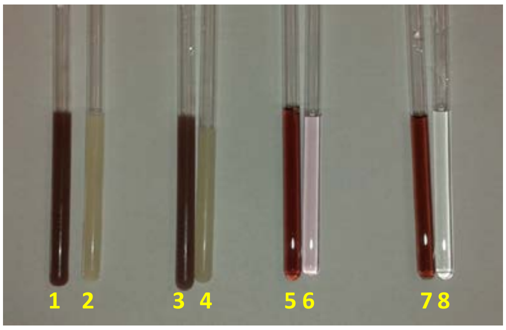

1. NP-F_gel_cell_0h = NP-F + THP-1 + DMEM + Agarose gel; 0 h
2. TFA_gel_cell_0h = TFA + THP-1 + DMEM + Agarose gel

3. NP-F_gel_cell_24h = NP-F + THP-1 + DMEM + Agarose gel; 24 h
4. TFA_gel_cell_24h = TFA + THP-1 + DMEM + Agarose gel

5. NP-F_gel_DMEM = NP-F + DMEM + Agarose gel
6. TFA_gel_DMEM = TFA + DMEM+ Agarose gel

7. NP-F_gel = NP-F + Agarose gel
8. TFA_gel = TFA + Agarose gel

Supplementary Figure 70: Summary of samples measured in agarose gels with either NP-F or TFA.

Supplementary Table 27: Values for viscosity η, diffusion constants *D* and the derived hydrodynamic radii r_h for NP-F incubated with THP-1 cells or DMEM embedded in agarose gels (0.5 % approximately), and the corresponding standard deviation.

Sample	η [10^{-4}·kg/ms]	D [10^{-11} m^2/s]	r_h [nm]
NP-F_gel_cell_0h	8.53 ± 0.17	5.14 ± 0.62	5.23 ± 0.57
NP-F_gel_cell_24h	7.48 ± 0.14	5.99 ± 1.23	5.23 ± 1.20
NP-F_gel_DMEM	7.70 ± 0.38	3.38 ± 0.02	8.72 ± 0.05
NP-F_gel	7.67 ± 0.29	3.36 ± 0.06	8.83 ± 0.15

In Supplementary Figure 71 the comparison between the hydrodynamic radii r_h as calculated for NP-F_water, that is NP-F in water and no agarose gel (8.74 ± 0.33 nm, Supplementary Table 22), after incubation with THP-1 cells (0 and 24 hours), in the presence and absence of DMEM, all of them embedded in approximately 0.5 % agarose gels, is shown.

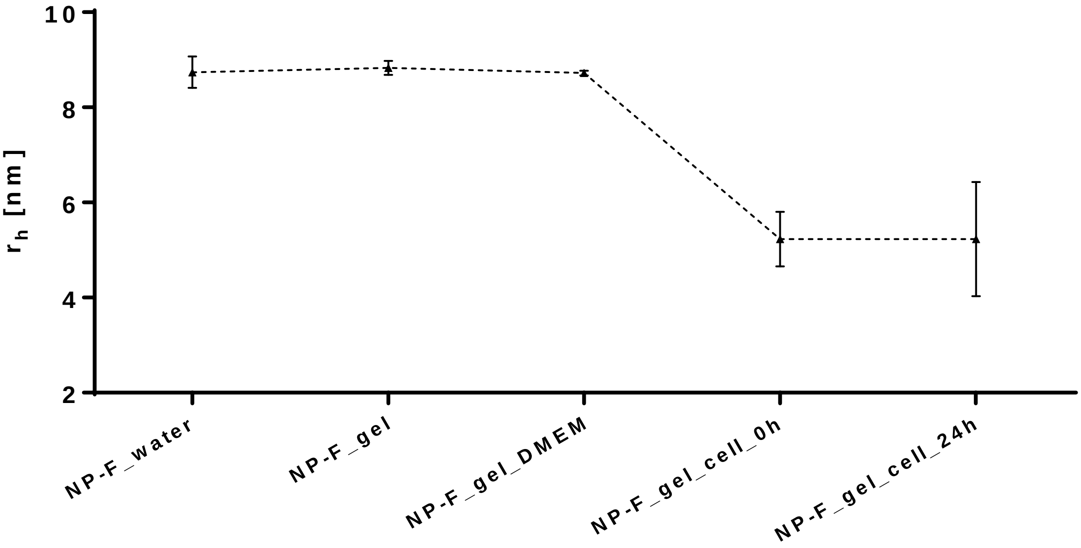

Supplementary Figure 71: Hydrodynamic radii r_h ± standard deviation for NP-F embedded in agarose gels in the presence and absence of cells.

As it can be observed, when NP-F was placed in agarose gels with or without DMEM, no size modifications were observed. However, in the presence of THP-1 cells, NPs shrank but their size remained unchanged even after 24 hour incubation. As at time point 0, *i.e.* directly after adding the NPs to cells no significant internalization of NPs by cells can be assumed, the mere presence of cells seem to affect diffusion of the NPs. Substantial shrinkage was equally observe in the presence of blood for related NPs such as NP-F/NH_2 (*cf*. Supplementary Table 20). Unravelling the resons for this will be an iminent task for the future.

Potential in vivo measurements

Ideally, a logical step forward in this research would be to measure diffusion of fluorinated NPs in vivo. We performed very preliminary ^{19}F-MRS (magnetic resonance spectroscopy) measurements in a phantom using a 11.7 T Bruker scanner with a 40 mm ^{1}H-^{19}F volumetric coil. Such phantom consisted of a NP-F/COOH solution (0.27 µM) in roughly 2 mL of water, to mimic the total volume of blood in a mouse (Supplementary Figure 72). That NP concentration corresponds to approximately 0.25 mM concentration of fluorine (0.5 µmol of total fluorine in 2 mL sample), which we estimate as the minimum amount of fluorine required to obtain a NMR diffusion measurement within a reasonable time.

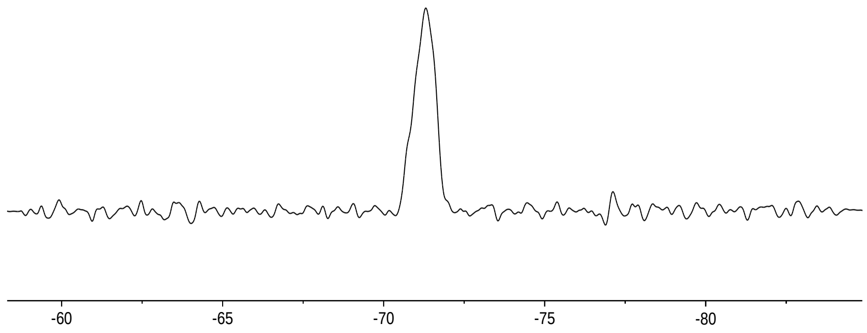

Supplementary Figure 72: ^{19}F-MRS spectrum of a phantom consisting of a 2 mL Eppendorf filled up with a NP-F/COOH solution (0.27 µM in NP and 0.25 mM in fluorine, approx. 2 mL) obtained in 3.5 min of scanning time.

In our phantom we used 0.33 mg of NP-F/COOH. According to ICP-MS data described before, 26.64 % of the total mass of the NP corresponds to gold mass. Hence, the remaining 73.36% corresponds to PEG ligands on the surface. Since 75% of the PEG ligands are HS-PEG-F, and given the very similar size of HS-PEG-F and HS-PEG-COOH (which is the remaining 25% of ligands), we can assume that 75% of the remaining mass after gold subtraction corresponds to fluorinated ligand. By dividing the mass of HS-PEG-F by its molecular weight (approximately 3300 $g \cdot mol^{-1}$) we obtain the number of HS-PEG-F mol in our phantom. Since each HS-PEG-F has 9 fluorine atoms, we can directly estimate the amount of fluorine present:

0.33 x 0.75 x (100-26.64) = 0.182 mg of HS-PEG-F
0.182 / 3300 = $5.5 \cdot 10^{-5}$ mmol of HS-PEG-F
$5.5 \cdot 10^{-5}$ x 9 = $5 \cdot 10^{-4}$ mmol = 0.5 µmol of F
$5 \cdot 10^{-4}$ mmol / 2 mL = $2.5 \cdot 10^{-4}$ M = 0.25 mM of F in a 2 mL sample.

Hence, one could in principle reach the desired fluorine amount, i.e. 0.25 mM in fluorine which corresponds to 0.27 µM in gold NP, with a 2.7 µM NP-F/COOH solution via tail vein injection (200 µL bolus):

$2.7 \cdot 10^{-7}$ mmol/mL x 2 mL = $5.4 \cdot 10^{-7}$ mmol Au NP are needed
$5.4 \cdot 10^{-7}$ mmol / 0.2 mL = $2.7 \cdot 10^{-6}$ M = 2.7 µM (0.2 mL injection volume)

Thus *in vivo* detection of NP hydrodynamic radii with the herein described method may in principle be feasible.

Supplementary References

1 Szabo, D., Mohl, J., Balint, A. M., Bodor, A. & Rabai, J. Novel generation ponytails in fluorous chemistry: Syntheses of primary, secondary, and tertiary (nonafluoro-tert-butyloxy)ethyl amines. *Journal of Fluorine Chemistry* **127**, 1496-1504 (2006).

2 Lin, C.-A. J. *et al.* Design of an Amphiphilic Polymer for Nanoparticle Coating and Functionalization. *Small* **4**, 334-341 (2008).

3 del_Pino, P. *et al.* Basic Physicochemical Properties of Polyethylene Glycol Coated Gold Nanoparticles that Determine Their Interaction with Cells. *Angew Chem Int Ed Engl* **55**, 5483–5487 (2016).

4 Schneider, C. A., Rasband, W. S. & Eliceiri, K. W. NIH Image to ImageJ: 25 years of image analysis. *Nature Methods* **9**, 671-675 (2012).

5 Rasband, W. S. (U. S. National Institutes of Health, Bethesda, Maryland, USA, https://imagej.nih.gov/ij/, 1997-2016).

6 Soliman, M. G., B, B. P., Parak, W. J. & Pino, P. d. Phase transfer and polymer coating methods toward improving the stability of metallic nanoparticles for biological applications. *Chemistry of Materials* **27**, 990-997 (2015).

7 del Pino, P. *et al.* Basic Physicochemical Properties of Polyethylene Glycol Coated Gold Nanoparticles that Determine Their Interaction with Cells. *Angewandte Chemie-International Edition* **55**, 5483-5487 (2016).

8 Hua, X. Y. & Rosen, M. J. Dynamic Surface-Tension of Aqueous Surfactant Solutions .1. Basic Parameters. *Journal of Colloid and Interface Science* **124**, 652-659 (1988).

9 Tan, G. *et al.* Conjugation of Polymer-Coated Gold Nanoparticles with Antibodies—Synthesis and Characterization. *Nanomaterials* **5**, 1297-1316 (2015).

10 Bradford, M. M. A rapid and sensitive method for the quantitation of microgram quantities of protein utilizing the principle of protein-dye binding. *Analytical Biochemistry* **72**, 248-254 (1976).

11 Pellegrino, T., Sperling, R. A., Alivisatos, A. P. & Parak, W. J. Gel Electrophoresis of Gold-DNA Nanoconjugates. *Journal of Biomedicine and Biotechnology* **2007**, 9, doi:10.1155/2007/26796 (2007).

12 Swan, I. *et al.* Sample convection in liquid-state NMR: Why it is always with us, and what we can do about it. *Journal of Magnetic Resonance* **252**, 120-129 (2015).

13 Stejskal, E. O. & Tanner, J. E. Spin Diffusion Measurements: Spin Echoes in the Presence of a Time-Dependent Field Gradient. *Journal of Chemical Physics* **42**, 288 (1965).

14 Wu, D. H., Chen, A. D. & Johnson, C. S. An Improved Diffusion-Ordered Spectroscopy Experiment Incorporating Bipolar-Gradient Pulses. *Journal of Magnetic Resonance Series A* **115**, 260-264 (1995).

15 Röcker, C., Pötzl, M., Zhang, F., Parak, W. J. & Nienhaus, G. U. A Quantitative Fluorescence Study of Protein Monolayer Formation on Colloidal Nanoparticles. *Nat. Nanotechnol.* **4**, 577-580 (2009).

16 del_Pino, P. *et al.* Protein corona formation around nanoparticles-from the past to the future. *Materials Horizons* **1**, 301-313 (2014).

17 Michelena, O. *et al.* Novel fluorinated ligands for gold nanoparticle labelling with applications in F-19-MRI. *Chemical Communications* **53**, 2447-2450 (2017).
